# Consequences of COVID-19 infection on educators’ executive functions: A systematic review and meta-analysis protocol

**DOI:** 10.1371/journal.pone.0350156

**Published:** 2026-10-14

**Authors:** Leonardo Mateus Teixeira de Rezende, Kaio Vinicius C. Silva, Ariel Cuadro, Vicente Miñana Signes, Matias Noll, Priscilla Rayanne e Silva

**Affiliations:** 1 Instituto Federal Goiano, Ceres, Goiás, Brazil; 2 Faculty of Nutrition, Federal University of Goiás (UFG), Goiânia, Goiás, Brazil; 3 Universidad Católica Del Uruguay, Motenvideo, Uruguay; 4 Education Faculty, University of Valencia, Valencia, Spain; Universidade Federal de Goias, BRAZIL

## Abstract

The coronavirus disease (COVID-19) pandemic has had a direct impact on social life and workplace, impairing work performance due to social and health difficulties, including cardiorespiratory issues, vascular damage, and cognitive deficits. Moreover, infection can impair cognitive functioning, particularly executive functions, which are essential skills for developing activities that require the application and management of concepts, such as those required in a educator’s work.

**Objective:**

This study aims to present the methodological framework and predefined criteria that will guide a systematic review and meta-analysis investigating the effects of COVID-19 infection on educators’ executive functions.

**Method:**

We will search seven databases—Web of Science Core Collection, Scopus, MEDLINE/PubMed via the National Library of Medicine interface, Cochrane Library, Latin American and Caribbean Health Sciences, Embase, ERIC, and SPORTDiscus via the EBSCOhost interface. Data extraction and analysis will be performed according to the guidelines of the Preferred Reporting Items for Systematic Reviews and Meta-Analysis Protocols. The entire process will be carried out by two independent reviewers, with any discrepancies resolved by a third reviewer. The eligibility criteria followed the PECO strategy. To be included, articles will have to be: i) original published, ii) studies with educators, and iii) studies that analyzed executive functions. Conversely, exclusion criteria will be: i) duplicates, ii) literature reviews, and iii) studies with retractions. If the search strategy yields a sufficient number of methodologically comparable studies, meta-analytic statistical methods will be applied, including meta-analysis or meta-regression, sensitivity, consistency, precision, and certainty of evidence analyses.

**Conclusion:**

This systematic review is expected to deepen understanding of COVID-19’s impact on educator’s health, with a focus on analyzing executive functions.

## Introduction

The coronavirus disease (COVID-19) has led to significant challenges in the world, affecting various aspects of daily life [1]. While much attention has been given to the respiratory and systemic effects of the virus, increasing evidence suggests that COVID-19 also affects the central nervous system (CNS) [2]. There are also reports of psychosocial issues, leading to disorders such as anxiety and depression [3,4]. Therefore, we are aware that the extent of the virus infection and the pandemic context experience have broad repercussions. This suggests the need for greater in-depth analysis of the long-term consequences for the population.

Aiming to broaden understanding, a biopsychosocial approach is necessary. From a biological perspective, COVID-19 has the potential to lead to neuroinflammation—commonly referred to as a “cytokine storm” [5], hypoxia [6], vascular damage [7] and, potentially leading to neuronal damage and cognitive deficits [8]. The psychological aspect is also affected. Studies point to the incidence of feelings of tiredness and stress [9,10], the “brain fog” [11] and mental confusion [12]. The social consequences have also been devastating. The pandemic has forced a routine break, distancing and reclusion, and work dynamics changes, generating problems such as anxiety and family financial crises, for example [13]. Indeed, previous research has linked such impairments to a decline in executive functions (EFs) [14–16]. These effects can even contribute to neuronal injury and cognitive deficits [17,18].

The impact on the CNS occurs as SARS-CoV-2 is a neurotrophic virus, that is, has attraction for nerve cells [19]. The virus reaches the CNS through sensory and motor pathways, for example, the olfactory nerve pathway. When it reaches the CNS, it triggers demyelination of nerve fibers and inflammatory processes [19]. These trigger a series of neuropsychiatric disorders. In the short-term, complications such as cerebrovascular events, headache, encephalopathies, anosmia, among others, are observed [20–23]. The literature has already become aware of the long-term infection consequences. Symptoms of inattention and dysexecutive dysfunction are common [24]. There are also reports of frontotemporal hypoperfusion, as well as structural brain abnormalities, mainly in the thalamic and temporal regions [25]. Demyelination also causes an information processing reduction, directly affecting performance in tasks related to the use of central executive functions [2].

Among the cognitive domains potentially affected, executive functions (EFs) have emerged as a particular concern [7–9]. These high-order cognitive processes, also known as cognitive control, include abilities essential for regulating thoughts and behaviors, such as maintaining attention, organizing tasks, problem-solving, and adapting to new information. The literature has identified three central EFs [26]: i) inhibitory control (i.e., managing inappropriate behaviors [17,18] and maintaining selective attention [26,27]), ii) working memory (i.e., retention and manipulation of information) [28], and ii) cognitive flexibility (i.e., adaptation to new rules [18] and perspectives [10]. From these core functions, more complex skills such as planning, decision-making, problem-solving, and reasoning emerge [18]. For the purposes of this review, executive functions will be operationally defined as cognitive processes assessed through validated instruments or structured tasks that explicitly measure at least one of the following domains: inhibitory control, working memory, or cognitive flexibility [29–31]. These skills are important for teaching activities as they allow the establishment of relationships between ideas, the integration of new information with existing knowledge, and the ability to remember sequences of events.

For educators, these EFs are critical to the performance of professional duties. Teaching demands the integration of information, multitasking, responsiveness to dynamic classroom environments (including online formats), and the ability to make pedagogical decisions under time constraints. Cognitive impairments in these areas may, therefore, directly compromise educational quality and teacher well-being. A report explained that COVID-19 led to impairments in executive function in 36% of cases [32]. Other studies have shown similar results, indicating that COVID-19 infection may lead to disorders related to headache, confusion and attention difficulties [21,23].

In addition to fear, COVID-19 has created unexpected and high work demands. This overloading teachers and imposing a routine that has resulted in a poor well-being [33]. Studies show that this disruption in well-being affects cognitive abilities, resulting in low scores in EFs analyses [33,34].

It’s worth mentioning that systematic reviews related to this topic have already been published [35-36]. Korzeniowski sought to understand the impacts of COVID-19 on children’s executive functions and found significant impacts on mental health, well-being, and quality of life [35]. Another relevant study reviewed the neurocognitive profile of people infected with the virus [37]. The authors concluded that executive functions are significantly impaired in people infected with long-term COVID, resulting in reduced performance on tasks that require the use of cognitive capacity. A third systematic review sought to understand the relationship between COVID-19 infection and the incidence of cognitive deficits, finding a high frequency of decline in cognitive capacity after infection [38]. Finally, a systematic review evaluated the cognitive effects of the infection on adults. The study concluded that significant impairments exist, but indicated that the pathological mechanisms underlying this are still unknown [36].

Despite initial studies suggesting a connection, gathering more solid evidence on whether COVID-19 affects educators’ EFs is important. To the best of our knowledge, no systematic review has addressed this issue [39,40]. The guiding research question was the following: “Does COVID-19 affect teachers’ EFs in the long term?” This knowledge can enhance our scientific understanding of the topic because EFs represent an essential element for teaching activities. Thus, The objective of this protocol is to describe the methodological procedures, eligibility criteria, search strategy, and analytical approach that will be used to conduct a systematic review and meta-analysis examining the consequences of COVID-19 infection on educators’ executive functions.

## Materials and methods

### Protocol and registration

This systematic review protocol was written based on the recommendations of the Preferred Reporting Items for Systematic Reviews and Meta-analyses for Protocols (PRISMA-P) [41]. After defining the research problem, a preliminary search will be conducted in the databases to identify studies that form the basis of the research. However, the full systematic search will be conducted once the protocol is published.

The study protocol was registered in the International Prospective Register of Systematic Reviews (PROSPERO) (CRD42024558019). If there is a need for modifications to ongoing research, these changes will be reported to PROSPERO.

### Search strategy and databases

A systematic review will be conducted to gather bibliographic data from the following databases: Web of Science Core Collection, Scopus, MEDLINE/PubMed via the National Library of Medicine interface, Cochrane Library, Latin American and Caribbean Health Sciences, Embase, ERIC, and SPORTDiscus via the EBSCOhost interface. The electronic database searches will be conducted on 10 April 2026, following acceptance of this protocol, to ensure methodological transparency and to capture the most up-to-date evidence available at the time of the review. Additionally, the search strategy was complemented by screening the reference lists of the included studies.

This systematic review employed the following Medical Subject Headings terms as the primary search terms: “COVID-19” and “executive function”. The search strategy will comprise three blocks that refer to the public (educators), exposure (COVID-19), and outcome (executive functions), as shown in Table 1.

**Table 1:** Search strategy organized into blocks.

Search	Blocks with search terms
#1	(teacher OR teach* OR professor OR professors OR advisor OR advisors OR lecturer OR lecturers OR “mentor” OR mentors OR instructor OR instructors OR pedagogue OR pedagogues OR schoolteacher OR schoolteachers OR preceptor OR preceptors OR educator OR educators OR educationist OR educationists OR tutor OR tutors)
#2	(“COVID-19” OR “COVID19” OR “coronavirus” OR “sars” OR “2019-nCoV” OR “CoV-2” OR “long covid” OR “omicron” OR “delta” OR “severe acute respiratory syndrome”)
#3	(“executive function*” OR “executive control” OR cognition OR “cognitive function*” OR “cognitive performance” OR “cognitive inhibition” OR “cognitive flexibility” OR “working memory” OR “frontal lobe function*” OR “inhibitory control” OR “working memory” OR “cognitive skill*” OR “cognitive control” OR “mental function*”)
#4	(#1) AND (#2) AND (#3)

The titles, abstracts, and keywords in the databases will be searched. The metadata extracted from the databases adhered to the Preferred Reporting Items for Systematic Reviews and Meta-Analyses (PRISMA-S) search checklist [42].

Search results will be exported from the databases in RIS format, stored and organized in Mendeley (reference manager), and subsequently imported into Rayyan software (http://rayyan.qcri.org) a web and mobile app for systematic reviews- for screening and selection [43]. It is important to emphasize that references from other systematic reviews will also be screened. The raw search data will be made available upon request, and all records will be retained indefinitely, in accordance with institutional data management policies. The reviewers will carry out the analyses in parallel and using the “blind” feature. Subsequently, the data from the selected studies will be extracted independently by each reviewer.

### Eligibility criteria

The eligibility criteria for this study were determined based on population, exposure, comparison, and outcome (PECO) (Table 2) [44,45]. Accordingly, the following parameters are established: population (P), educators; exposure (E), have been infected with COVID-19; and outcome (O), executive function.

**Table 2:** Eligibility criteria for study selection according to the population, exposure, comparison, and outcome (PECO) framework.

PECO	Inclusion	Exclusion
Population	Adult individuals (>18 years) who work as a teacher/educator at any educational levels	individuals diagnosed with psychiatric illnesses
Exposure	COVID-19 infection	Not exposed to COVID-19 infection
Outcomes	Analysis of the executive functions of teachers who were infected with COVID-19	Studies that used non-validated methods to analyze executive functions
		Self-reported executive functioning
		Studies that analyzed executive functions outside the context of COVID-19
Study design	Cross-sectional, longitudinal; randomized and non-randomized studies	Literature reviews; studies with retractions

This systematic review will include articles published after the onset of the COVID-19 pandemic (March 11, 2020). To be considered eligible, articles have to meet the following inclusion criteria: i) original published articles and peer-reviewed studies, ii) studies with educators, and iii) studies that analyzed executive functions. Conversely, the exclusion criteria were as follows: i) duplicates (studies published in more than one journal were carefully reviewed to confirm elimination) [46], ii) literature reviews, and iii) studies with retractions. Retraction was identified using the Scite tool [42].

### Reviewer training

Before beginning the screening process, researchers involved in eligibility assessments will undergo training focused on the study eligibility criteria [47-48]. The researchers will first receive specific instructions on how to use the Rayyan software. Following which they will assess the eligibility of 50 references (i.e., abstracts and titles) to standardize the screening process. Additionally, a specific training session will be held to standardize the assessment of the risk of bias.

## Data extraction, synthesis, and analysis

After extracting the data from the databases, it will be added to the Rayyan online software. This tool allows the peer review process to be conducted by independent reviewers (LM and SM) who begin by reading the titles and abstracts to apply the eligibility criteria. Disagreements will be resolved by consensus with a third researcher (PS). The selection process is illustrated in the flowchart (Fig 1) [49].

**Fig 1:**
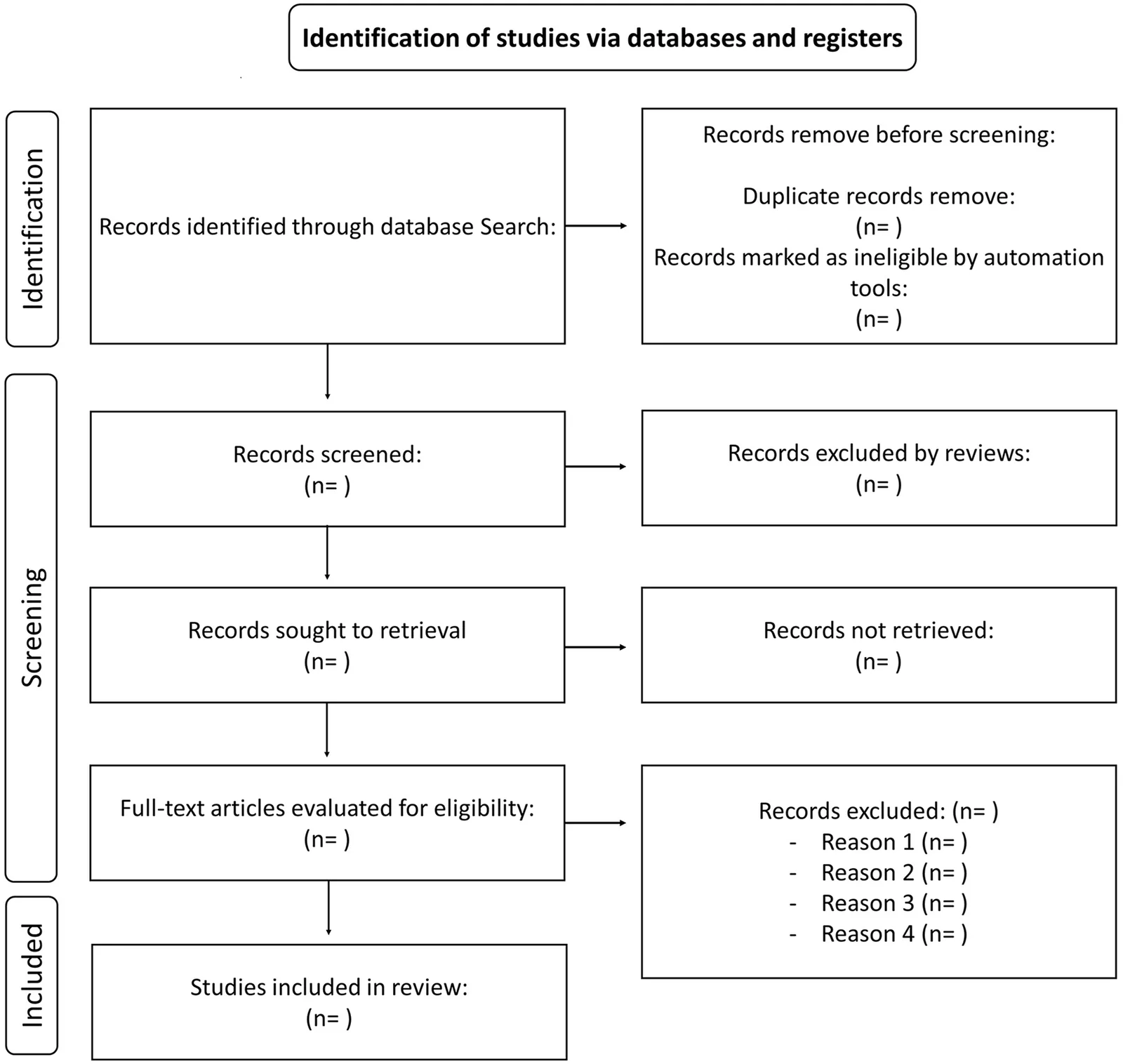
PRISMA 2020 flow diagram for the identification, screening, and inclusion of studies in this review.

The study selection process will be conducted in three sequential phases: title screening, abstract screening, and full-text screening. All phases will be performed independently by two reviewers (LM and SM), and any disagreements will be resolved through discussion and, when necessary, adjudicated by a third reviewer (PS).

In the first phase, study titles will be screened against the predefined eligibility criteria. In the second phase, abstracts of the selected titles will be reviewed by the same two independent reviewers using the same inclusion and exclusion criteria. In the third phase, the full texts of potentially eligible studies will be retrieved and examined to confirm final inclusion [50]. All three screening stages will be conducted independently and blindly between LM and SM.

A spreadsheet will be created to include all fields presented in Table 3 for data extraction. Two independent reviewers will evaluate and extract the data (LM and SM). Any discrepancies will be addressed by consensus with a third reviewer (PS). Data synthesis and analysis will also be performed by LM and SM, under the methodological supervision of PS. Depending on the volume and quality of the included studies, both qualitative synthesis and, where appropriate, meta-analytic methods (e.g., meta-analysis or meta-regression) will be applied. Quality appraisal and risk of bias assessments will be essential to inform the statistical approach and interpret the findings in alignment with the review’s objectives.

**Table 3:** Data extraction from the included studies.

Author, year, and country of publication	Study design	Intervention used	Sample	Instrument used	Statistical analysis	Main results
						
						
						
						

Descriptive and content analyses of the results will also be conducted based on the impact of COVID-19 on educators’ executive functions. The extracted data will include quantitative indicators such as means, standard deviations, and effect sizes related to executive function performance (e.g., working memory, cognitive flexibility, inhibitory control), as well as qualitative themes derived from narrative reports or interviews. Indices such as test scores, neuropsychological assessments, and self-reported measures will be used to categorize and synthesize findings.

The WebPlotDigitizer will be used to collect the data available in the graphs [51]. If any data are missing from the primary studies, the authors will be contacted by email up to three times over a period of four weeks. If no response is received after this period, the study will be included in the review with the available data, and the missing information will be acknowledged as a limitation. After collecting all the data, the percentage of agreement and Cohen’s kappa will be calculated to evaluate the accuracy of each stage of the data extraction process. Cohen’s kappa coefficient will be interpreted as a measure of agreement between evaluators, ranging from 0 to 1, where values closer to 1 indicate high agreement beyond chance, and values closer to 0 indicate low or no agreement [52,53].

### Risk of bias

The risk of bias will be assessed by two independent reviewers, and any disagreements will be resolved by consulting a third reviewer. Given the potential methodological heterogeneity of the included studies, validated tools will be applied according to the specific study design. For observational studies, the Joanna Briggs Institute (JBI) critical appraisal tools will be used; for randomized controlled trials, the Cochrane Risk of Bias Tool (RoB 2.0); for qualitative studies, the JBI Checklist for Qualitative Research; and for mixed-methods studies, the Mixed Methods Appraisal Tool (MMAT).

In cases where the Downs and Black checklist is appropriate—particularly for quantitative studies with both randomized and non-randomized elements—it may also be applied [54]. A quality score will be calculated for each study and expressed as a percentage of the maximum possible score for that design. Studies scoring 70% or higher will be considered to have a low risk of bias [55].

### Main meta-analyses and/or meta-regressions

The primary outcome will be post-COVID-19 executive function performance in educators, assessed through validated neuropsychological tests (e.g., working memory, inhibitory control, cognitive flexibility measures). For continuous outcomes, effect sizes will be calculated as standardized mean differences (Hedges’ g) with 95% confidence intervals (95% CI). Statistical significance will be defined as p < 0.05 (two-tailed). Effect size magnitude will be interpreted according to Cohen’s criteria (0.20 = small, 0.50 = moderate, 0.80 = large) [56].

For dichotomous outcomes, pooled estimates will be expressed as odds ratios (OR) or risk ratios (RR) with 95% CI. When adjusted estimates are available, the most fully adjusted model will be extracted [56].

A random-effects model (DerSimonian and Laird estimator) will be used. Between-study heterogeneity will be quantified using I^2^ (25% low, 50% moderate, 75% high) and Cochran’s Q test (p < 0.10 indicating significant heterogeneity) [57]. Subgroup analyses and meta-regression will be conducted when k ≥ 10 studies are available per covariate [58].

All analyses will be conducted in R (metafor and meta packages). Methodological decisions are based on established meta-analytic guidelines [56].

### Sensitivity analysis

Sensitivity analyses will be conducted to evaluate the robustness and reliability of the meta-analytic results. The primary sensitivity analysis will use a leave-one-out approach, systematically excluding each individual study one at a time to assess its influence on the overall pooled effect size and confidence intervals. This will identify any studies disproportionately affecting the results or driving the observed effect [59].

Additionally, sensitivity analyses will exclude studies classified as having a high risk of bias, based on the predefined criteria of each risk of bias assessment tool applied (e.g., studies scoring below 70% on the Downs and Black scale or rated as “high risk” on the Cochrane RoB 2.0 tool). The impact of excluding these studies on pooled estimates will be reported. Further sensitivity analyses will be restricted to studies providing complete datasets for the primary outcomes (executive functions) to ensure comparability and reduce potential bias from missing data.

All sensitivity analyses will be fully described in the results section, including the specific studies excluded, changes in effect sizes, confidence intervals, and heterogeneity statistics (I^2^). Any substantial changes in the direction or magnitude of the effect will be explicitly discussed. Sensitivity analyses will be performed after the initial meta-analysis and will also be conducted whenever significant heterogeneity (I^2^ > 50%) or risk of bias concerns are identified.

### Consistency analysis and precision analysis

Each analyzed dataset will be evaluated for consistency. Thus, we will use the *Q* statistic to assess consistency, with P ≤ 0.05 indicating the presence of significant heterogeneity. Additionally, the I^2^ statistic will be employed to detect any heterogeneity between effect estimates, with 0–25% indicating “low heterogeneity,” 25–50% indicating “moderate heterogeneity,” and more than 50% indicating “high heterogeneity” [57]. Finally, to assess the precision of each meta-analysis, the power of all analyses will be calculated [60].

### Publication bias

The analysis included a check for publication bias across all meta-analyzed datasets [61]. We will begin by generating funnel plots that display the effect sizes of individual studies on the x-axis against the study size or precision on the y-axis [62]. Visual asymmetry in these plots would suggest publication bias [62].

Moreover, we will perform a “trim and fill” analysis in search of missing studies, which should be illustrated in the funnel plot [34,35,37]. This method identifies potentially missing studies using funnel plots and provides an adjusted meta-analysis [63]. Finally, Egger’s test will be used to ensure greater objectivity in detecting publication bias [64].

### Certainty of evidence

The GRADE (Grading of Recommendations, Assessment, Development, and Evaluations) Working Group method will be used to assess the certainty of the evidence. To evaluate the risk of bias, the certainty of evidence will be downgraded by two levels if 50–75% of the studies are rated as having a “low risk of bias” and by one level if less than 50% of the studies are considered to have a “low risk of bias.” If the power of detection of the analysis is less than 90%, the certainty will be reduced by one level, and if the power is less than 80%, it will be reduced by two levels.

The certainty of the evidence will be downgraded by one level for “moderate heterogeneity” and by two levels for “high heterogeneity.” The presence of publication bias, as indicated by the Egger test result (P < 0.05), lowers certainty by one level. Additionally, when indirect evidence is involved, such as by extrapolating findings to populations not specified in the study, the certainty is downgraded by one level. Despite these downgrading criteria, the certainty of the evidence may increase if there is a “large” or “very large” effect size [60]. This method classifies each meta-analysis result into one of four levels: high (⨁⨁⨁⨁), moderate (⨁⨁⨁), low (⨁⨁), or very low (⨁). The GRADE assessments will be conducted using the GRADEpro GDT software (https://gradepro.org).

## Discussion

This systematic review protocol aims to understand the impact of the COVID-19 pandemic on educators’ EFs. The COVID-19 pandemic has had short-, medium-, and long-term consequences, many of which remain unknown [39]. By systematically examining these effects, this protocol provides critical insights that can inform the development of interventions to support educators’ mental health and enhance the quality of student teaching in post-pandemic times.

EFs are fundamental to an educator’s professional performance and play a central role in the execution of a series of recurring tasks in this type of work activity [65]. Therefore, understanding the impact of COVID-19 on these variables is highly valuable for a class of workers who perform socially and economically relevant roles. Furthermore, understanding the long-term impact of the virus on the EFs of education professionals will provide the necessary support for creating intervention plans aimed at minimizing consequences, as well as for preventing new COVID-19 infections and new pandemic episodes.

This systematic review protocol has several strengths that could contribute to improving educators’ activities, health, and quality of life. This field is still poorly explored and requires the attention of the scientific community. Gathering these data will allow the planning of public-private interventions aimed at mitigating the consequences of COVID-19 on the professional activity of educators, which certainly has an impact on the quality of young students’ education.

Using an appropriate and robust methodology guarantees data validity and reliability. For this purpose, PRISMA-P [41] and PRISMA [49] will be used as reference methods with the PECO strategy for organizing and managing database searches. Furthermore, all reviewer training guarantees fairness, assertiveness, and efficiency in the peer review process.

A potential limitation of this protocol is the possibility of retrieving a limited number of studies after applying the database search strategies, which may restrict the breadth of the synthesis. Nonetheless, even a small number of studies can provide valuable insights and highlight existing gaps in the literature that warrant further investigation [66,67]. Another limitation that may arise is the great variability in the characteristics of the population used as a sample, leading to heterogeneity, which makes the synthesis and analysis of results more complex. One proposal to alleviate this issue is to classify the participants into subgroups to perform statistical analyses.

It is worth noting that our findings will be limited to educators and cannot be generalized to all populations. Subsequent studies should observe specific classes of educators and other populations.

## Conclusion

This protocol aims to establish the appropriate methodological criteria for later carrying out the systematic review and meta-analysis to understand the impact of a serious global event, the COVID-19 pandemic, on educators’ EFs. The findings are expected to contribute to the development of interventions aimed at promoting educators’ cognitive health, particularly executive functions, which are closely related to planning, attention, emotional regulation, and decision-making. By supporting these capacities, such interventions may indirectly favor improvements in educators’ mental health and professional well-being, potentially influencing the overall quality of teaching and the educational environment.
