# Supplementary material for: Consequences of COVID-19 infection on educators’ executive functions: A systematic review and meta-analysis protocol
Source: PLoS One. 2026 Oct 14;21(10):e0350156. doi: 10.1371/journal.pone.0350156 (PMC13667799; doi:10.1371/journal.pone.0350156)

PLOS ONE

Consequences of COVID-19 infection on professors’ executive functions: a systematic review protocol

--Manuscript Draft--

**Manuscript Number:**	PONE-D-25-03730
**Article Type:**	Study Protocol
**Full Title:**	Consequences of COVID-19 infection on professors’ executive functions: a systematic review protocol
**Short Title:**	Consequences of COVID-19 infection on professors’ executive functions: a systematic review protocol
**Corresponding Author:**	Leonardo Mateus Teixeira de Rezende, PhD<br>Instituto Federal Goiano<br>Ceres, BRAZIL
**Keywords:**	Executive functions; COVID-19; systematic review; Public Health; educators.
**Abstract:**	The coronavirus disease (COVID-19) pandemic has had a direct impact on social life and workplace, impairing work performance due to social and health difficulties, including cardiorespiratory issues, vascular damage, and cognitive deficits. Moreover, infection can impair cognitive functioning, particularly executive functions, which are essential skills for developing activities that require the application and management of concepts, such as those required in a professors’ work.<br><br>Objective<br><br>This systematic review aims to explore the effects of COVID-19 on professors’ executive functions.<br><br>Method<br><br>We will search seven databases—Web of Science Core Collection, Scopus, MEDLINE/PubMed via the National Library of Medicine interface, Cochrane Library, Latin American and Caribbean Health Sciences, Embase, ERIC, and SPORTDiscus via the EBSCOhost interface. Data extraction and analysis will be performed according to the guidelines of the Preferred Reporting Items for Systematic Reviews and Meta-Analysis Protocols. The entire process will be carried out by peers, that is, by the work of two independent reviewers, with any discrepancies resolved by a third reviewer. If the results allow, meta-analytic statistical methods will be applied, including meta-analysis or meta-regression, sensitivity, consistency, precision, and certainty of evidence analyses. Discussion: This systematic review is expected to deepen understanding of COVID-19’s impact on professors' health, with a focus on analyzing executive functions. Additionally, it will allow for the development of strategies aimed at implementing protective measures and mitigating post-infection effects.
**Order of Authors:**	Leonardo Mateus Teixeira de Rezende, PhD
	Kaio Vinicius C Silva
	Ariel Cuadro
	Vicente Miñana Signes
	Matias Noll
	Priscilla Rayanne e Silva Noll
**Additional Information:**	
**Question**	**Response**
**Financial Disclosure**	The author(s) received no specific funding for this work.

Powered by Editorial Manager® and ProduXion Manager® from Aries Systems Corporation

Enter a financial disclosure statement that describes the sources of funding for the work included in this submission. Review the submission guidelines for detailed requirements. View published research articles from *PLOS ONE* for specific examples.<br><br>This statement is required for submission and **will appear in the published article** if the submission is accepted. Please make sure it is accurate.<br><br>**Funded studies**<br>Enter a statement with the following details:<br>• Initials of the authors who received each award<br>• Grant numbers awarded to each author<br>• The full name of each funder<br>• URL of each funder website<br>• Did the sponsors or funders play any role in the study design, data collection and analysis, decision to publish, or preparation of the manuscript?<br><br>Did you receive funding for this work?	
**Competing Interests**<br><br>Use the instructions below to enter a competing interest statement for this submission. On behalf of all authors, disclose any competing interests that could be perceived to bias this work—acknowledging all financial support and any other relevant financial or non-financial competing interests.<br><br>This statement is required for submission and **will appear in the published article** if the submission is accepted. Please make sure it is accurate and that any funding sources listed in your Funding Information later in the submission form are also declared in your Financial Disclosure statement.<br><br>View published research articles from	NO authors have competing interests.

PLOS ONE for specific examples.<br><br>**NO authors have competing interests**<br><br>Enter: *The authors have declared that no competing interests exist.*<br><br>**Authors with competing interests**<br><br>Enter competing interest details beginning with this statement:<br><br>*I have read the journal's policy and the authors of this manuscript have the following competing interests: [insert competing interests here]*<br><br>* typeset	
**Ethics Statement**<br><br>Enter an ethics statement for this submission. This statement is required if the study involved:<br><br>• Human participants<br>• Human specimens or tissue<br>• Vertebrate animals or cephalopods<br>• Vertebrate embryos or tissues<br>• Field research<br><br>Write "N/A" if the submission does not require an ethics statement.<br><br>General guidance is provided below. Consult the submission guidelines for detailed instructions. **Make sure that all information entered here is included in the Methods section of the manuscript.**	N/A.

**Format for specific study types**<br><br>**Human Subject Research (involving human participants and/or tissue)**<br>• Give the name of the institutional review board or ethics committee that approved the study<br>• Include the approval number and/or a statement indicating approval of this research<br>• Indicate the form of consent obtained (written/oral) or the reason that consent was not obtained (e.g. the data were analyzed anonymously)<br><br>**Animal Research (involving vertebrate animals, embryos or tissues)**<br>• Provide the name of the Institutional Animal Care and Use Committee (IACUC) or other relevant ethics board that reviewed the study protocol, and indicate whether they approved this research or granted a formal waiver of ethical approval<br>• Include an approval number if one was obtained<br>• If the study involved *non-human primates*, add *additional details* about animal welfare and steps taken to ameliorate suffering<br>• If anesthesia, euthanasia, or any kind of animal sacrifice is part of the study, include briefly which substances and/or methods were applied<br><br>**Field Research**<br><br>Include the following details if this study involves the collection of plant, animal, or other materials from a natural setting:<br>• Field permit number<br>• Name of the institution or relevant body that granted permission	
**Data Availability**<br><br>Authors are required to make the data underlying their research findings fully available, without restriction.<br><br>If the manuscript reports pilot or preliminary results, the data underlying those results must be made available at the time of publication. A Data Availability	Yes - Results are reported and the data are fully available

Powered by Editorial Manager® and ProduXion Manager® from Aries Systems Corporation

Statement describing where the data can be found is required at submission. Your answers to this question constitute the Data Availability Statement and will be published in the article, if accepted. When legal or ethical requirements prohibit public data sharing, authors must indicate how researchers may obtain access to the data. See the PLOS Data Policy and FAQ for detailed information.<br><br>**Important:** Stating 'data available on request from the author' is not sufficient. If your data are only available upon request, select 'No' for the first question and explain your situation in the text box.<br><br>For protocols without pilot or preliminary data, authors are strongly encouraged to state how they plan to share research data from their study when it is completed or published.<br><br>If your study includes pilot data, will they be made fully available without restriction?	
**Describe where the data may be found in full sentences. If you are copying our sample text, replace any instances of XXX with the appropriate details.**<br><br>• If **no pilot data are reported** in the manuscript, please enter the following: *No datasets were generated or analysed during the current study. All relevant data from this study will be made available upon study completion.*<br>• If the manuscript reports pilot data **held in a public repository**, include URLs, accession numbers or DOIs. If this information will only be available after acceptance, indicate this by ticking the box below. For example: *All XXX files are available from the XXX database (accession number(s) XXX, XXX.).*<br>• If the manuscript reports pilot data contained **within the manuscript and/or**	The protocol is registered on the PROSPERO platform.<br><br>CRD42024558019

**Supporting Information files**, enter the following:<br>*All relevant data are within the manuscript and its Supporting Information files.*<br>• If neither of these applies but you are able to provide **details of access elsewhere**, with or without limitations, please do so. For example:<br><br>*Data cannot be shared publicly because of [XXX]. Data are available from the XXX Institutional Data Access / Ethics Committee (contact via XXX) for researchers who meet the criteria for access to confidential data.*<br><br>*The data underlying the results presented in the study are available from (include the name of the third party and contact information or URL).*<br>• This text is appropriate if the data are owned by a third party and authors do not have permission to share the data.<br><br>For study protocols with data management or sharing plans, authors are encouraged to briefly describe in their Data Availability Statement how data they generate will be made accessible when the study is completed. For example:<br>• Deidentified research data will be made publicly available when the study is completed and published.<br><br>* typeset	
Additional data availability information:	

Manuscript Click here to access/download;Manuscript;Manuscript.docx

Consequences of COVID-19 infection on professors' executive functions: a systematic review protocol

Leonardo Mateus Teixeira de Rezende[1], Kaio Vinicius C Silva[2], Ariel Cuadro[3], Vicente Miñana Signes[4], Matias Noll[1] e Priscilla Rayanne e Silva Noll[1].

Affiliation:

[1] Instituto Federal Goiano, Ceres, Goiás, Brazil.

[2] Faculty of Nutrition, Federal University of Goiás (UFG), Goiânia, Goiás, Brazil;

[3] Universidad Católica Del Uruguay, Motenvideo, Uruguay.

[4] Education Faculty, University of Valencia, Valencia, Spain.

Abstract

The coronavirus disease (COVID-19) pandemic has had a direct impact on social life and workplace, impairing work performance due to social and health difficulties, including cardiorespiratory issues, vascular damage, and cognitive deficits. Moreover, infection can impair cognitive functioning, particularly executive functions, which are essential skills for developing activities that require the application and management of concepts, such as those required in a professors' work.

Objective: This systematic review aims to explore the effects of COVID-19 on professors' executive functions.

Method: We will search seven databases—Web of Science Core Collection, Scopus, MEDLINE/PubMed via the National Library of Medicine interface, Cochrane Library, Latin American and Caribbean Health Sciences, Embase, ERIC, and SPORTDiscus via the EBSCOhost interface. Data extraction and analysis will be performed according to the guidelines of the Preferred Reporting Items for Systematic Reviews and Meta-Analysis Protocols. The entire process will be carried out by peers, that is, by the work of

two independent reviewers, with any discrepancies resolved by a third reviewer. If the results allow, meta-analytic statistical methods will be applied, including meta-analysis or meta-regression, sensitivity, consistency, precision, and certainty of evidence analyses.

Discussion: This systematic review is expected to deepen understanding of COVID-19's impact on professors' health, with a focus on analyzing executive functions. Additionally, it will allow for the development of strategies aimed at implementing protective measures and mitigating post-infection effects.

Key-words: Executive functions; COVID-19; systematic review; public health; educators.

Introduction

The coronavirus disease (COVID-19) has led to significant challenges in the world, affecting various aspects of daily life, including education (1). It can trigger neuroinflammation, commonly known as a "cytokine storm" (2), hypoxia (3), vascular damage (4), and feelings of tiredness and stress (5), potentially leading to neuronal damage and cognitive deficits (6). Indeed, previous research has linked such impairments to a decline in executive functions (EFs) (7-9). These functions, also known as cognitive control, are necessary for actions requiring concentration and attention (10, 11).

The literature has identified three central EFs (12): inhibitory control (i.e., managing inappropriate behaviors (10, 11) and maintaining selective attention (12, 13)), working memory (i.e., retention and manipulation of information) (14), and cognitive flexibility (i.e., adaptation to new rules (11) and perspectives (10)). From these core functions, more complex skills such as planning, decision-making, problem-solving, and reasoning emerge (11). These skills are important for teaching activities as they allow the establishment of relationships between ideas, the integration of new information with existing knowledge, and the ability to remember sequences of events.

Understanding whether COVID-19 affects EFs is essential, especially considering its potential negative effects on educators. A report explained that COVID-19 led to impairments in executive function in 36% of cases (15). Other studies have shown similar results, indicating that COVID-19 infection may lead to disorders related to confusion and attention difficulties (15).

Despite initial studies suggesting a connection, gathering more solid evidence on whether COVID-19 affects educators' EFs is important. To the best of our knowledge, no systematic review has addressed this issue (16, 17). The guiding research question was the following: "Does COVID-19 affect teachers' EF in the long term?" Therefore, this

systematic review aims to explore the effects of COVID-19 on educators' EF. This knowledge can enhance our scientific understanding of the topic because EFs represent an essential element for teaching activities. Thus, this study could potentially contribute to proposals to improve educators' mental health after the pandemic, which could indirectly impact the quality of teaching for students.

Materials and Methods

Protocol and registration

This systematic review protocol was written based on the recommendations of the Preferred Reporting Items for Systematic Reviews and Meta-analyses for Protocols (PRISMA-P) (18). After defining the research problem, a preliminary search will be conducted in the databases to identify studies that form the basis of the research.

The study protocol was registered in the International Prospective Register of Systematic Reviews (PROSPERO) (CRD42024558019). If there is a need for modifications to ongoing research, these changes will be reported to PROSPERO.

Search strategy and databases

A systematic review will be conducted to gather bibliographic data from the following databases: Web of Science Core Collection, Scopus, MEDLINE/PubMed via the National Library of Medicine interface, Cochrane Library, Latin American and Caribbean Health Sciences, Embase, ERIC, and SPORTDiscus via the EBSCOhost interface. The databases will be searched until March 2025 to identify potential articles for inclusion in this systematic review. Additionally, the search strategy was complemented by screening the reference lists of the included studies.

This systematic review employed the following Medical Subject Headings terms as the primary search terms: "COVID-19" and "executive function". The Boolean

operator “OR” will be used to group the synonyms of each term. The terms will then be organized into blocks and connected by the Boolean operator "AND" to complete the search strategy. The search strategy will comprise three blocks that refer to the public (educators), exposure (COVID-19), and outcome (executive functions), as shown in Table 1.

Table 1 - Search strategy organized into blocks.

**Search**	**Blocks with search terms**
#1	(teacher OR teach* OR professor OR professors OR advisor OR advisors OR lecturer OR lecturers OR “mentor” OR mentors OR instructor OR instructors OR pedagogue OR pedagogues OR schoolteacher OR schoolteachers OR preceptor OR preceptors OR educator OR educators OR educationist OR educationists OR tutor OR tutors)
#2	(“COVID-19” OR “COVID19” OR “coronavirus” OR “sars” OR “2019-nCoV” OR “CoV-2” OR “long covid” OR “omicron” OR “delta” OR “severe acute respiratory syndrome”)
#3	(“executive function*” OR “executive control” OR cognition OR “cognitive function*” OR “cognitive performance” OR “cognitive inhibition” OR “cognitive flexibility” OR “working memory” OR “frontal lobe function*” OR “cognitive flexibility” OR “inhibitory control” OR “working memory” OR “cognitive skill*” OR “cognitive control” OR “mental function*”)
#4	(#1) AND (#2) AND (#3)

The titles, abstracts, and keywords in the databases will be searched. The metadata extracted from the databases adhered to the Preferred Reporting Items for Systematic Reviews and Meta-Analyses (PRISMA-S) search checklist. The results of the database searches will be entered into the first round of screening in the Rayyan software. Subsequently, the data from the selected studies will be extracted independently by each reviewer.

Eligibility criteria

The eligibility criteria for this study were determined based on population, exposure, comparison, and outcome (PECO). Accordingly, the following parameters are established: population (P), educators; exposure (E), COVID-19 infection; and outcome (O), executive function.

This systematic review will include articles published after the onset of the COVID-19 pandemic (March 11, 2020). To be considered eligible, articles have to meet the following inclusion criteria: i) original published articles and peer-reviewed studies, ii) studies with educators, and iii) studies that analyzed executive functions. Conversely, the exclusion criteria were as follows: i) duplicates (studies published in more than one journal were carefully reviewed to confirm elimination) (19), ii) literature reviews, and iii) studies with retractions. Retraction was identified using the Scite tool (20).

Reviewer training

Before beginning the screening process, researchers involved in eligibility assessments will undergo training focused on the study eligibility criteria (21-23). The researchers will first receive specific instructions on how to use the Rayyan software. Following which they will assess the eligibility of 50 references (i.e., abstracts and titles) to standardize the screening process. Additionally, a specific training session will be held to standardize the assessment of the risk of bias.

Data extraction, synthesis, and analysis

After extracting the data from the databases, it will be added to the Rayyan online software. This tool allows the peer review process to be conducted by independent reviewers who begin by reading the titles and abstracts to apply the eligibility criteria. Disagreements will be resolved by consensus with a third researcher. The selection process is illustrated in the flowchart (Fig. 1) (24).

A spreadsheet will be created to include all fields presented in Table 2 for data extraction. Two independent reviewers will evaluate and extract the data. Any discrepancies will be addressed by consensus with a third reviewer. Descriptive and content analyses of the results will also be conducted based on the impact of COVID-19 on educators' executive functions.

Table 2 Data extraction from the included studies.

**Author, year, and country of publication**	**Study design**	**Intervention used**	**Sample**	**Instrument used**	**Statistical analysis**	**Main results**

The WebPlotDigitizer will be used to collect the data available in the graphs. If any data are missing from the primary studies, the authors will be contacted to obtain them. After collecting all the data, the percentage of agreement and Cohen's kappa will be calculated to evaluate the accuracy of each stage of the data extraction process. Cohen's kappa coefficient will be interpreted as a measure of agreement between evaluators, ranging from 0 to 1, where values closer to 1 indicate high agreement beyond chance, and values closer to 0 indicate low or no agreement (25, 26).

The risk of bias will be evaluated using the 27-item Downs and Black scale (27). Notably, this scale is acceptable for quantitative studies. A quality score will be calculated for each study; the obtained score will be expressed as a percentage relative to the maximum possible score for the study design (28). In summary, studies with scores equal

to or above 70% will be considered to have a low risk of bias. The risk of bias will be evaluated by two independent reviewers. In case of disagreement, a third reviewer will be consulted.

Data transformation and results prioritization

A data adjustment process will be performed to integrate and simplify the interpretation of results obtained from the systematic review. The function will be prioritized for the evaluation. If more than one tool will be used to measure a variable, the one with the greatest reliability and validity will be selected.

Main meta-analyses and/or meta-regressions

All main analyses will consider studies that have investigated the impact of COVID-19 on the executive functions of the teaching public. Therefore, the main outcomes focused on in our analysis are EFs. Importantly, these analyses will be categorized according to the type of study, separating cross-sectional studies from cohort studies if the search returns to both models. This organization ensures that the analyses are adapted to the context and peculiarities of each study design.

The pooled effect sizes will be calculated together with their 95% confidence intervals. In this way, the analyses will make it possible to assess the effect of COVID-19 on educators' EFs. Based on the results obtained in the systematic review, subgroup meta-analyses will be performed to investigate the variability and possible modifications in the effects between different profiles of individuals within the sample. Variables such as age, sex, socioeconomic context, and network of activities should be considered.

Sensitivity analysis

We will conduct a sensitivity analysis to assess the reliability of the data. This involves systematically removing one study at a time from the meta-analysis to determine

whether the results are significantly affected. This is crucial for identifying the maximum and minimum effect sizes. Additionally, another sensitivity analysis will involve excluding studies with a "high risk of bias" (29). Additional sensitivity analyses will be conducted.

Consistency analysis and precision analysis

Each analyzed dataset will be evaluated for consistency. Thus, we will use the *Q* statistic to assess consistency, with $P \leq 0.05$ indicating the presence of significant heterogeneity. Additionally, the I^2 statistic will be employed to detect any heterogeneity between effect estimates, with 0 to 25% indicating "low heterogeneity," 25 to 50% indicating "moderate heterogeneity," and more than 50% indicating "high heterogeneity" (30). Finally, to assess the precision of each meta-analysis, the power of all analyses will be calculated (31).

Publication bias

The analysis included a check for publication bias across all meta-analyzed datasets (32). We will begin by generating funnel plots that display the effect sizes of individual studies on the x-axis against the study size or precision on the y-axis (33). Visual asymmetry in these plots would suggest publication bias (33).

Moreover, we will perform a "trim and fill" analysis in search of missing studies, which should be illustrated in the funnel plot. (34–36). This method identifies potentially missing studies using funnel plots and provides an adjusted meta-analysis (34). Finally, Egger's test will be used to ensure greater objectivity in detecting publication bias (35).

Certainty of evidence

The GRADE (Grading of Recommendations, Assessment, Development, and Evaluations) Working Group method will be used to assess the certainty of the evidence.

To evaluate the risk of bias, the certainty of evidence will be downgraded by two levels if 50–75% of the studies are rated as having a "low risk of bias" and by one level if less than 50% of the studies are considered to have a "low risk of bias.” If the power of detection of the analysis is less than 90%, the certainty will be reduced by one level, and if the power is less than 80%, it will be reduced by two levels.

The certainty of the evidence will be downgraded by one level for "moderate heterogeneity" and by two levels for "high heterogeneity.” The presence of publication bias, as indicated by the Egger test result ($P < 0.05$), lowers certainty by one level. Additionally, when indirect evidence is involved, such as by extrapolating findings to populations not specified in the study, the certainty is downgraded by one level. Despite these downgrading criteria, the certainty of the evidence may increase if there is a "large" or "very large" effect size (31). This method classifies each meta-analysis result into one of four levels: high (⊕⊕⊕⊕), moderate (⊕⊕⊕), low (⊕⊕), or very low (⊕).

Discussion

This systematic review aims to understand the impact of the COVID-19 pandemic on educators’ EFs. The COVID-19 pandemic has had short-, medium-, and long-term consequences, many of which remain unknown (16). By systematically examining these effects, this review provides critical insights that can inform the development of interventions to support educators’ mental health and enhance the quality of student teaching in post-pandemic times.

EFs are fundamental to an educator’s professional performance and play a central role in the execution of a series of recurring tasks in this type of work activity (36). Therefore, understanding the impact of COVID-19 on these variables is highly valuable for a class of workers who perform socially and economically relevant roles. Furthermore,

understanding the long-term impact of the virus on the EFs of education professionals will provide the necessary support for creating intervention plans aimed at minimizing consequences, as well as for preventing new COVID-19 infections and new pandemic episodes.

This systematic review protocol has several strengths that could contribute to improving educators' activities, health, and quality of life. This field is still poorly explored and requires the attention of the scientific community. Gathering these data will allow the planning of public-private interventions aimed at mitigating the consequences of COVID-19 on the professional activity of educators, which certainly has an impact on the quality of young students' education.

Using an appropriate and robust methodology guarantees data validity and reliability. For this purpose, PRISMA-P (18) and PRISMA (24) will be used as reference methods with the PECO strategy for organizing and managing database searches. Furthermore, all reviewer training guarantees fairness, assertiveness, and efficiency in the peer review process.

A potential limitation of this protocol is the possibility that no studies would be returned after using the database search strategies, leading to the establishment of an "empty review." However, this still represents an important result, suggesting a knowledge gap in the literature that indicates the need for direct efforts to explore this research problem. Another limitation that may arise is the great variability in the characteristics of the population used as a sample, leading to heterogeneity, which makes the synthesis and analysis of results more complex. One proposal to alleviate this issue is to classify the participants into subgroups to perform statistical analyses.

Conclusion

This study aims to identify and understand the impact of a serious global event, the COVID-19 pandemic, on educators' EFs. The findings are expected to contribute to the development of interventions aimed at caring for educators' mental health and quality of life, which will certainly impact the quality of teaching activities and student training.

Acknowledgments

We would like to thank Fundação de Amparo à Pesquisa do Estado de Goiás (FAPEG), the Conselho Nacional de Desenvolvimento Científico e Tecnológico (CNPq), the Instituto Federal Goiano-Campus Ceres and the Grupo de Estudos Sobre a Saúde da Criança e do Adolescente (GPSaCA) for their support.

Ethics Statement:

N/A.

References

1. Lambert H, Gupte J, Fletcher H, Hammond L, Lowe N, Pelling M, et al. COVID-19 as a global challenge: towards an inclusive and sustainable future. Lancet Planet Health. 2020 Aug;4(8):e312-e4.
2. Kempuraj D, Selvakumar GP, Ahmed ME, Raikwar SP, Thangavel R, Khan A, et al. COVID-19, Mast Cells, Cytokine Storm, Psychological Stress, and Neuroinflammation. Neuroscientist. 2020 Oct-Dec;26(5-6):402-14.
3. Jahani M, Dokaneheifard S, Mansouri K. Hypoxia: A key feature of COVID-19 launching activation of HIF-1 and cytokine storm. J Inflamm (Lond). 2020;17:33.
4. Amraei R, Rahimi N. COVID-19, Renin-Angiotensin System and Endothelial Dysfunction. Cells. 2020;9(9).
5. Rocona PC, Parob FM, Vieirac RD, Andradea ACS, Barbalho-Moulimb MC, Oliveirad CGT, et al. Self-perceived stress by women during the COVID-19 pandemic: an online survey with Brazilian physiotherapists. Journal of Human Growth and Development. 2024;34(1):22-30.
6. Nersesjan V, Fonsmark L, Christensen RHB, Amiri M, Merie C, Lebech AM, et al. Neuropsychiatric and Cognitive Outcomes in Patients 6 Months After COVID-19 Requiring Hospitalization Compared With Matched Control Patients Hospitalized for Non-COVID-19 Illness. JAMA Psychiatry. 2022 May 1;79(5):486-97.
7. Teoh NSN, Gyanwali B, Lai MKP, Chai YL, Chong JR, Chong EJY, et al. Association of Interleukin-6 and Interleukin-8 with Cognitive Decline in an Asian Memory Clinic Population. J Alzheimers Dis. 2023;92(2):445-55.
8. Chroboczek M, Kostrzewa M, Micielska K, Grzywacz T, Laskowski R. Effect of Acute Normobaric Hypoxia Exposure on Executive Functions among Young Physically Active Males. J Clin Med. 2021 Apr 8;10(8).

9. Hoagey DA, Lazarus LTT, Rodrigue KM, Kennedy KM. The effect of vascular health factors on white matter microstructure mediates age-related differences in executive function performance. Cortex. 2021 Aug;141:403-20.
10. Diamond A. Executive Functions. . Annual Review of Psychology. 2013;64:135-68.
11. Blair C. Educating executive function. Wiley Interdiscip Rev Cogn Sci. 2017 Jan;8(1-2).
12. Hofmann W, Schmeichel BJ, Baddeley AD. Executive functions and self-regulation. Trends Cogn Sci. 2012 Mar;16(3):174-80.
13. Ardila A. On the evolutionary origins of executive functions. . Brain and cognition 2008;68:92-9.
14. Baddeley A. The episodic buffer: A new component of working memory? Trends in Cognitive Sciences. 2000;4:417-23.
15. Helms J, Kremer S, Merdji H, Clere-Jehl R, Schenck M, Kummerlen C, et al. Neurologic Features in Severe SARS-CoV-2 Infection. N Engl J Med. 2020 Jun 4;382(23):2268-70.
16. Wu Y, Xu X, Yang L, Liu C, Yang C. Nervous system damage after COVID-19 infection: Presence or absence? Brain Behav Immun. 2020 Jul;87:55.
17. Mao L, Wang M, Chen S, He Q, Chang J, C. H. Neurological manifestations of hospitalized patients with COVID-19 in Wuhan, China: a retrospective case series study. . MedRxiv. 2020.
18. Shamseer L, Moher D, Clarke M, Ghersi D, Liberati A, Petticrew M, et al. Preferred reporting items for systematic review and meta-analysis protocols (PRISMA-P) 2015: elaboration and explanation. BMJ. 2015 Jan 2;350:g7647.
19. McKeown S, Mir ZM. Considerations for conducting systematic reviews: evaluating the performance of different methods for de-duplicating references. Syst Rev. 2021 Jan 23;10(1):38.
20. Perez-Neri I, Pineda C, Flores-Guerrero JL, Estevao MD, Vasanthan LT, Lorente S, et al. Adherence to literature search reporting guidelines in leading rheumatology journals' systematic reviews: umbrella review protocol. Rheumatol Int. 2022 Dec;42(12):2135-40.
21. Fernandes MDV, Mendonca CR, da Silva TMV, Noll PRES, de Abreu LC, Noll M. Relationship between depression and quality of life among students: a systematic review and meta-analysis. Sci Rep-Uk. 2023 Apr 25;13(1).
22. Noll M, Wedderkopp N, Mendonca CR, Kjaer P. Motor performance and back pain in children and adolescents: a systematic review and meta-analysis protocol. Syst Rev. 2020 Sep 14;9(1):212.
23. Moreira MVB, Visoto TF, Noll PRS, Drezet J. What do we know about the perpetrators of sexual homicide of adult victims? A systematic review and qualitative meta-synthesis. Journal of Human Growth and Development. 2024.;34(1):141-65.
24. Page MJ, McKenzie JE, Bossuyt PM, Boutron I, Hoffmann TC, Mulrow CD, et al. The PRISMA 2020 statement: an updated guideline for reporting systematic reviews. Rev Esp Cardiol (Engl Ed). 2021 Sep;74(9):790-9.
25. McHugh ML. Interrater reliability: the kappa statistic. Biochem Med (Zagreb). 2012;22(3):276-82.
26. Costa WPD, Fernandes M, Memon AR, Noll P, Sousa MM, Noll M. Factors influencing the work of researchers in Scientific Initiation: A systematic review protocol. PLoS One. 2024;19(1):e0297186.
27. Dixon-Woods M, Bonas S, Booth A, Jones DR, Miller T, Sutton AJ, et al. How can systematic reviews incorporate qualitative research? A critical perspective. Qualitative Research. 2006;6:27-44.
28. Noll M, de Mendonca CR, de Souza Rosa LP, Silveira EA. Determinants of eating patterns and nutrient intake among adolescent athletes: a systematic review. Nutr J. 2017 Jul 28;16(1):46.
29. SILVA KVC, COSTA BD, GOMES A, C.;, SAUNDERS B, MOTA JF. Factors that Moderate the Effect of Nitrate Ingestion on Exercise Performance in Adults: A Systematic Review with Meta-Analyses and Meta-Regressions. Advances in Nutrition. 2022;13(5):1866-81.
30. Higgins JP, Thompson SG, Deeks JJ, Altman DG. Measuring inconsistency in meta-analyses. BMJ. 2003 Sep 6;327(7414):557-60.

31. Guyatt GH, Oxman AD, Vist GE, Kunz R, Falck-Ytter Y, Alonso-Coello P, et al. GRADE: an emerging consensus on rating quality of evidence and strength of recommendations. BMJ. 2008 Apr 26;336(7650):924-6.
32. Winters M, Weir A. Grey matters; on the importance of publication bias in systematic reviews. Br J Sports Med. 2017 Mar;51(6):488-9.
33. Egger M, Smith GD. Misleading meta-analysis. BMJ. 1995 Mar 25;310(6982):752-4.
34. Duval S, Tweedie R. Trim and fill: A simple funnel-plot-based method of testing and adjusting for publication bias in meta-analysis. Biometrics. 2000 Jun;56(2):455-63.
35. Egger M, Davey Smith G, Schneider M, Minder C. Bias in meta-analysis detected by a simple, graphical test. BMJ. 1997 Sep 13;315(7109):629-34.
36. Sankalaite S, Huizinga M, Dewandeleer J, Xu C, de Vries N, Hens E, et al. Strengthening Executive Function and Self-Regulation Through Teacher-Student Interaction in Preschool and Primary School Children: A Systematic Review. Front Psychol. 2021;12:718262.

 Click here to access/download;Figure;fig 1.jpg

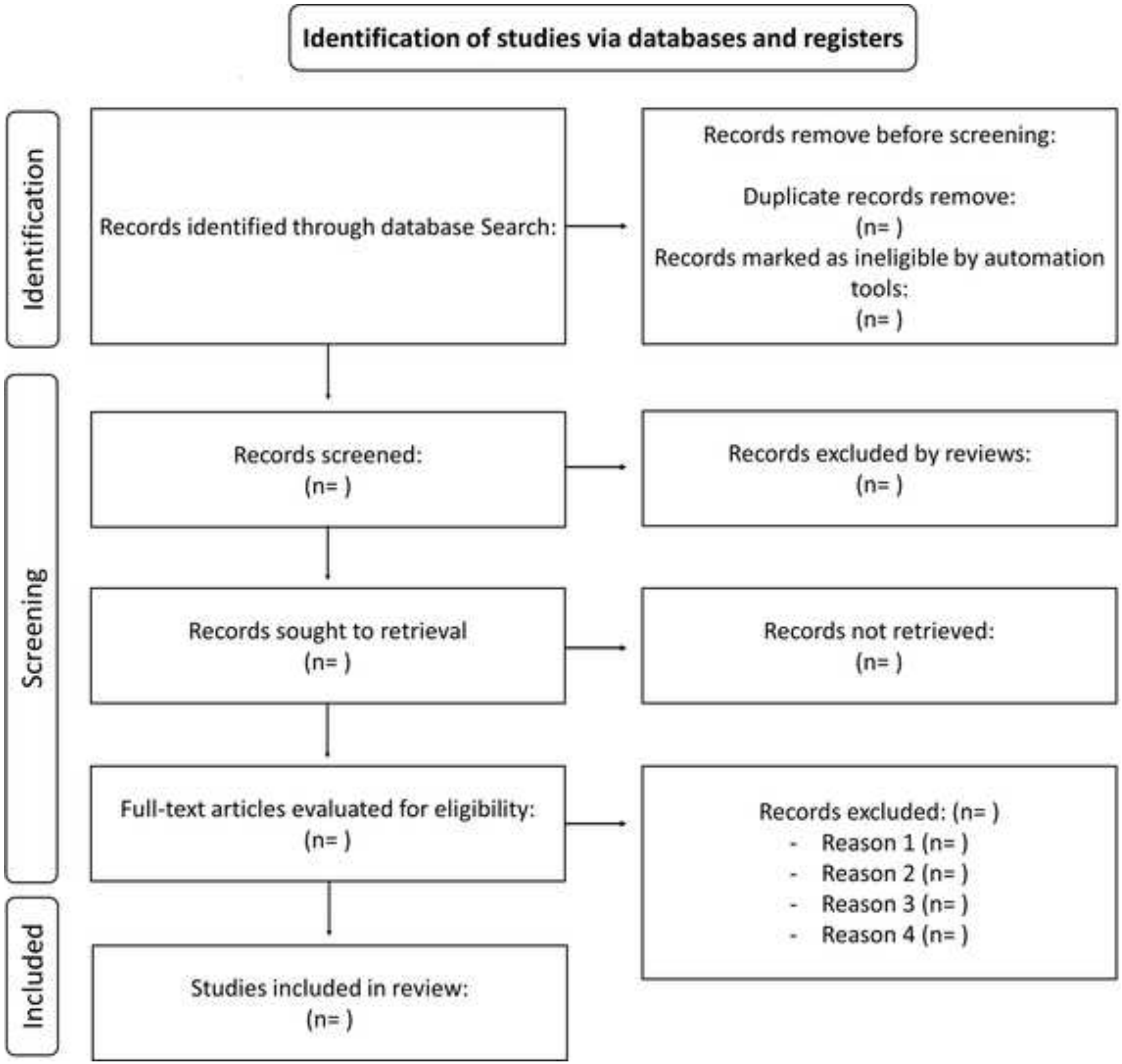

Supplement: Supplementary file 1 [file pone.0350156.s001.pdf]
