# Supplementary material for: Consequences of COVID-19 infection on educators’ executive functions: A systematic review and meta-analysis protocol
Source: PLoS One. 2026 Oct 14;21(10):e0350156. doi: 10.1371/journal.pone.0350156 (PMC13667799; doi:10.1371/journal.pone.0350156)
Supplement: Supplementary file 2 [file pone.0350156.s004.pdf]

Dear reviewers, we are very grateful for your careful analysis and valuable comments. We use all of them to improve the methodological quality and scientific rigor of our work. Please see the answers to the questions below. The revised sentences are in red color in the manuscript file.

Reviewer #1:

Thank you for submitting your manuscript for review. After a thorough evaluation, I believe that the article requires substantial improvements before it can be reconsidered for publication.

In particular, the introduction needs significant development. This section should not only present the topic but also provide a clear context, demonstrate its relevance within the field, and justify the study's purpose. As it stands, the rationale appears underdeveloped, which gives the impression that the topic is being addressed arbitrarily—this can introduce bias into the overall approach of the work.

Authors' Response: We appreciate the insightful comment. In response, we have significantly revised the introduction to provide a clearer and more robust context for the study. The updated section now includes: (1) a more comprehensive background on the cognitive consequences of COVID-19 infection, (2) a detailed explanation of executive functions and their importance in academic professions, and; (3) a clearer justification for focusing specifically on professors as a population of interest. Please see highlighted in the introduction section.

Additionally, the methodology section should be rewritten to ensure greater transparency. The procedures must be described in enough detail to allow for replication by other researchers. In this regard, I also recommend improving the eligibility criteria, which are currently too vague and insufficiently specific to support reproducibility.

Authors' Response: We appreciate the reviewer's thoughtful feedback regarding the clarity and transparency of our methodology. In response, we have thoroughly revised the Methods section to ensure it provides sufficient detail to allow for replication by other researchers. Please, See highlighted in the text, as follows:

Table 2. Eligibility criteria.

	Inclusion	Exclusion
Population	Adult individuals (>18 years) who work as a teacher/educator at any educational levels	individuals diagnosed with psychiatric illnesses
Exposure	COVID-19 infection	- Not exposed to COVID-19 infection
Outcomes	Analysis of the executive functions of teachers who were infected with COVID-19	Studies that used non-validated methods to analyze executive functions<br><br>Self-reported executive functioning<br><br>Studies that analyzed executive functions outside the context of COVID-19
Study design	Cross-sectional, longitudinal; random and non-random studies	Literature reviews; studies with retractions

"

You will find detailed comments in the attached document.

Authors' Response: We appreciate the careful review. All points have been considered. The point-by-point response follows.

Abstract

as I understand it is a protocol not a review so the focus should be on the protocol and not the review itself.

Authors' Response: Adjusted. Please see abstract, as follows:

"This systematic review protocol aims to identify, synthesize, and critically evaluate the existing body of literature on the effects of COVID-19 on educator's executive functions."

The elegibility criteria should be reported. Please review https://www.prisma-statement.org/abstracts

Authors' Response: Adjusted. Please see abstract, as follows:

"**Method:** We will search seven databases—Web of Science Core Collection, Scopus, MEDLINE/PubMed via the National Library of Medicine interface, Cochrane Library,

Latin American and Caribbean Health Sciences, Embase, ERIC, and SPORTDiscus via the EBSCOhost interface. Data extraction and analysis will be performed according to the guidelines of the Preferred Reporting Items for Systematic Reviews and Meta-Analysis Protocols. The entire process will be carried out for two independent reviewers, with any discrepancies resolved by a third reviewer. The eligibility criteria followed the PECO strategy. To be included, articles will have to be: i) original published, ii) studies with educators, and iii) studies that analyzed executive functions. Conversely, exclusion criteria will be: i) duplicates, ii) literature reviews, and iii) studies with retractions. If the search strategy yields a sufficient number of methodologically comparable studies, meta-analytic statistical methods will be applied, including meta-analysis or meta-regression, sensitivity, consistency, precision, and certainty of evidence analyses."

I suggest not using the same words as in the title to generate more impact.

Authors' Response: Adjusted, as follows:

"**Key-words:** Executive control; SARS-COV-2; public health; educators."

Introduction:

1) I think that, since it is a protocol, the introduction should be much more in-depth than it is at present. It is important that they not only discuss each concept but also explain some studies and delve into existing results that demonstrate the relevance of studying the problem. If this is not done, it may seem that the topic is chosen at random, which may generate biases.

2) Explaining this can be good if we talk about the effects of people who have suffered from covid, but it is not so interesting for the question they have asked, I think you should check well what they are looking to realize. Your theory is that all these effects affected executive functions but they do not discuss other variables that also influence and seem to reduce the problem in a very simple way. I recommend that they also discuss other related biopsychosocial aspects.

3) I think this definition is quite simple for what the executive functions entail, I suggest to go deeper into it.

4) since executive functions have different authors and different definitions because of this, I suggest commenting on this as well or, at least, pointing out why it is decided to follow this line itself. I think it is necessary because the way it is written it seems that there is an absolute consensus.

5) this should be deepened

6) In order to be able to say this, they must also explain what already exists. Are there any other studies on similar topics?

7) I think this did not go into too much depth to be able to take this on.

Authors' Response: We appreciate the insightful comments. we have taken your suggestion and significantly modified the introduction to improve the text, as follows:

"The coronavirus disease (COVID-19) has led to significant challenges in the world, affecting various aspects of daily life, including education (1). While much attention has been given to the respiratory and systemic effects of the virus, increasing evidence suggests that COVID-19 also affects the central nervous system (CNS) (2). There are also reports of psychosocial issues, leading to disorders such as anxiety and depression (3, 4). Therefore, we are aware that the extent of the virus infection and the pandemic context experience have broad repercussions. This suggests the need for greater in-depth analysis of the long-term consequences for the population.

Aiming to broaden understanding, a biopsychosocial approach is necessary. From a biological perspective, COVID-19 has the potential to lead to neuroinflammation—commonly referred to as a "cytokine storm" (5), hypoxia (6), vascular damage (7) and, potentially leading to neuronal damage and cognitive deficits (8). The psychological aspect is also affected. Studies point to the incidence of feelings of tiredness and stress (9, 10), the "brain fog" (11) and mental confusion (12). The social consequences have also been devastating. The pandemic has forced a routine break, distancing and reclusion, and work dynamics changes, generating problems such as anxiety and family financial crises, for example (13). Indeed, previous research has linked such impairments to a decline in executive functions (EFs) (14-16). These effects can even contribute to neuronal injury and cognitive deficits (17, 18).

The impact on the CNS occurs as SARS-CoV-2 is a neurotrophic virus, that is, has attraction for nerve cells (19). The virus reaches the CNS through sensory and motor pathways, for example, the olfactory nerve pathway. When it reaches the CNS, it triggers demyelination of nerve fibers and inflammatory processes (19). These trigger a series of neuropsychiatric disorders. In the short-term, complications such as cerebrovascular

events, headache, encephalopathies, anosmia, among others, are observed. (20-23). The literature has already become aware of the long-term infection consequences. Symptoms of inattention and dysexecutive dysfunction are common (24). There are also reports of frontotemporal hypoperfusion, as well as structural brain abnormalities, mainly in the thalamic and temporal regions (25). Demyelination also causes an information processing reduction, directly affecting performance in tasks related to the use of central executive functions (2).

Among the cognitive domains potentially affected, executive functions (EFs) have emerged as a particular concern (7–9). These high-order cognitive processes, also known as cognitive control, include abilities essential for regulating thoughts and behaviors, such as maintaining attention, organizing tasks, problem-solving, and adapting to new information. The literature has identified three central EFs (26): inhibitory control (i.e., managing inappropriate behaviors (17, 18) and maintaining selective attention (26, 27)), working memory (i.e., retention and manipulation of information) (28), and cognitive flexibility (i.e., adaptation to new rules (18) and perspectives (10). From these core functions, more complex skills such as planning, decision-making, problem-solving, and reasoning emerge (18). For the purposes of this review, executive functions will be operationally defined as cognitive processes assessed through validated instruments or structured tasks that explicitly measure at least one of the following domains: inhibitory control, working memory, or cognitive flexibility (29-31). These skills are important for teaching activities as they allow the establishment of relationships between ideas, the integration of new information with existing knowledge, and the ability to remember sequences of events.

For educators, these EFs are critical to the performance of professional duties. Teaching demands the integration of information, multitasking, responsiveness to

dynamic classroom environments (including online formats), and the ability to make pedagogical decisions under time constraints. Cognitive impairments in these areas may, therefore, directly compromise educational quality and teacher well-being. A report explained that COVID-19 led to impairments in executive function in 36% of cases (32). Other studies have shown similar results, indicating that COVID-19 infection may lead to disorders related to headache, confusion and attention difficulties (21, 23, 24, 32).

In addition to fear, COVID-19 has created unexpected and high work demands. This overloading teachers and imposing a routine that has resulted in a poor well-being (33). Studies show that this disruption in well-being affects cognitive abilities, resulting in low scores in EFs analyses (33, 34).

Despite initial studies suggesting a connection, gathering more solid evidence on whether COVID-19 affects educators' EFs is important. To the best of our knowledge, no systematic review has addressed this issue (35, 36). The guiding research question was the following: "Does COVID-19 affect teachers' EFs in the long term?" Therefore, this systematic review aims to explore the effects of COVID-19 on educators' EFs. This knowledge can enhance our scientific understanding of the topic because EFs represent an essential element for teaching activities. Thus, this study could potentially contribute to proposals to improve educators' mental health after the pandemic, which could indirectly impact the quality of teaching for students."

Materials and Methods

Any type of teacher? The psychosocial variables between levels are very different, as well as their tasks. Review the entire introduction to give context to this

Authors' Response: Yes. This study was designed to cover all educator's, from different levels and subjects. We believe it is necessary, since we know little about the subject. Later, specifications may be made. Logically, depending on the results obtained from the databases, these groupings and analyses may be carried out.

Hasn't this preliminary search already been carried out?

Authors' Response: Yes. However, few studies have been found.

update the date as it is a protocol, I suggest that you extend it to the publication of the protocol.

Authors' Response: Adjusted. Please see in the text, as follows:

"The databases will be searched by August 2025 to identify potential articles for inclusion in this systematic review."

I am fine with the format in which you show it but I suggest you indicate each specific search strategy as this allows for replicability, I suggest you review the table at https://doi.org/10.1371/journal.pone.0296273.

Authors' Response: Dear reviewer, we are grateful for the suggestion and have decided to incorporate this table into our work (see Table 2). However, we believe that we should keep Table 1, since it shows us the search strategy used.

why was * not used as in the previous case?

Authors' Response: The * is a truncation operator that allows you to recover variations of the same word root. In this case, we believe that we have exhausted the possibilities and it would not be necessary to use it.

it is repeated

Authors' Response: It was removed from the table.

suggest pointing out in the text an example of the search number you found in this preliminary adjustment

Authors' Response: Dear author, we have chosen not to include this information. We believe that it may seem that we are skipping steps and breaking the rules of the PRISMA manual. We are working to soon publish these results in a systematic review format.

insert link after

Authors' Response: Inserted. Please see text, as follows:

"Search results will be exported from the databases in RIS format, stored and organized in Mendeley (reference manager), and subsequently imported into Rayyan software (https://new.rayyan.ai/) for screening and selection."

but in what way? In parallel, blinded...?

Authors' Response: Inserted. Please see text

, as follows:

"The reviewers will carry out the analyses in parallel and using the "blind" feature."

remember that this is the protocol, so you must detail everything very specifically and go very well into each section of the methodology. This section should be improved.

Authors' Response: The text has been adjusted. Please let me know what you think.

Reference

Authors' Response: Reference inserted. See text, pleas

, as follows:

"The eligibility criteria for this study were determined based on population, exposure, comparison, and outcome (PECO) (Tab. 2) (38, 39).

But in what way? just having gone through it or having been infected?

Authors' Response: Adjusted. Please see in the text

, as follows:

"Accordingly, the following parameters are established: population (P), educators; exposure (E), have been infected with COVID-19; and outcome (O), executive function."

it is necessary to specify this very well, what type of teacher and what characteristics should be excluded. In addition, they should define the characteristics very well because I think they could be biased, not only for being a teacher influences the covid in one way but also the age for example could also influence.

Authors' Response: Our goal is not to deal specifically with areas or levels of education, but rather to explore teaching activities in general. We believe that this broad mapping and data collection is important. Later, we will consider narrowing the analyses.

either way?

Authors' Response: No restrictions. We believe that we will find few studies, due to the specificity of the sample. Therefore, we will analyze any type of work that has focused on the executive functions of teachers.

It is very important not only to detail which one is not, but also which one is. Not all studies are going to give us the same indications nor are they going to be equally useful in the subject, this should be reviewed. I suggest that you use a better inclusion and exclusion table and go deeper into each section.

Authors' Response: Dear reviewer, the table has been inserted. Please see text, as follows:

Table 2. Eligibility criteria.

	Inclusion	Exclusion
Population	Adult individuals (>18 years) who work as a teacher/educator at any educational levels	- Children and adolescents.<br>- Non-teaching adults.
Exposure	COVID-19 infection	- Not exposed to COVID-19 infection
Outcomes	Analysis of the executive functions of teachers who were infected with COVID-19	Studies that used non-validated methods to analyze executive functions<br><br>Self-reported executive functioning<br><br>Studies that analyzed executive functions outside the context of COVID-19
Study design	Cross-sectional, longitudinal; random and non-random studies	Literature reviews; studies with retractions

"

should explain the exact data and indices to be used

Authors' Response: Adjusted. Please see in the text, as follows:

"Descriptive and content analyses of the results will also be conducted based on the impact of COVID-19 on educators' executive functions. The extracted data will include quantitative indicators such as means, standard deviations, and effect sizes related to executive function performance (e.g., working memory, cognitive flexibility, inhibitory control), as well as qualitative themes derived from narrative reports or interviews.

Indices such as test scores, neuropsychological assessments, and self-reported measures will be used to categorize and synthesize findings."

reference

Authors' Response: Inserted. Please see text, as follows:

"The WebPlotDigitizer will be used to collect the data available in the graphs (47)."

how many times for how long?

Authors' Response: Adjusted. Please see in the text, as follows:

"If any data are missing from the primary studies, the authors will be contacted by email up to three times over a period of four weeks. If no response is received after this period, the study will be included in the review with the available data, and the missing information will be acknowledged as a limitation."

You included several studies, they should take into account tools for each type of study.

Authors' Response: Adjusted. Please see in the text , as follows:

"In summary, studies with scores equal to or above 70% will be considered to have a low risk of bias. The risk of bias will be evaluated by two independent reviewers. In case of disagreement, a third reviewer will be consulted. Different validated tools can will be applied according to the design of each included study: for observational studies, the Joanna Briggs Institute (JBI) critical appraisal tools will be used; for randomized controlled trials, the Cochrane Risk of Bias Tool (RoB 2.0); for qualitative studies, the JBI Checklist for Qualitative Research; and for mixed-methods studies, the Mixed Methods Appraisal Tool (MMAT).

"

this was not mentioned in the title, it should be added.

Authors' Response: Adjusted. Please see title, as follows:

the software, the moderators and the indexes to be taken into account were not explained. It should be reviewed.

Authors' Response: Adjusted. Please see in the text, as follows:

All main analyses will consider studies that have investigated the impact of COVID-19 on the executive functions of the teaching public. Therefore, the main outcomes focused on in our analysis are EFs. Importantly, these analyses will be categorized according to the type of study, separating cross-sectional studies from cohort studies if the search returns to both models. This organization ensures that the analyses are adapted to the context and peculiarities of each study design.

The pooled effect sizes will be calculated together with their 95% confidence intervals, using the R statistical software. In this way, the analyses will make it possible to assess the effect of COVID-19 on educators' EFs. Based on the results obtained in the systematic review, subgroup meta-analyses will be performed to investigate the variability and possible modifications in the effects between different profiles of individuals within the sample. Variables such as age, sex, socioeconomic context, professional experience, and teaching level will be considered as potential moderators.

Statistical heterogeneity among the studies will be assessed using the I^2 statistic and the Chi-squared (Q) test. If applicable, publication bias will be examined through funnel plots and Egger's test.

with all the data?

Authors' Response: Adjusted. Please see in the text

, as follows:

"Additional sensitivity analyses will be conducted using all available data included in the meta-analysis, based on predefined eligibility criteria. These analyses will consider only complete datasets for the outcomes of interest (executive functions), ensuring consistency across comparisons. Sensitivity analyses will be performed after the initial meta-analysis and whenever significant heterogeneity or risk of bias is detected.

"

it is not explained with which software

Authors' Response: Inserted. Please see text , as follows:

"The GRADE assessments will be conducted using the GRADEpro GDT software (https://gradepro.org)."

it is a protocol, please review it

Authors' Response: Adjusted. Please see in the text , as follows:

"This systematic review protocol aims to understand".

it is not a review

Authors' Response: Adjusted. Please see in the text

, as follows:

"By systematically examining these effects, this protocol provides critical insights that can inform the development of interventions to support educators' mental health and enhance the quality of student teaching in post-pandemic times."

it is not the review, this is not the aim of the protocol

Authors' Response: Adjusted. Please see in the text

, as follows:

"This protocol aims to establish the appropriate methodological criteria for later carrying out the systematic review and meta-analysis to understand the impact of a serious

global event, the COVID-19 pandemic, on educators' EFs. The findings are expected to contribute to the development of interventions aimed at promoting educators' cognitive health, particularly executive functions, which are closely related to planning, attention, emotional regulation, and decision-making. By supporting these capacities, such interventions may indirectly favor improvements in educators' mental health and professional well-being, potentially influencing the overall quality of teaching and the educational environment.

"

You are talking about executive functions, do you think this will be the case? I think this is a statement that is too far removed from direct impact

Authors' Response: Adjusted. Please see in the text

, as follows:

"The findings are expected to contribute to the development of interventions aimed at promoting educators' cognitive health, particularly executive functions, which are closely related to planning, attention, emotional regulation, and decision-making. By supporting these capacities, such interventions may indirectly favor improvements in educators' mental health and professional well-being, potentially influencing the overall quality of teaching and the educational environment."

Reviewer #2:

1. A clearer operational definition of executive functions (e.g., which subdomains or assessments will be included).

Authors' Response: Inserted. Please see the introduction text, as follows:

"Among the cognitive domains potentially affected, executive functions (EFs) have emerged as a particular concern (7–9). These high-order cognitive processes, also known as cognitive control, include abilities essential for regulating thoughts and behaviors, such as maintaining attention, organizing tasks, problem-solving, and adapting to new

information. The literature has identified three central EFs (26): inhibitory control (i.e., managing inappropriate behaviors (17, 18) and maintaining selective attention (26, 27)), working memory (i.e., retention and manipulation of information) (28), and cognitive flexibility (i.e., adaptation to new rules (18) and perspectives (10). From these core functions, more complex skills such as planning, decision-making, problem-solving, and reasoning emerge (18). For the purposes of this review, executive functions will be operationally defined as cognitive processes assessed through validated instruments or structured tasks that explicitly measure at least one of the following domains: inhibitory control, working memory, or cognitive flexibility (29-31)."

2. Possibly adding a note about the scope limitation (i.e., generalizability to populations beyond professors) and whether any comparator analysis is anticipated in the future.

<u>Authors' Response</u>: Inserted. Please see the discussion text (lines: 322-325), as follows:

"Therefore, understanding the impact of COVID-19 on these variables is highly valuable for a class of workers who perform socially and economically relevant roles. Furthermore, understanding the long-term impact of the virus on the EFs of education professionals will provide the necessary support for creating intervention plans aimed at minimizing consequences, as well as for preventing new COVID-19 infections and new pandemic episodes."

"It is worth noting that our findings will be limited to educators and cannot be generalized to all populations. Subsequent studies should observe specific classes of educators and other populations."

Reviewer #3: Dear author,

I read your protocol submitted to PLOSone titled: "Consequences of COVID-19 infection on professors' executive functions: a systematic review protocol"
Below are my comments

General:
There is a large body of evidence supporting the notion of COVId-19 induced executive dysfunction, and there are many systematic reviews an meta-analyses published on the subject
You have identified a gap in the knowledge specifically around EFs in educators in the academic setup

<u>Authors' Response</u>: Dear reviewer, thank you for your insightful comment. Indeed, while there is a substantial body of evidence on COVID-19-induced executive dysfunction in general populations, our systematic review protocol specifically targets the impact on executive functions in educators within the academic setting. This focus is important because executive functions are critical for teaching activities, and understanding their potential impairment in this group may have important implications for educational processes and outcomes. Our goal is to

contribute knowledge that can inform targeted interventions to support educators' cognitive health and, consequently, the quality of education.

You stated that the subjects of interest in the review are professors. It would be helpful to change the word or provide an explanation early in the text. A suggestion owould be to use the term senior educator or experienced educator. Are you specifically after those with Professor designation? Any limitation on academic background or speciality? One can assume that a Professor in Math might be affected differently than a professor in English literature for example.

Authors' Response: This study was designed to cover all educators and teachers (we modified the text), from different levels and subjects. We believe it is necessary, since we know little about the subject. Later, specifications may be made. Logically, depending on the results obtained from the databases, these groupings and analyses may be carried out.

Line 191: " Additional sensitivity analyses will be conducted." - Specify how and when

Authors' Response: Inserted. Please see the text (lines: 248-253), as follows:

"Additional sensitivity analyses will be conducted using all available data included in the meta-analysis, based on predefined eligibility criteria. These analyses will consider only complete datasets for the outcomes of interest (executive functions), ensuring consistency across comparisons. Sensitivity analyses will be performed after the initial meta-analysis and whenever significant heterogeneity or risk of bias is detected. "

Reviewer #4:

Great efforts have been done in this manuscript and highlighted a specific condition regarding professors. Very good protocol and all the best.

R: Dear reviewer, We are immensely grateful for the review and encouragement provided.

Reviewer #5:

Dear authors,
Thank you for this opportunity to comment in your protocol.
I focus this time on the search strategies. The essential part of a systematic review to identify all potentially available evidence. In your protocol, you mentioned several databases. That is great. However, I was only able to check the search equations for PubMed. The current version does not align with a "good" search strategy that a medical librarian would do. I advise that you contact your library or have help from international services like systematicreviews.org. You are

missing MeSH terms but also need targeting the important field codes (like title and abstract of article in PubMed, where the body text is not searchable). It is recommended to have all search strategies available for the other databases for peer review and reproducibility issues. The prisma flow chart does not seem to be the latest from Page MJ (2020) available at https://www.prisma-statement.org/prisma-2020.

Authors' Response: Thank for your valuable and constructive feedback regarding our protocol, especially concerning the search strategies.

We fully agree with your observations. In the revised version of our manuscript, we are preparing a comprehensive Supplementary Material that will include the complete and detailed search strategies for all databases mentioned (including MeSH terms and appropriate field codes such as [tiab] for PubMed). This aims to ensure transparency, reproducibility, and adherence to best practices in systematic reviews.

Additionally, we will update the PRISMA flowchart according to the most recent 2020 version, as recommended.

The search strategies used both MeSH terms (Medical Subject Headings) and free-text terms (title and abstract) for each concept in the PICO framework. Below, we present the full search string used in PubMed, including MeSH descriptors.

Supplementary material

Pubmed:

("Teachers"[MeSH Terms] OR teacher*[tiab] OR professor*[tiab] OR educator*[tiab] OR instructor*[tiab] OR tutor*[tiab] OR lecturer*[tiab])

AND

("COVID-19"[MeSH Terms] OR "COVID-19"[tiab] OR coronavirus[tiab] OR "SARS-CoV-2"[tiab] OR "long COVID"[tiab] OR omicron[tiab] OR delta[tiab] OR "severe acute respiratory syndrome"[tiab])

AND

("Executive Function"[MeSH Terms] OR "Cognition"[MeSH Terms] OR "attention" OR "executive function*"[tiab] OR "cognitive control"[tiab] OR "working memory"[tiab] OR "cognitive inhibition"[tiab] OR "mental function*"[tiab] OR "frontal lobe function*"[tiab])

Scopus:

TITLE-ABS (teacher* OR professor* OR educator* OR instructor* OR tutor* OR lecturer*)

AND

TITLE-ABS ("COVID-19" OR coronavirus OR "SARS-CoV-2" OR "long COVID" OR omicron OR delta OR "severe acute respiratory syndrome")

AND

TITLE-ABS ("executive function*" OR "cognitive control" OR "working memory" OR "cognitive inhibition" OR "mental function*" OR "cognitive performance" OR "frontal lobe function*")

Web of science:

TS=(teacher* OR professor* OR educator* OR instructor* OR tutor* OR lecturer*)

AND

TS=("COVID-19" OR coronavirus OR "SARS-CoV-2" OR "long COVID" OR omicron OR delta OR "severe acute respiratory syndrome")

AND

TS=("executive function*" OR "cognitive control" OR "working memory" OR "cognitive inhibition" OR "mental function*" OR "frontal lobe function*" OR "cognitive performance")

Cochrane Library:

(teacher* OR professor* OR educator* OR instructor* OR tutor* OR lecturer*):ti,ab,kw

AND

("COVID-19" OR coronavirus OR "SARS-CoV-2" OR "long COVID" OR omicron OR delta OR "severe acute respiratory syndrome"):ti,ab,kw

AND

("executive function*" OR "cognitive control" OR "working memory" OR "cognitive inhibition" OR "mental function*" OR "cognitive performance" OR "frontal lobe function*"):ti,ab,kw

Embase:

('teacher'/exp OR teacher*:ti,ab OR professor*:ti,ab OR educator*:ti,ab OR instructor*:ti,ab OR tutor*:ti,ab OR lecturer*:ti,ab)

AND

('covid-19'/exp OR 'covid-19':ti,ab OR coronavirus:ti,ab OR 'sars-cov-2':ti,ab OR 'long covid':ti,ab OR omicron:ti,ab OR delta:ti,ab OR 'severe acute respiratory syndrome':ti,ab)

AND

('executive function'/exp OR 'cognition'/exp OR 'executive function*':ti,ab OR 'working memory':ti,ab OR 'cognitive performance':ti,ab OR 'cognitive inhibition':ti,ab OR 'mental function*':ti,ab OR 'frontal lobe function*':ti,ab)

ERIC:

(teacher* OR professor* OR educator* OR instructor* OR tutor* OR lecturer*)

AND

("COVID-19" OR coronavirus OR "SARS-CoV-2" OR "long COVID" OR omicron OR delta OR "severe acute respiratory syndrome")

AND

("executive function*" OR "cognitive control" OR "working memory" OR "cognitive inhibition" OR "mental function*" OR "frontal lobe function*" OR "cognitive performance")

SPORTdiscuss

(teacher* OR professor* OR educator* OR instructor* OR tutor* OR lecturer*)

AND

("COVID-19" OR coronavirus OR "SARS-CoV-2" OR "long COVID" OR omicron OR delta OR "severe acute respiratory syndrome")

AND

("executive function*" OR "cognitive control" OR "working memory" OR "cognitive inhibition" OR "mental function*" OR "frontal lobe function*" OR "cognitive performance")

LILACS:

(teacher OR professor OR educator OR instructor OR tutor OR lecturer)

AND

("COVID-19" OR coronavirus OR "SARS-CoV-2" OR "long COVID" OR omicron OR delta OR "síndrome respiratória aguda grave")

AND

("função executiva" OR "controle cognitivo" OR "memória de trabalho" OR "função mental" OR "desempenho cognitivo" OR "inibição cognitiva")

Reviewer #6:

1. Data Availability: Please specify where the data is available and for how long. If it is subjected to some institutional policy, please mention that, too. Please elaborate more.

<u>Authors' Response</u>: Thank you for your observation. The data from this systematic review will be made publicly available through the PROSPERO registry. All relevant materials—including the search strategy, data extraction form, and synthesis plans—will remain accessible on the PROSPERO platform for an indefinite period, following its open-access and transparency policies. No institutional restrictions apply to the availability of this information.

2. Abstract: The objective reads like that of a Qualitative study. It needs to be framed as a Review study's objective: "What is the body of literature on..." and so on..

Authors' Response: Thanks for your valuable suggestion. Adjusted into the manuscript, as follows:

"This systematic review protocol aims to identify, synthesize, and critically evaluate the existing body of literature on the effects of COVID-19 on educator's executive functions. "

Line 28: no need to mention 'Peers' , just reviewers would suffice.

Authors' Response: Thanks for your valuable suggestion. Adjusted into the manuscript , as follows:

"Data extraction and analysis will be performed according to the guidelines of the Preferred Reporting Items for Systematic Reviews and Meta-Analysis Protocols. The entire process will be carried out for two independent reviewers, with any discrepancies resolved by a third reviewer."

Line 30: Does it depend on the results? Or would it depend on the review question? Please clarify this, whether your review would assume a narrative synthesis or meta-analysis, in that case, you may have to include a quality checking of the included articles based on their methods. Or in other words, please mention why a risk of bias analysis is needed for the review, in light of your review objectives, Add sources, for the method/framework you will apply.

Authors' Response: Dear reviewer, we appreciate your comment. Our concern is that we may not find enough work to allow us to conduct a systematic review (or even no work at all). This is because this is a very specific population. In any case, we have adjusted the text to make this clear (line 34), as follows:

". If the search strategy yields a sufficient number of methodologically comparable studies" (...).

Line 127-128: Does not mention content: Exposure: CoVID-19 infection in inclusion criteria. Or time frame and language articles published in. Please mention everything and also a tentative idea about the time frame of search if the authors have an idea of that in advance.

Authors' Response: Thanks for your valuable suggestion. Adjusted into the manuscript, as follows:

"The eligibility criteria for this study were determined based on population, exposure, comparison, and outcome (PECO) (Tab. 2) (38, 39). Accordingly, the following

parameters are established: population (P), educators; exposure (E), have been infected with COVID-19; and outcome (O), executive function.

Table 2. Eligibility criteria.

	Inclusion	Exclusion
Population	Adult individuals (>18 years) who work as a teacher/educator at any educational levels	individuals diagnosed with psychiatric illnesses
Exposure	COVID-19 infection	- Not exposed to COVID-19 infection
Outcomes	Analysis of the executive functions of teachers who were infected with COVID-19	Studies that used non-validated methods to analyze executive functions<br><br>Self-reported executive functioning<br><br>Studies that analyzed executive functions outside the context of COVID-19
Study design	Cross-sectional, longitudinal; random and non-random studies	Literature reviews; studies with retractions

This systematic review will include articles published after the onset of the COVID-19 pandemic (March 11, 2020)."

4. No details on how search results will be recorded before any of the systematic steps start. Mention how yielded results will be recorded (which format- CSV/.Ref etc. , whether any software will be used (i.e. Excel / Endnote/ others reference managing softwares), how long data will be stored for, wheterh raw data of the search will be shared/ available for the readers,

Authors' Response: Dear reviewer, thank you for your comment. The information has been included in the methods, as follows:

"Search results will be exported from the databases in RIS format, stored and organized in Mendeley (reference manager), and subsequently imported into Rayyan software (https://new.rayyan.ai/) for screening and selection. The raw search data will be made available upon request, and all records will be retained indefinitely, in accordance

with institutional data management policies . The reviewers will carry out the analyses in parallel and using the "blind" feature. Subsequently, the data from the selected studies will be extracted independently by each reviewer".

5. Data extraction, synthesis and analysis: Mention how many reviewers in total will undertake each step and break-down each of the steps! (title abstract and full-text screening / reviewing/ data extraction/ analysis-synthesis), mention if same reviewers will be conducting all the steps.

R: Dear reviewer, thank you for your comment. The information has been included in the methods (lines: 185-208) , as follows:

"The study selection process will be conducted in three sequential phases: title screening, abstract screening, and full-text screening. All phases will be performed independently by two reviewers (LM and SM), and any disagreements will be resolved through discussion and, when necessary, adjudicated by a third reviewer (PS).

In the first phase, study titles will be screened against the predefined eligibility criteria. In the second phase, abstracts of the selected titles will be reviewed by the same two independent reviewers using the same inclusion and exclusion criteria. In the third phase, the full texts of potentially eligible studies will be retrieved and examined to confirm final inclusion (46). All three screening stages will be conducted independently and blindly between LM and SM.

A spreadsheet will be created to include all fields presented in Table 3 for data extraction. Two independent reviewers will evaluate and extract the data (LM and SM). Any discrepancies will be addressed by consensus with a third reviewer (PS). Data synthesis and analysis will also be performed by LM and SM, under the methodological supervision of PS. Depending on the volume and quality of the included studies, both qualitative synthesis and, where appropriate, meta-analytic methods (e.g., meta-analysis or meta-regression) will be applied. Quality appraisal and risk of bias assessments will be essential to inform the statistical approach and interpret the findings in alignment with the review's objectives.

Descriptive and content analyses of the results will also be conducted based on the impact of COVID-19 on educators' executive functions. The extracted data will include quantitative indicators such as means, standard deviations, and effect sizes related to executive function performance (e.g., working memory, cognitive flexibility, inhibitory control), as well as qualitative themes derived from narrative reports or interviews. Indices such as test scores, neuropsychological assessments, and self-reported measures will be used to categorize and synthesize findings".

6. Table 2 comes before Figure 1, though appears otherwise in text.

Authors' Response: Adjusted! Please see into the manuscript file.

7. The most critical point is probably the limitation of the review mentioned in the last paragraph (Line 253). If this is essentially the case (should the topic be so novel that the search results may not return any articles so as to appear as an 'Empty Review', then perhaps the authors should undertake a scoping review method which would serve the purpose better (provide an overview of literature on a relatively new topic, among pothers). In that case such elaborate risk-of-bias analysis plan do not have to be presented. This approach would also align with the idea that some preliminary search / in cases, a whole scoping review would take place before undertaking the actual Systematic Review. The authors may also consider broadening their search strategy/ operational definitions/ eligibility criteria to make sure some articles show up in search result!

Authors' Response: Thank you very much for this thoughtful comment. We truly appreciate the concern regarding the possibility of an "empty review" and the suggestion to consider a scoping review approach. However, we respectfully intend to maintain the systematic review design as originally proposed. This decision is grounded in our broader research plan, which includes an experimental field study focusing on the effects of COVID-19 on executive functions in university professors.

In this context, even a limited or null result from the systematic review will be also of scientific value. A potential "empty review" would highlight the existing research gap and serve as a strong justification and foundation for the field investigation currently underway. Furthermore, we understand the implications of such a result for evidence synthesis, and for that reason, we have designed a rigorous protocol to ensure transparency and methodological soundness, including risk of bias assessment and planned sensitivity analyses, should sufficient data be available.

We have also carefully considered our search strategy and eligibility criteria to balance specificity with comprehensiveness, aiming to capture relevant studies without compromising the review's focus. We remain open to refining the search strategy to maximize retrieval without altering the scope or nature of the review.

Once again, we appreciate your valuable input and the opportunity to clarify our approach.

I wish the authors best of luck in their decisions, publishing the protocol and finishing the review!
