# Supplementary material for: Consequences of COVID-19 infection on educators’ executive functions: A systematic review and meta-analysis protocol
Source: PLoS One. 2026 Oct 14;21(10):e0350156. doi: 10.1371/journal.pone.0350156 (PMC13667799; doi:10.1371/journal.pone.0350156)
Supplement: Supplementary file 4 [file pone.0350156.s006.pdf]

Dear reviewers, we are very grateful for your careful analysis and valuable comments. We use all of them to improve the methodological quality and scientific rigor of our work. Please see the answers to the questions below.

Reviewer #1: Thank you for the opportunity to review this protocol. I hope the above comments are helpful in refining and strengthening the manuscript prior to publication.

Keywords: It is recommended not to repeat keywords that are already in the title, in order to increase the paper's reach and discoverability.

Authors' Response: Adjusted. Thanks for your suggestion. Please see key-words, as follows:

"Key-words: Executive control; SARS-COV-2; public health; educational."

Abstract: Since this is a protocol, it would be more appropriate to end the abstract with a clear conclusion rather than including a discussion, which is not relevant in this context.

Authors' Response: Adjusted. Please see abstract, as follows:

"Conclusion: This systematic review is expected to deepen understanding of COVID-19's impact on educator's health, with a focus on analyzing executive functions. "

Introduction:

Line 57 of the introduction, the phrase that says "including education" and then continues discussing neuroinflammation seems to lack meaning, I suggest reviewing the wording.

Authors' Response: Adjusted. Please see introduction, as follows:

"The coronavirus disease (COVID-19) has led to significant challenges in the world, affecting various aspects of daily life (1). While much attention has been given to the respiratory and systemic effects of the virus, increasing evidence suggests that COVID-19 also affects the central nervous system (CNS) (2). There are also reports of psychosocial issues, leading to disorders such as anxiety and depression (3, 4). Therefore, we are aware that the extent of the virus infection and the pandemic context experience have broad repercussions. This suggests the need for greater in-depth analysis of the long-term consequences for the population."

Line 63: The text refers to "three core executive functions" but then lists four; this inconsistency should be corrected.

Authors' Response: Sorry for this mistake. Adjusted. Please see introduction, as follows:

The literature has identified three central EFs (26): i) inhibitory control (i.e., managing inappropriate behaviors (17, 18) and maintaining selective attention (26, 27)), ii) working memory (i.e., retention and manipulation of information) (28), and ii) cognitive flexibility (i.e., adaptation to new rules (18) and perspectives (10).

Line 73: The term “other studies” is used, but the same author previously cited is referenced again, which may be confusing.

Authors’ Response: Adjusted. To make clearer and meet the scientific writing requirements, we removed the previously author, keeping only the others. Please see introduction, as follows:

“A report explained that COVID-19 led to impairments in executive function in 36% of cases (32). Other studies have shown similar results, indicating that COVID-19 infection may lead to disorders related to headache, confusion and attention difficulties (21, 23).”

The introduction should explicitly mention previous systematic reviews and meta-analyses that have addressed similar topics, and clearly explain what novelty this review adds.

Authors’ Response: Dear reviewer, We agree with your requestion and have added a paragraph to address it. Please see introduction, as follows:

It's worth mentioning that systematic reviews related to this topic have already been published (35-38). Korzeniowski sought to understand the impacts of COVID-19 on children's executive functions and found significant impacts on mental health, well-being, and quality of life (35). Another relevant study reviewed the neurocognitive profile of people infected with the virus (36). The authors concluded that executive functions are significantly impaired in people infected with long-term COVID, resulting in reduced performance on tasks that require the use of cognitive capacity. A third systematic review sought to understand the relationship between COVID-19 infection and the incidence of cognitive deficits, finding a high frequency of decline in cognitive capacity after infection (37). Finally, a systematic review evaluated the cognitive effects of the infection on adults. The study concluded that significant impairments exist, but indicated that the pathological mechanisms underlying this are still unknown (38).

Methods:

Lines 91–92: A preliminary search is mentioned, but no findings are reported. It would be useful to briefly state what was identified.

Authors’ Response: Thanks for your comment. We didn't actually present a preliminary study, but rather reported that a preliminary search will be conducted in the methodological steps for systematic review. As follows:

“After defining the research problem, a preliminary search will be conducted in the databases to identify studies that form the basis of the research”.

Line 100: Please clarify that the full systematic search will be conducted once the protocol is published.

Authors’ Response: Adjusted. Please see introduction, as follows:

“the full systematic search will be conducted once the protocol is published”.

Lines 104–107: Consider removing the explanation of Boolean operators “OR” and “AND,” as it is unnecessary for a scholarly audience.

Authors’ Response: Suggestion accepted. See the excerpt after deletion:

“This systematic review employed the following Medical Subject Headings terms as the primary search terms: "COVID-19" and "executive function”. The search strategy will comprise three blocks that refer to the public (educators), exposure (COVID-19), and outcome (executive functions), as shown in Table 1”.

Table 1:

It is suggested to include the exact search strategies used for each database (copy-pasted) to enhance reproducibility.

Authors’ Response: Dear reviewer, we have chosen to submit this material as a supplementary document for publication.

The use of the asterisk (*) appears inconsistent across terms; please clarify the rationale.

Authors’ Response: Thank you for your insightful observation regarding the use of the asterisk (*) in our search strategies. Indeed, the asterisk was employed as a truncation operator to broaden the retrieval of terms sharing the same root, where such functionality is supported by the respective database.

The apparent inconsistency in the use of truncation across terms and databases can be explained by two main factors:

1- Differences among database platforms: Some databases—such as **LILACS**, **PubMed (using MeSH)**, and **Embase**—handle indexed terms differently or do not interpret truncation in the same way. Therefore, in these cases, we chose to use exact terms or controlled vocabulary descriptors to ensure alignment with the database’s structure and indexing system.

2- Adaptation to the technical capabilities of each platform: In databases that allow truncation in title/abstract fields (e.g., Scopus, Web of Science, ERIC), the asterisk was intentionally included to ensure comprehensive retrieval of word variants. In contrast, for databases like LILACS, where truncation may not function reliably or optimally, we opted to use explicit singular and plural forms where necessary.

In this way, each search strategy was carefully tailored to the specific features and capabilities of the database, aiming to balance both sensitivity and specificity in the identification of relevant studies.

Line 116: Add the appropriate citation for the PRISMA-S guideline.

Authors’ Response: Adjusted. Please see as follows:

“The metadata extracted from the databases adhered to the Preferred Reporting Items for Systematic Reviews and Meta-Analyses (PRISMA-S) search checklist (42). “

Reference:

Pérez-Neri I, Pineda C, Flores-Guerrero JL, Estêvão MD, Vasanthan LT, Lorente S, et al. Adherence to literature search reporting guidelines in leading rheumatology journals' systematic reviews: umbrella review protocol. Rheumatol Int. 2022; 42: 2135–2140. https://doi.org/10.1007/s00296-022-05194-1 PMID: 36029320

Line 117: Include a link to the Rayyan platform.

Authors' Response: Adjusted. Please see methods, as follows:

"Search results will be exported from the databases in RIS format, stored and organized in Mendeley (reference manager), and subsequently imported into Rayyan software (https://new.rayyan.ai/) for screening and selection."

Line 118: Mention that references from other systematic reviews will also be screened.

Authors' Response: Adjusted. Please see methods, as follows:

"It is important to emphasize that references from other systematic reviews will also be screened."

Eligibility Criteria (Line 121):

This section should be specified much more clearly. I suggest using a table and reviewing inclusion and exclusion criteria for each PECO component, and adding an "S" for the type of study. It is very important to determine how executive functions will be evaluated, and what will be considered an educator or teacher (from any level?). The exposure must be clearly defined — will only longitudinal studies with educators who have been exposed be included? Or will studies that simply mention COVID also be considered?

Authors' Response:

We appreciate your thoughtful and constructive comments regarding the clarification of the eligibility criteria using the PECO(S) framework. Indeed, we recognize the value of specifying each component more rigorously, including clearer operational definitions of exposure, outcome, and population, as well as the study design.

However, considering the exploratory nature of our review and the anticipated low volume of eligible studies on this specific topic (educators, COVID-19 exposure, and executive functions), we have intentionally adopted broader inclusion criteria to avoid excessively restricting the sample. Imposing additional parameters at this stage—such as limiting to longitudinal designs or requiring standardized instruments across all studies—might significantly hinder the feasibility and comprehensiveness of the review.

Our goal is to capture the current state of the evidence in this emerging field. Therefore, we opted to include both self-reported and performance-based measures of executive functions and to consider all educational levels and teaching contexts. Similarly, studies that clearly mention COVID-19 exposure among educators are considered eligible, even if not exclusively longitudinal.

We believe this broader approach will enable a more inclusive synthesis of available evidence, which may in turn inform more refined future research. Nonetheless, we acknowledge the importance of methodological rigor and will transparently report the heterogeneity and limitations of the included studies in our analysis and discussion.

Figures and Tables:

Line 144: Figure 1 is not visible; please ensure it is included properly.

Authors' Response: Dear reviewer, Figure 1 was submitted separately. However, due to your request, we've included it in the text. Please let me know what you think.

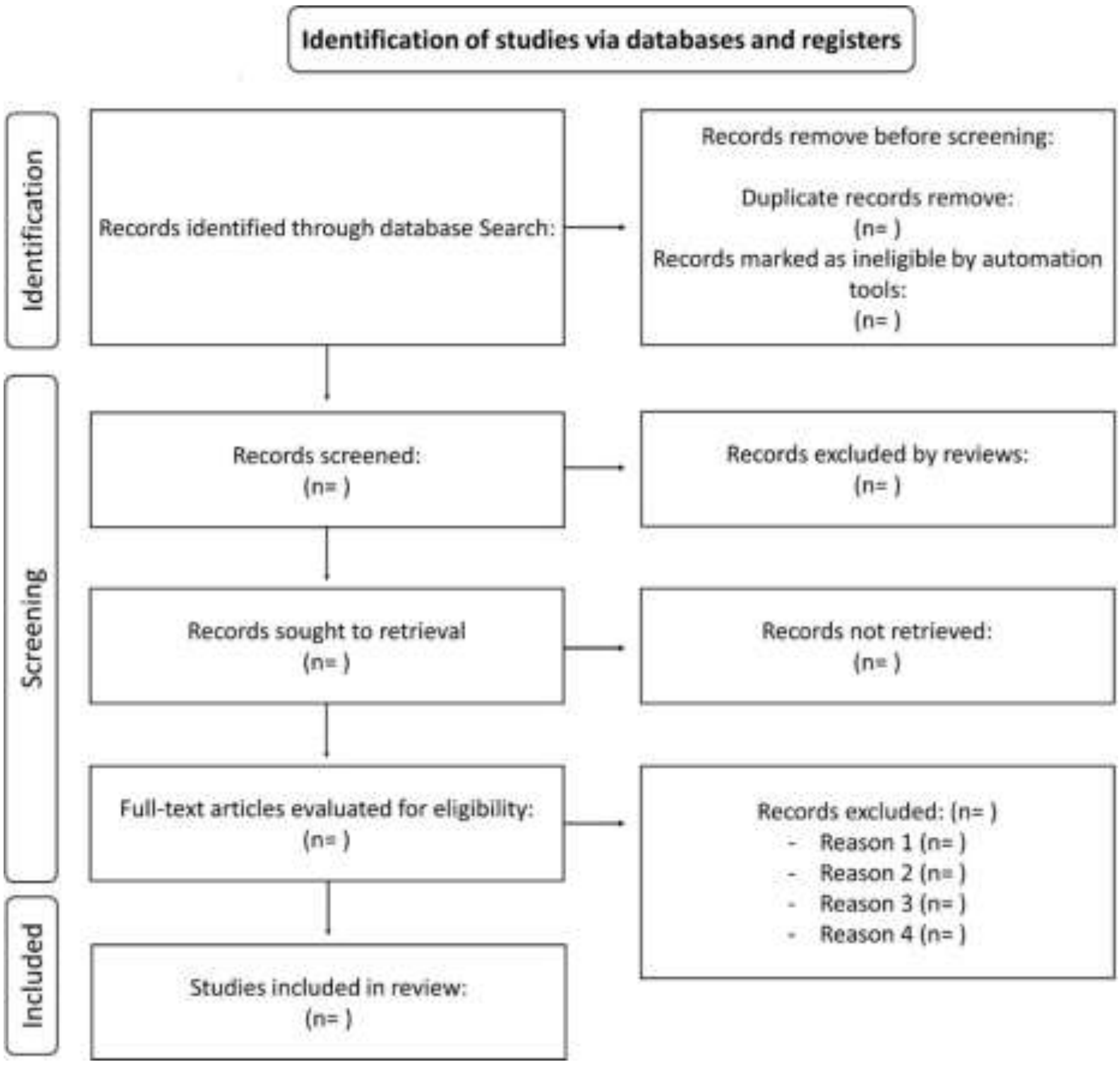

Line 151: Table 2 is not mentioned in the main text; it should be properly referenced.

Authors' Response: Adjusted. Please see methods, as follows:

"The eligibility criteria for this study were determined based on population, exposure, comparison, and outcome (PECO) (Tab. 2)".

Risk of Bias Assessment (Line 161): Add the appropriate heading for this section.

Authors' Response: Adjusted. Please see methods, as follows:

“Risk of bias”
“The risk of bias will be evaluated using the 27-item Downs and Black scale (...)”

It is not possible to evaluate the proposed bias assessment tool without a clear statement on the study designs that will be included.

Authors’ Response:

Dear reviewer, based on your comment, we have modified this section. Given the emerging and multidisciplinary nature of this topic, we anticipate a potentially limited number of eligible studies. Therefore, we have chosen not to predefine strict restrictions regarding study design at this stage, in order to avoid excluding potentially relevant contributions. Eligible studies may include cross-sectional, longitudinal, or other non-randomized designs.

Consequently, the risk of bias will be assessed using the tool most appropriate to each study design, following established methodological guidance. This approach allows flexibility and ensures methodological rigor across different types of evidence. The tools applied will be clearly stated and justified in the final manuscript, along with a transparent discussion of the limitations and heterogeneity of the included studies.

Please see the modified excerpt:
“The risk of bias will be assessed by two independent reviewers, and any disagreements will be resolved by consulting a third reviewer. Given the potential methodological heterogeneity of the included studies, validated tools will be applied according to the specific study design For observational studies, the Joanna Briggs Institute (JBI) critical appraisal tools will be used; for randomized controlled trials, the Cochrane Risk of Bias Tool (RoB 2.0); for qualitative studies, the JBI Checklist for Qualitative Research; and for mixed-methods studies, the Mixed Methods Appraisal Tool (MMAT).

In cases where the Downs and Black checklist is appropriate—particularly for quantitative studies with both randomized and non-randomized elements—it may also be applied (54). A quality score will be calculated for each study and expressed as a percentage of the maximum possible score for that design. Studies scoring 70% or higher will be considered to have a low risk of bias (55). “

Data Transformation (Line 168): This section is unclear. What data will be transformed exactly? What data will be extracted specifically for each of the sections mentioned in Table 2 (e.g., mean, SD…?) Please clarify.

Authors’ Response: Dear reviewer, due to the lack of clarity, we have decided to remove this topic. Once we have the data, if necessary, we will perform this type of procedure and clarify it in the final manuscript (systematic review).

Meta-Analysis (Line 173): The planned statistical analyses should be described in greater detail and with more technical specificity.

Authors’ Response: Adjusted. Please see methods, as follows:

“All main analyses will consider studies that have investigated the impact of COVID-19 on executive functions (EFs) among educators. Accordingly, the primary outcome of interest is the assessment of executive functioning post-COVID-19 infection.

Analyses will be stratified by study design: cross-sectional and longitudinal (cohort) studies will be analyzed separately to account for differences in methodological structure and inference potential. If sufficient data are available, separate meta-analyses will be conducted for each design type.

Effect sizes (ES) for continuous outcomes will be extracted or calculated (e.g., using means and standard deviations) and reported as standardized mean differences (SMDs) with corresponding 95% confidence intervals (CIs). When studies provide adjusted estimates (e.g., odds ratios or regression coefficients), these will also be considered, and their inclusion will be justified in sensitivity analyses.

Meta-analyses will be performed using the random-effects model (DerSimonian and Laird method), given the expected clinical and methodological heterogeneity across studies. Analyses will be conducted using R software (metafor and meta packages).

Heterogeneity will be assessed using the I^2 statistic (with thresholds: 25% = low, 50% = moderate, and 75% = high heterogeneity) and Cochran's Q test ($p < 0.10$ considered significant). Where substantial heterogeneity is detected, subgroup analyses and/or meta-regression will be conducted to explore potential effect modifiers.

Subgroup analyses will be performed based on variables such as age group, gender, socioeconomic status, years of teaching experience, and level of education (e.g., primary, secondary, higher education). These variables will also be considered for meta-regression, provided a sufficient number of studies ($k \geq 10$) report the necessary data.”

Sensitivity Analysis (Line 186): What exactly will be reported? Will every individual analysis be described? What thresholds will be used to judge quality? Further elaboration is needed.

Authors’ Response: Adjusted. Please see methods, as follows:

“Sensitivity analyses will be conducted to evaluate the robustness and reliability of the meta-analytic results. The primary sensitivity analysis will use a leave-one-out approach, systematically excluding each individual study one at a time to assess its influence on the overall pooled effect size and confidence intervals. This will identify any studies disproportionately affecting the results or driving the observed effect (56).

Additionally, sensitivity analyses will exclude studies classified as having a high risk of bias, based on the predefined criteria of each risk of bias assessment tool applied (e.g., studies scoring below 70% on the Downs and Black scale or rated as “high risk” on the Cochrane RoB 2.0 tool). The impact of excluding these studies on pooled estimates will be reported. Further sensitivity analyses will be restricted to studies providing complete datasets for the primary outcomes (executive functions) to ensure comparability and reduce potential bias from missing data.

All sensitivity analyses will be fully described in the results section, including the specific studies excluded, changes in effect sizes, confidence intervals, and heterogeneity statistics (I^2). Any substantial changes in the direction or magnitude of the effect will be explicitly discussed. Sensitivity analyses will be performed after the initial meta-analysis and will also be conducted whenever significant heterogeneity ($I^2 > 50\%$) or risk of bias concerns are identified.”

Limitations (Line 254): The text states that relevant studies may not be found, but earlier a preliminary search was said to have been conducted. This appears contradictory and should be clarified.

Authors' Response: Thank you for your comment. Although we conducted a preliminary search, we recognize that a systematic search may still retrieve a limited number of studies due to the specificity of the topic. We've adjusted the text to clarify this. Please see as follows:

"A potential limitation of this protocol is the possibility of retrieving a limited number of studies after applying the database search strategies, which may restrict the breadth of the synthesis. Nonetheless, even a small number of studies can provide valuable insights and highlight existing gaps in the literature that warrant further investigation (47, 64). Another limitation that may arise is the great variability in the characteristics of the population used as a sample, leading to heterogeneity, which makes the synthesis and analysis of results more complex. One proposal to alleviate this issue is to classify the participants into subgroups to perform statistical analyses."

Reviewer #5: Dear Authors, thank you for this revised version. I have few comments. In a table, you mentioned type of eligible studies as "random and non-random", please modify to "randomized and non-randomized". However, for ethical reasons, I argue that there will be no randomized controlled trials on this topic. This might be said somewhere in the protocol.

Authors' response: Dear reviewer, thank you for your thoughtful review. The adjustment has been made. See the table.

	Inclusion	Exclusion
Population	Adult individuals (>18 years) who work as a teacher/educator at any educational levels	individuals diagnosed with psychiatric illnesses
Exposure	COVID-19 infection	- Not exposed to COVID-19 infection
Outcomes	Analysis of the executive functions of teachers who were infected with COVID-19	Studies that used non-validated methods to analyze executive functions<br><br>Self-reported executive functioning<br><br>Studies that analyzed executive functions outside the context of COVID-19
Study design	Cross-sectional, longitudinal; randomized and non-randomized studies	Literature reviews; studies with retractions

Please revise the English language for your protocol: "carried out for" seems weird: "carried out by", is my suggestion. The statement that cite ref. 9.10,11 and 12, needs to be more clear and

fluent. The website of Rayyan does not naturally contains the subdomain "news". Please also cite it as Mourad Ouzzani, Hossam Hammady, Zbys Fedorowicz, and Ahmed Elmagarmid. Rayyan — a web and mobile app for systematic reviews. Systematic Reviews (2016) 5:210, DOI: 10.1186/s13643-016-0384-4.

Authors' response: Dear reviewer, thank you for your thoughtful review. The adjustment has been made, as follow:

“Search results will be exported from the databases in RIS format, stored and organized in Mendeley (reference manager), and subsequently imported into Rayyan software (http://rayyan.qcri.org)- a web and mobile app for systematic reviews- for screening and selection (43).”

You replied to a reviewer by saying you are going to "narrowing the analyses": you meant subgroup analyses. You mentioned this later in detail but make sure that the reader gets such information well in advance. YOu may add the code field "kw" in Embase as :ab,ti,kw. You may also improve the syntax and vocabulary to cover all potentially availabel evidence.

Proofread your protocol and good luck.

Author’s response: Thanks for your important comment. we've carefully analyzed our search strategy and made adjustments. Please see below:

PubMed

("Teachers"[MeSH Terms] OR teacher[tiab] OR teachers[tiab] OR professor[tiab] OR professors[tiab] OR educator[tiab] OR educators[tiab] OR instructor[tiab] OR instructors[tiab] OR tutor[tiab] OR tutors[tiab] OR lecturer[tiab] OR lecturers[tiab])

AND

("COVID-19"[MeSH Terms] OR "COVID-19"[tiab] OR coronavirus[tiab] OR "SARS-CoV-2"[tiab] OR "long COVID"[tiab] OR omicron[tiab] OR delta[tiab] OR "severe acute respiratory syndrome"[tiab])

AND

("Executive Function"[MeSH Terms] OR "Cognition"[MeSH Terms] OR attention[tiab] OR "executive function"[tiab] OR "executive functions"[tiab] OR "cognitive control"[tiab] OR "working memory"[tiab] OR "cognitive inhibition"[tiab] OR "mental function"[tiab] OR "mental functions"[tiab] OR "frontal lobe function"[tiab] OR "frontal lobe functions"[tiab])

Scopus

TITLE-ABS (teacher* OR professor* OR educator* OR instructor* OR tutor* OR lecturer*)

AND

TITLE-ABS ("COVID-19" OR coronavirus OR "SARS-CoV-2" OR "long COVID" OR omicron OR delta OR "severe acute respiratory syndrome")

AND

TITLE-ABS ("executive function*" OR "cognitive control" OR "working memory" OR "cognitive inhibition" OR "mental function*" OR "frontal lobe function*" OR "cognitive performance")

Web of Science

TS=(teacher* OR professor* OR educator* OR instructor* OR tutor* OR lecturer*)

AND

TS=("COVID-19" OR coronavirus OR "SARS-CoV-2" OR "long COVID" OR omicron OR delta OR "severe acute respiratory syndrome")

AND

TS=("executive function*" OR "cognitive control" OR "working memory" OR "cognitive inhibition" OR "mental function*" OR "frontal lobe function*" OR "cognitive performance")

Cochrane Library

(teacher* OR professor* OR educator* OR instructor* OR tutor* OR lecturer*):ti,ab,kw

AND

("COVID-19" OR coronavirus OR "SARS-CoV-2" OR "long COVID" OR omicron OR delta OR "severe acute respiratory syndrome"):ti,ab,kw

AND

("executive function*" OR "cognitive control" OR "working memory" OR "cognitive inhibition" OR "mental function*" OR "frontal lobe function*" OR "cognitive performance"):ti,ab,kw

Embase

('teacher'/exp OR teacher*:ti,ab OR professor*:ti,ab OR educator*:ti,ab OR instructor*:ti,ab OR tutor*:ti,ab OR lecturer*:ti,ab)

AND

('covid-19'/exp OR 'covid-19':ti,ab OR coronavirus:ti,ab OR 'sars-cov-2':ti,ab OR 'long covid':ti,ab OR omicron:ti,ab OR delta:ti,ab OR 'severe acute respiratory syndrome':ti,ab)

AND

('executive function'/exp OR 'cognition'/exp OR 'executive function*':ti,ab OR 'working memory':ti,ab OR 'cognitive performance':ti,ab OR 'cognitive inhibition':ti,ab OR 'mental function*':ti,ab OR 'frontal lobe function*':ti,ab)

ERIC

(teacher* OR professor* OR educator* OR instructor* OR tutor* OR lecturer*)

AND

("COVID-19" OR coronavirus OR "SARS-CoV-2" OR "long COVID" OR omicron OR delta OR "severe acute respiratory syndrome")

AND

("executive function*" OR "cognitive control" OR "working memory" OR "cognitive inhibition" OR "mental function*" OR "frontal lobe function*" OR "cognitive performance")

SPORTDiscus

(teacher* OR professor* OR educator* OR instructor* OR tutor* OR lecturer*)

AND

("COVID-19" OR coronavirus OR "SARS-CoV-2" OR "long COVID" OR omicron OR delta OR "severe acute respiratory syndrome")

AND

("executive function*" OR "cognitive control" OR "working memory" OR "cognitive inhibition" OR "mental function*" OR "frontal lobe function*" OR "cognitive performance")

LILACS

(teacher OR teachers OR professor OR professors OR educator OR educators OR instructor OR instructors OR tutor OR tutors OR lecturer OR lecturers)

AND

("COVID-19" OR coronavirus OR "SARS-CoV-2" OR "long COVID" OR omicron OR delta OR "severe acute respiratory syndrome")

AND

("executive function" OR "executive functions" OR "cognitive control" OR "working memory" OR "cognitive inhibition" OR "mental function" OR "mental functions" OR "frontal lobe function" OR "frontal lobe functions" OR "cognitive performance")

#Reviewer 6

Much care should be taken in detailing out the analysis plan, themes, sub-themes etc.

reporting guidelines are yet to be developed for empty reviews, so need to be cautious.

Discussion and conclusions must be evidence -based.

Author's response: We sincerely appreciate your valuable comments and thoughtful guidance regarding the analysis plan and the challenges associated with empty reviews. In response, we have thoroughly reviewed and refined our methodology to ensure clarity and appropriateness, maintaining only the elements we consider essential at this stage. Naturally, certain aspects of the analysis and reporting may be adapted as necessary based on the data retrieved during the systematic search. We remain committed to grounding our discussion and conclusions strictly in the evidence obtained.
