# Supplementary material for: Consequences of COVID-19 infection on educators’ executive functions: A systematic review and meta-analysis protocol
Source: PLoS One. 2026 Oct 14;21(10):e0350156. doi: 10.1371/journal.pone.0350156 (PMC13667799; doi:10.1371/journal.pone.0350156)
Supplement: Supplementary file 5 [file pone.0350156.s002.docx]

**PONE-D-25-03730**

1. Data Availability: Please specify where the data is available and for how long. If it is subjected to some institutional policy, please mention that, too. Please elaborate more.

2. Abstract: The objective reads like that of a Qualitative study. It needs to be framed as a Review study's objective: "What is the body of literature on..." and so on..

Line 28: no need to mention 'Peers' , just reviewers would suffice.

Line 30: Does it depend on the results? Or would it depend on the review question? Please clarify this, whether your review would assume a narrative synthesis or meta-analysis, in that case, you may have to include a quality checking of the included articles based on their methods. Or in other words, please mention why a risk of bias analysis is needed for the review, in light of your review objectives, Add sources, for the method/framework you will apply.

3. Line 127-128: Does not mention content: Exposure: CoVID-19 infection in inclusion criteria. Or time frame and language articles published in. Please mention everything and also a tentative idea about the time frame of search if the authors have an idea of that in advance.

4. No details on how search results will be recorded before any of the systematic steps start. Mention how yielded results will be recorded (which format- CSV/.Ref etc. , whether any software will be used (i.e. Excel / Endnote/ others reference managing softwares), how long data will be stored for, wheterh raw data of the search will be shared/ available for the readers,

5. Data extraction, synthesis and analysis: Mention how many reviewers in total will undertake each step and break-down each of the steps! (title abstract and full-text screening / reviewing/ data extraction/ analysis-synthesis), mention if same reviewers will be conducting all the steps.

6. Table 2 comes before Figure 1, though appears otherwise in text.

7. The most critical point is probably the limitation of the review mentioned in the last paragraph (Line 253). If this is essentially the case (should the topic be so novel that the search results may not return any articles so as to appear as an ‘Empty Review’, then perhaps the authors should undertake a scoping review method which would serve the purpose better (provide an overview of literature on a relatively new topic, among pothers). In that case such elaborate risk-of-bias analysis plan do not have to be presented. This approach would also align with the idea that some preliminary search / in cases, a whole scoping review would take place before undertaking the actual Systematic Review. The authors may also consider broadening their search strategy/ operational definitions/ eligibility criteria to make sure some articles show up in search result!

I wish the authors best of luck in their decisions, publishing the protocol and finishing the review!
