# Supplementary material for: Consequences of COVID-19 infection on educators’ executive functions: A systematic review and meta-analysis protocol
Source: PLoS One. 2026 Oct 14;21(10):e0350156. doi: 10.1371/journal.pone.0350156 (PMC13667799; doi:10.1371/journal.pone.0350156)
Supplement: Supplementary file 6 [file pone.0350156.s007.docx]

**Response to the Reviewer and Editor:**

Thank you for considering our manuscript and for the opportunity to revise and resubmit it. We have carefully reviewed the reviewers’ comments and your editorial guidance and made substantial revisions to improve the manuscript, including minor linguistic revisions. The responses to the reviewers were prepared separately and attached. All revisions are highlighted in **red** color using track changes for transparency.

We want to express our sincere gratitude for the opportunity to revise and resubmit our manuscript, as well as for the continued coordination of the peer review process. We would like to extend our special thanks to both reviewers for the time and dedication they invested in providing their valuable feedback.

**Reviewer #1:** Thank you for addressing all my comments. I think the manuscript has improved considerably, but I still think that not all the comments have been fully resolved, perhaps due to some confusion. Here are the minor revisions that the study would need:

Comment 1: Objective: I believe that the objective, even if changed, still does not correspond to what is being done. As this study corresponds to a protocol, the objective should not focus on synthesizing and critically evaluating the existing evidence, as that will be the result of the future review. The objective should be to establish the methodological guidelines and criteria for carrying out this review. You can also point out that the objective of the future review will be to synthesize, etc.

**Authors’ Response:** Adjusted. Please see introduction, as follows:

“To the best of our knowledge, no systematic review has addressed this issue (39, 40). The guiding research question was the following: “Does COVID-19 affect teachers' EFs in the long term?” This knowledge can enhance our scientific understanding of the topic because EFs represent an essential element for teaching activities. Thus, The objective of this protocol is to describe the methodological procedures, eligibility criteria, search strategy, and analytical approach that will be used to conduct a systematic review and meta-analysis examining the consequences of COVID-19 infection on educators’ executive functions.”

Comment 2: Data and indices to be used: I appreciate the adjustments made, however, I believe that the description still needs to be more specific. It is not enough to state that means, standard deviations, effect sizes, or neuropsychological tests in general will be included; it is necessary to indicate more precisely: When will a relevant difference be considered to exist (e.g., statistical significance thresholds, minimum effect sizes, or clinical criteria), what specific estimators will be taken into account (95% CI, p-values, Cohen's d, OR, RR, etc.)... and all sources supporting this use must be referenced.
Comment 3: Risk of bias: The clarification is adequate, but references are needed to justify the choice of the 70% threshold for considering low risk of bias. This criterion should be supported by recognized literature or methodological guidelines. Similarly, for each of the tools mentioned (RoB 2.0, JBI, MMAT, etc.), it would be advisable to include the corresponding reference, so that the theoretical and methodological basis for their application is clear. This will provide transparency and facilitate the reproducibility of the assessment.

**Authors’ Response:** Thank you for thess suggestions. We have revised the section to specify the statistical thresholds for significance (p < 0.05), the interpretation criteria for effect sizes (Cohen’s benchmarks), and the exact estimators to be used (Hedges’ g, OR, RR, 95% CI). We also clarified the criteria for conducting subgroup analyses and meta-regression (k ≥ 10) and included appropriate methodological references. Please see the text, as follows:

**“Main meta-analyses and/or meta-regressions**

The primary outcome will be post-COVID-19 executive function performance in educators, assessed through validated neuropsychological tests (e.g., working memory, inhibitory control, cognitive flexibility measures). For continuous outcomes, effect sizes will be calculated as standardized mean differences (Hedges’ g) with 95% confidence intervals (95% CI). Statistical significance will be defined as p < 0.05 (two-tailed). Effect size magnitude will be interpreted according to Cohen’s criteria (0.20 = small, 0.50 = moderate, 0.80 = large) (57).

For dichotomous outcomes, pooled estimates will be expressed as odds ratios (OR) or risk ratios (RR) with 95% CI. When adjusted estimates are available, the most fully adjusted model will be extracted (57).

A random-effects model (DerSimonian and Laird estimator) will be used. Between-study heterogeneity will be quantified using I² (25% low, 50% moderate, 75% high) and Cochran’s Q test (p < 0.10 indicating significant heterogeneity) (58). Subgroup analyses and meta-regression will be conducted when k ≥ 10 studies are available per covariate (59).

All analyses will be conducted in R (metafor and meta packages). Methodological decisions are based on established meta-analytic guidelines (57).”

Comment 4: Exact date of the search: It is not enough to indicate only the month in which the search was conducted. It is essential to indicate the exact date (day, month, and year) on which each search will be performed in the databases, which I also suggest should be after the protocol has been accepted.

**Authors’ Response:** Adjusted. Thank you for this clarification. We have revised the manuscript to specify the exact date on which the database searches will be conducted (10 April 2026). We also clarified that the searches will be performed after protocol acceptance to ensure transparency and inclusion of the most recent evidence. Please, se as follow:

“The electronic database searches will be conducted on 10 April 2026, following acceptance of this protocol, to ensure methodological transparency and to capture the most up-to-date evidence available at the time of the review.”

Comment 7: Search strategy: I believe my previous comment has not been fully understood. I agree with the format currently presented and understand that you want to keep the tables included, but what I am requesting is not the eligibility criteria (as incorporated in Table 2), but the exact step-by-step search strategy. To ensure replicability, you need to include the complete search strings as they were executed in each database. The example I wanted to point out was not the table in the cited article, but the table in the appendix to that work (https://doi.org/10.1371/journal.pone.0296273) , which details the specific search strategies. I therefore suggest keeping the tables you already have, but adding an additional table (or an appendix) that explicitly reproduces each search strategy used in the different databases.

**Authors’ Response:** Thank you for the clarification. We have now added a separate appendix table that provides the complete, database-specific search strings exactly as executed in each source, ensuring full transparency and reproducibility. The original tables have been maintained, and the additional appendix explicitly details the step-by-step search strategies, as suggested. Please, see as follow:

**Terms used in the search strategy according to each database.**

| Database | Search strategy |
| --- | --- |
| Cochrane Library | ([mh "School Teachers"] OR [mh "Faculty"] OR teacher*:ti,ab,kw OR professor*:ti,ab,kw OR educator*:ti,ab,kw OR instructor*:ti,ab,kw OR tutor*:ti,ab,kw OR lecturer*:ti,ab,kw) AND ([mh "COVID-19"] OR [mh "SARS-CoV-2"] OR [mh "Post-Acute COVID-19 Syndrome"] OR "COVID-19":ti,ab,kw OR "SARS-CoV-2":ti,ab,kw OR coronavirus:ti,ab,kw OR "long COVID":ti,ab,kw) AND ([mh "Executive Function"] OR [mh "Cognition"] OR [mh "Neuropsychological Tests"] OR "executive function*":ti,ab,kw OR "working memory":ti,ab,kw OR "cognitive control":ti,ab,kw OR "cognitive flexibility":ti,ab,kw) |
| Pubmed | ("School Teachers"[Mesh] OR "Faculty"[Mesh] OR teacher*[tiab] OR professor*[tiab] OR educator*[tiab] OR instructor*[tiab] OR tutor*[tiab] OR lecturer*[tiab] OR faculty[tiab]) AND ("COVID-19"[Mesh] OR "SARS-CoV-2"[Mesh] OR "Post-Acute COVID-19 Syndrome"[Mesh] OR COVID-19[tiab] OR SARS-CoV-2[tiab] OR coronavirus[tiab] OR "long COVID"[tiab] OR "post-acute COVID"[tiab]) AND ("Executive Function"[Mesh] OR "Cognition"[Mesh] OR "Neuropsychological Tests"[Mesh] OR "Cognition Disorders"[Mesh]OR "executive function*"[tiab] OR "cognitive control"[tiab] OR "working memory"[tiab] OR "cognitive inhibition"[tiab] OR "cognitive flexibility"[tiab] OR "cognitive performance"[tiab]) |
| Scopus | TITLE-ABS-KEY(teacher* OR professor* OR educator*  OR instructor* OR tutor* OR lecturer* OR faculty) AND TITLE-ABS-KEY("COVID-19" OR "SARS-CoV-2" OR coronavirus OR "long COVID" OR "post-acute COVID") AND TITLE-ABS-KEY("executive function*" OR "cognitive control" OR "working memory" OR "cognitive inhibition"  OR "cognitive flexibility"OR "cognitive performance") |
| Web of Science | TS=(teacher* OR professor* OR educator* OR instructor* OR tutor* OR lecturer* OR faculty) AND TS=("COVID-19" OR "SARS-CoV-2" OR coronavirus OR "long COVID" OR "post-acute COVID") AND TS=("executive function*" OR "cognitive control" OR "working memory" OR "cognitive inhibition" OR "cognitive flexibility"OR "cognitive performance") |
| EMBASE | ('school teacher'/exp OR 'faculty member'/exp OR teacher*:ti,ab OR professor*:ti,ab OR educator*:ti,ab OR instructor*:ti,ab OR tutor*:ti,ab OR lecturer*:ti,ab) AND('covid-19'/exp OR 'sars-related coronavirus 2'/exp OR 'post-acute covid-19 syndrome'/exp OR covid-19:ti,ab OR sars-cov-2:ti,ab OR coronavirus:ti,ab OR 'long covid':ti,ab) AND('executive function'/exp OR 'cognition'/exp OR 'neuropsychological test'/exp OR 'cognitive defect'/exp  OR 'working memory'/exp OR 'cognitive control':ti,ab OR 'cognitive flexibility':ti,ab OR 'cognitive inhibition':ti,ab OR 'cognitive performance':ti,ab) |
| ERIC | (teacher* OR professor* OR educator* OR instructor* OR tutor* OR lecturer* OR faculty) AND ("COVID-19" OR "SARS-CoV-2" OR coronavirus OR "long COVID" OR "post-acute COVID") AND ("executive function*" OR "cognitive control" OR "working memory" OR "cognitive inhibition" OR "cognitive flexibility" OR "cognitive performance") |
| SPORTDiscus | (teacher* OR professor* OR educator* OR instructor* OR tutor* OR lecturer* OR faculty) AND ("COVID-19" OR "SARS-CoV-2" OR coronavirus OR "long COVID" OR "post-acute COVID") AND ("executive function*" OR "cognitive control" OR "working memory" OR "cognitive inhibition" OR "cognitive flexibility" OR "cognitive performance") |
| LILACS | (teacher* OR professor* OR educator* OR instructor* OR tutor* OR lecturer* OR faculty) AND ("COVID-19" OR "SARS-CoV-2" OR coronavirus OR "long COVID" OR "post-acute COVID") AND ("executive function*" OR "cognitive control" OR "working memory" OR "cognitive inhibition" OR "cognitive flexibility" OR "cognitive performance") |

**Reviewer #5:**

Dear authors,
Thank you for this revised version for partially addressing my comments. I focus my review this time on the search strategies. There are some mistakes, and all search strategies need revising by a medical librarian or seek any international service (e.g., systematicreviews.org). The MeSH term "teachers" does not exist. The MeSH term"COVID-19" is not enough to capture all potentially relevant evidence (e.g., "Post-Acute COVID-19 Syndrome"[Mesh] OR "SARS-CoV-2"[Mesh]). What about "Cognition Disorders"[Mesh]?. The table 1 in page 7 has not changed from previous version. In the asbtract, you state where you search PubMed interface. for Medline only or all for non-Medline. Or you search Medline in another platform as Medline Ovid. All these terms are not similar, and it is important for reproducibility issues.

**Authors’ Response:** Thank you for this important methodological observation. We have revised all search strategies to ensure correct use of controlled vocabulary and improved sensitivity. Specifically, we replaced the non-existent MeSH term “teachers” with the valid terms “School Teachers” and “Faculty”; expanded the COVID-19 block to include “SARS-CoV-2” and “Post-Acute COVID-19 Syndrome”; and refined the cognition block by incorporating additional relevant MeSH terms. We also clarified that MEDLINE will be searched via the PubMed interface to ensure reproducibility. The updated, fully detailed strategies are now presented in the appendix. Please, see as follow:

| Database | Search strategy |
| --- | --- |
| MEDLINE (via PubMed) | ("School Teachers"[Mesh] OR "Faculty"[Mesh] OR teacher*[tiab] OR professor*[tiab] OR educator*[tiab]  OR instructor*[tiab] OR tutor*[tiab] OR lecturer*[tiab] OR faculty[tiab]) AND ("COVID-19"[Mesh] OR "SARS-CoV-2"[Mesh] OR "Post-Acute COVID-19 Syndrome"[Mesh] OR COVID-19[tiab] OR SARS-CoV-2[tiab] OR coronavirus[tiab] OR "long COVID"[tiab] OR "post-acute COVID"[tiab]) AND ("Executive Function"[Mesh] OR "Cognition"[Mesh] OR "Neuropsychological Tests"[Mesh] OR "Cognition Disorders"[Mesh]OR "executive function*"[tiab] OR "cognitive control"[tiab] OR "working memory"[tiab] OR "cognitive inhibition"[tiab] OR "cognitive flexibility"[tiab] OR "cognitive performance"[tiab]) |
| EMBASE | ('school teacher'/exp OR 'faculty member'/exp OR teacher*:ti,ab OR professor*:ti,ab OR educator*:ti,ab OR instructor*:ti,ab OR tutor*:ti,ab OR lecturer*:ti,ab) AND('covid-19'/exp OR 'sars-related coronavirus 2'/exp OR 'post-acute covid-19 syndrome'/exp OR covid-19:ti,ab OR sars-cov-2:ti,ab OR coronavirus:ti,ab OR 'long covid':ti,ab) AND('executive function'/exp OR 'cognition'/exp OR 'neuropsychological test'/exp OR 'cognitive defect'/exp  OR 'working memory'/exp OR 'cognitive control':ti,ab OR 'cognitive flexibility':ti,ab OR 'cognitive inhibition':ti,ab OR 'cognitive performance':ti,ab) |
| Scopus | TITLE-ABS-KEY(teacher* OR professor* OR educator*  OR instructor* OR tutor* OR lecturer* OR faculty) AND TITLE-ABS-KEY("COVID-19" OR "SARS-CoV-2" OR coronavirus OR "long COVID" OR "post-acute COVID") AND TITLE-ABS-KEY("executive function*" OR "cognitive control" OR "working memory" OR "cognitive inhibition"  OR "cognitive flexibility"OR "cognitive performance") |
| Web of Science | TS=(teacher* OR professor* OR educator* OR instructor* OR tutor* OR lecturer* OR faculty) AND TS=("COVID-19" OR "SARS-CoV-2" OR coronavirus OR "long COVID" OR "post-acute COVID") AND TS=("executive function*" OR "cognitive control" OR "working memory" OR "cognitive inhibition"  OR "cognitive flexibility"OR "cognitive performance") |
| Cochrane Library | ([mh "School Teachers"] OR [mh "Faculty"] OR teacher*:ti,ab,kw OR professor*:ti,ab,kw OR educator*:ti,ab,kw OR instructor*:ti,ab,kw OR tutor*:ti,ab,kw OR lecturer*:ti,ab,kw) AND ([mh "COVID-19"] OR [mh "SARS-CoV-2"] OR [mh "Post-Acute COVID-19 Syndrome"] OR "COVID-19":ti,ab,kw OR "SARS-CoV-2":ti,ab,kw OR coronavirus:ti,ab,kw OR "long COVID":ti,ab,kw) AND ([mh "Executive Function"] OR [mh "Cognition"] OR [mh "Neuropsychological Tests"] OR "executive function*":ti,ab,kw OR "working memory":ti,ab,kw OR "cognitive control":ti,ab,kw  OR "cognitive flexibility":ti,ab,kw) |
| ERIC | (teacher* OR professor* OR educator* OR instructor* OR tutor* OR lecturer* OR faculty) AND ("COVID-19" OR "SARS-CoV-2" OR coronavirus OR "long COVID" OR "post-acute COVID") AND ("executive function*" OR "cognitive control" OR "working memory" OR "cognitive inhibition" OR "cognitive flexibility" OR "cognitive performance") |
| SPORTDiscus | (teacher* OR professor* OR educator* OR instructor* OR tutor* OR lecturer* OR faculty) AND ("COVID-19" OR "SARS-CoV-2" OR coronavirus OR "long COVID" OR "post-acute COVID") AND ("executive function*" OR "cognitive control"  OR "working memory" OR "cognitive inhibition" OR "cognitive flexibility" OR "cognitive performance") |
| LILACS | (teacher* OR professor* OR educator* OR instructor* OR tutor* OR lecturer* OR faculty) AND ("COVID-19" OR "SARS-CoV-2" OR coronavirus OR "long COVID" OR "post-acute COVID") AND ("executive function*" OR "cognitive control" OR "working memory" OR "cognitive inhibition" OR "cognitive flexibility" OR "cognitive performance") |
