# Supplementary material for: Consequences of COVID-19 infection on educators’ executive functions: A systematic review and meta-analysis protocol
Source: PLoS One. 2026 Oct 14;21(10):e0350156. doi: 10.1371/journal.pone.0350156 (PMC13667799; doi:10.1371/journal.pone.0350156)
Supplement: Supplementary file 7 [file pone.0350156.s008.docx]

Dear reviewer, we are very grateful for your careful analysis and valuable comments. We use all of them to improve the methodological quality and scientific rigor of our work. Please see the answers to the questions below.

1- Title: The title is representative of the study design.

**Authors response:** Thanks for your positive feedback.

2- Abstract: The abstract is relatively acceptable in terms of overall structure and content, but there is a fundamental flaw in how the objective is stated. The objective of a protocol should not be "to conduct a systematic review" (explore the effects), but rather to describe the methodology and study criteria; this flaw in the abstract needs to be corrected.

**Authors response:** Thank you for this comment. We have revised the objective in the abstract to reflect the purpose of a protocol, emphasizing the methodological framework and predefined criteria for the planned systematic review and meta-analysis. See as follow:

“Objective: This study aims to present the methodological framework and predefined criteria that will guide a systematic review and meta-analysis investigating the effects of COVID-19 infection on educators’ executive functions.

3- Introduction: The introduction section is acceptable in terms of overall coverage of the topic, but it has weaknesses in conceptual precision, clarity of the research gap. The text uses terms such as "educational educators," "teachers," and "professors" but does not provide an operational or clear definition of this population (e.g., educational level, type of employment, gender, work experience). This ambiguity will cause problems later in the study's eligibility criteria.

**Authors response:** Thank you for this valuable comment. We intentionally adopted broad eligibility criteria for the educator population because of the limited number of studies available on this topic. Restricting participants by educational level, employment type, gender, or work experience could substantially reduce the available evidence. Therefore, our aim is to comprehensively map the existing literature across all educator groups. If sufficient data are identified, subgroup analyses based on these characteristics will be considered in the systematic review.

The introduction states that there are three core executive functions: inhibitory control, working memory, and cognitive flexibility. However, when elaborating on each, it only provides examples for working memory and cognitive flexibility, giving only a brief explanation for inhibitory control. Additionally, it mentions "planning" and "decision-making" as more complex skills but does not explain their relationship with the three core functions.

**Authors response:** Thank you for this comment. We have revised the description of executive functions by providing a clearer definition of inhibitory control and clarifying that higher-order abilities, such as planning and decision-making, arise from the interaction of the three core executive functions rather than constituting independent executive functions. See as follow:

“The literature has identified three central EFs (26): i) inhibitory control (i.e., the ability to suppress automatic or inappropriate responses/behaviors, resist distractions, and maintain goal-directed attention (17, 18) and maintaining selective attention (26, 27)), ii) working memory (i.e., retention and manipulation of information) (28), and ii) cognitive flexibility (i.e., adaptation to new rules (18) and perspectives (10). From these core functions, more complex skills such as planning, decision-making, problem-solving, and reasoning emerge (18). These higher-order abilities rely on the coordinated interaction of inhibitory control, working memory, and cognitive flexibility rather than representing independent executive functions.

There is no clarity in distinguishing between the direct effects of the virus and the indirect effects of the pandemic. The introduction discusses both neuronal damage caused by the virus (inflammation, hypoxia) and the stress, anxiety, and workload resulting from the pandemic, but it does not differentiate whether the impairment in teachers' executive functions is due to the infection itself or the psychosocial conditions of the pandemic. This confounding is a fundamental weakness in the study's design. The research gap is not explicitly stated.

**Authors response:** Thank you for this important comment. We acknowledge that distinguishing the direct neurological effects of SARS-CoV-2 infection from the indirect psychosocial consequences of the pandemic is currently a limitation of the available evidence rather than of the study design. We have clarified this point in the Introduction, emphasizing that the proposed systematic review aims to synthesize the existing evidence and identify knowledge gaps that may guide future research to better disentangle these mechanisms.

Although at the end it says "no systematic review has addressed this issue," it does not explain why, despite previous reviews on the general population, a specific review focused on teachers is needed. The differences between teachers and other professions (e.g., unique cognitive load, continuous interaction with students, high flexibility requirements) are not mentioned. There is inconsistency between the objectives and the research question.

**Authors response:** Thank you for this comment. The Introduction already includes a paragraph highlighting the importance of executive functions for educators, emphasizing the specific cognitive demands of teaching, such as multitasking, continuous information integration, responsiveness to dynamic classroom environments, and pedagogical decision-making. We have revised this section to further clarify that this rationale supports the need for a review specifically focused on educators. Please, see as follow:

“For educators, these EFs are critical to the performance of professional duties. Teaching demands the integration of information, multitasking, responsiveness to dynamic classroom environments (including online formats), and the ability to make pedagogical decisions under time constraints. Cognitive impairments in these areas may, therefore, directly compromise educational quality and teacher well-being. A report explained that COVID-19 led to impairments in executive function in 36% of cases (32). Other studies have shown similar results, indicating that COVID-19 infection may lead to disorders related to headache, confusion and attention difficulties (21, 23).”

The research question is: "Does COVID-19 affect teachers' executive functions in the long term?" But the objective of the protocol is: "to describe the methodology... to investigate the consequences of infection" (not to answer the question). This confusion between the protocol and the study itself is unacceptable.

**Authors response:** Thank you for this comment. We agree that the central research question is whether COVID-19 affects educators' executive functions in the long term. However, as this manuscript is a systematic review protocol, its objective is to describe the methodological procedures and predefined criteria that will guide the planned systematic review addressing this research question. We have revised the text to make this distinction clearer.

Furthermore, the term "long term" is not defined in the introduction (how many months? which indicator?).

**Authors response:** Thank you for this comment. We acknowledge that there is currently no consensus in the literature regarding a specific time threshold for defining long-term executive function outcomes following COVID-19 infection, particularly in educators. Therefore, we did not establish a predefined cutoff. Instead, the systematic review will synthesize the available evidence according to the follow-up periods reported in the included studies, allowing the findings to be presented and interpreted across different time intervals.

The authors mention four previous reviews (35-38) but do not critique any of them. For example, the methodological flaws of those reviews or why their results are not generalizable to teachers are not mentioned. The explanation of neural mechanisms (demyelination, inflammation) is repeated across several paragraphs. Some sentences, such as "These skills are important for teaching activities," appear more than once with similar phrasing.

**Authors response:** Thank you for this comment. We consider it appropriate to avoid generalizing findings from other occupational groups, as teaching involves specific cognitive demands that may influence executive functioning differently. Moreover, evidence on this topic remains limited, even in broader populations. We have clarified this rationale in the Introduction and revised the text to reduce repetitive descriptions of neural mechanisms and executive functions. We believe that the planned systematic review will help identify current evidence gaps and provide valuable information to guide future research and inform educational systems.

 Important confounding variables are not mentioned. Factors such as infection severity (hospitalized or outpatient), vaccination, history of neurological diseases, age, gender, and occupational stress level, which could modify the effect of COVID-19 on executive functions, are overlooked in the introduction. The writing requires editing.

**Authors response:** Thank you for this valuable comment. We agree that these variables are important and may influence the relationship between COVID-19 infection and executive function outcomes. Accordingly, we have incorporated a paragraph in the Introduction acknowledging these potential confounding factors and highlighting their relevance when interpreting the available evidence. Please, see as follow:

“Multiple factors may contribute to the variability in executive function outcomes following COVID-19 infection, including infection severity, vaccination status, pre-existing neurological conditions, age, sex, and occupational stress. These variables may contribute to the heterogeneity of cognitive outcomes reported across studies and should be considered when interpreting the available evidence (39, 40). Therefore, the proposed systematic review will also seek to identify and summarize these characteristics whenever they are reported in the included studies.”

4- Methods: The Methods section is generally well‑designed, with just a few minor issues that should be addressed. The exclusion criterion that removes individuals diagnosed with psychiatric illnesses introduces selection bias, because COVID-19 itself can cause psychiatric disorders such as depression, anxiety, and PTSD; excluding these individuals means only a subgroup of infected persons without psychiatric symptoms is included, which biases the estimated effect of COVID‑19 on executive functions, and these characteristics would be better treated as moderating variables rather than exclusion criteria. Similarly, the exclusion of studies that rely on self‑reported executive functioning creates a high risk of an empty review, since many pandemic‑era studies have used self‑report questionnaires like the BRIEF‑A or simple surveys;

**Authors response:** Thank you for this valuable comment. We acknowledge the reviewer's concern; however, these exclusion criteria were intentionally established to minimize potential confounding factors and improve the specificity of the review. Psychiatric disorders may independently affect executive function performance, making it difficult to isolate the association with COVID-19 infection. In addition, our objective is to synthesize evidence based on objective assessments of executive functions using validated neuropsychological instruments, such as the Five Digits Test (FDT), the Montreal Cognitive Assessment (MoCA), and the Rey Auditory Verbal Learning Test (RAVLT), rather than self-reported measures, which assess perceived cognitive functioning and are more susceptible to reporting bias. We have clarified this rationale in the manuscript.

If only studies employing objective neuropsychological tests (e.g., Wisconsin Card Sorting Test, Stroop, N‑back) are accepted, it is very likely that no eligible studies will be found. Furthermore, no operational definition of "educator" is provided in the eligibility criteria: the population is defined only as "adults working as teachers/educators at any educational level," with no criteria to distinguish educational level (preschool, primary, secondary, university), type of employment (full‑time, part‑time), or minimum work experience, leading to high population heterogeneity that will be difficult to manage in a quantitative synthesis.

**Authors response:** Thank you for this valuable comment. We appreciate the reviewer's concern; however, we have decided to maintain the inclusion of studies based on objective neuropsychological assessments. Although this approach may reduce the number of eligible studies, we believe it provides greater methodological rigor for evaluating executive functions. If no eligible studies are identified, this finding will itself highlight an important gap in the literature and reinforce the need for further research using objective cognitive assessments in educators. Regarding the definition of educators, we intentionally adopted broad eligibility criteria to maximize the identification of relevant studies in this emerging field. If sufficient data are available, participant characteristics, such as educational level, employment conditions, and workload, will be explored through subgroup or stratified analyses.

The protocol also states a threshold of 70% for low risk of bias without citing any reference or methodological justification for this cutoff.

**Authors response:** Thank you for alerting us to the lack of a reference for this information. The reference has been added. See below:

“Studies scoring 70% or higher will be considered to have a low risk of bias (58).”

58.	Noll M, de Mendonca CR, de Souza Rosa LP, Silveira EA. Determinants of eating patterns and nutrient intake among adolescent athletes: a systematic review. Nutr J. 2017 Jul 28;16(1):46.

In the text, references are made to Table 1 and Table 2, but Table 3 appears without a citation after the description of qualitative synthesis.

**Authors response:** Thank you for alerting us to the missing reference to Table 3 in the text. We have made the correction. See below:

“Descriptive and content analyses of the results will also be conducted based on the impact of COVID-19 on educators’ executive functions. The extracted data will include quantitative indicators such as means, standard deviations, and effect sizes related to executive function performance (e.g., working memory, cognitive flexibility, inhibitory control), as well as qualitative themes derived from narrative reports or interviews (Tab. 3).”

Finally, although the research question and introduction use the term "long‑term," the methods section does not define a minimum follow‑up period (e.g., 3 months, 6 months, 1 year), so studies with different follow‑up durations will be included without stratification.

**Authors response:** Thank you for this valuable comment. We acknowledge that no universally accepted definition of a minimum follow-up period for long-term executive function outcomes after COVID-19 infection currently exists, particularly in studies involving educators. Therefore, we did not establish a predefined cutoff. Instead, follow-up duration will be extracted from each included study, and the findings will be stratified according to the reported follow-up periods whenever possible.

5- Results: Not Applicable.

6- Tables: Tables present the key information in a structured format.

7- Figures: The PRISMA figure was appropriate in terms of standards.

8- Discussion and Limitation: The Discussion section is not appropriate for a systematic review protocol, as it discusses expected findings rather than methodological limitations. In a systematic review protocol, before the search and data analysis are carried out, a "Discussion" section does not conceptually make sense. Protocols typically use a "Discussion" or "Considerations" section to address the methodological challenges ahead, not to provide insights based on results. In this text, the Discussion is written as if findings already exist (e.g., "This protocol provides critical insights"). This is premature for a protocol and constitutes unscientific exaggeration.

**Authors response:** Thank you for this thoughtful comment. We respectfully prefer to retain the Discussion section, as its inclusion is consistent with the PRISMA-P recommendations for systematic review protocols. In addition, several recently published systematic review protocols have included a Discussion section with a similar purpose. We have revised this section to ensure that it focuses on the rationale, potential contributions, and anticipated methodological considerations of the proposed review rather than on the results of the review itself. Examples of recently published protocols are provided below for reference:

<https://link.springer.com/article/10.1186/2046-4053-4-1>

<https://bmjopen.bmj.com/content/15/5/e101478.abstract>

<https://bmjopen.bmj.com/content/16/5/e106406.abstract>

The Discussion contains unsubstantiated claims lacking supporting references, such as: "This protocol provides critical insights that can inform the development of interventions..." while no data yet exist.

**Authors response:** Thank you for this valuable comment. We agree with the reviewer's observation and have revised the sentence to avoid making unsupported claims about the protocol itself. The statement now refers to the potential contributions of the future systematic review rather than the protocol, as follows:

“By systematically examining these effects, the future review can provide critical insights that can inform the development of interventions to support educators’ mental health and enhance the quality of student teaching in post-pandemic times.”

Critical methodological limitations are not properly identified or analyzed. The limitations mentioned in the Discussion are very superficial and general: the limitation of "a limited number of studies" is noted, but its consequences (e.g., inability to perform meta‑analysis, lack of generalizability) are not addressed. For the limitation of "population heterogeneity," the proposed solution ("subgroup classification") is overly simplistic, and real statistical challenges (e.g., insufficient number of studies within subgroups) are ignored. Important limitations not mentioned in the Discussion include, for example, the high risk of an empty review due to restrictive eligibility criteria (excluding self‑report and psychiatric illness).

**Authors response:**Thank you for this valuable comment. We appreciate the reviewer's concern; however, we respectfully prefer to maintain the current text. Although our eligibility criteria may reduce the number of eligible studies, we do not consider the possibility of an empty review to be a methodological limitation. Rather, if no eligible studies are identified, this would represent an important finding by highlighting a significant gap in the literature and the need for future research employing objective neuropsychological assessments in educators. For this reason, we have chosen not to present this possibility as a limitation of the protocol.

Sentences such as "Executive functions are fundamental to an educator's professional performance" and "This field is still poorly explored" are repeated from the introduction. Instead of repeating, the Discussion should focus on the challenges facing the protocol.

**Authors response:** Thank you for this valuable comment. We agree that the Discussion should avoid repeating points already presented in the Introduction. Accordingly, we have revised this section to reduce redundancy and place greater emphasis on the methodological considerations and challenges of the proposed systematic review, as shown below.

A good Discussion in a protocol should honestly acknowledge the weaknesses of the study design, for example: excluding self‑report studies may lead to loss of real-world evidence; excluding individuals with psychiatric illness may introduce selection bias; the likely absence of a comparison group in many studies. The section on generalizability is very brief and unclear. It states: "Our findings will be limited to educators and cannot be generalized to all populations." However, it does not explain why educators differ from other populations (e.g., due to high cognitive load, occupational stress, or frequent interaction with students). This statement is unsupported and merely a general comment.

**Authors response:**Thank you for this valuable comment. We appreciate the reviewer's observation. The rationale for focusing specifically on educators and the unique cognitive demands of the teaching profession has already been addressed in the Introduction. Therefore, we prefer to avoid repeating these points in the Discussion and maintain the current structure to minimize redundancy across sections. See as follow:

“For educators, these EFs are critical to the performance of professional duties. Teaching demands the integration of information, multitasking, responsiveness to dynamic classroom environments (including online formats), and the ability to make pedagogical decisions under time constraints. Cognitive impairments in these areas may, therefore, directly compromise educational quality and teacher well-being. A report explained that COVID-19 led to impairments in executive function in 36% of cases (32). Other studies have shown similar results, indicating that COVID-19 infection may lead to disorders related to headache, confusion and attention difficulties (21, 23).”

9- Conclusion: The conclusion section contains claims about results that appear before the study has been conducted. For example, the statement "It is expected that the findings will help develop interventions..." is made even though no search, extraction, or analysis has yet been performed. In a protocol, the conclusion should be limited to methodology, not to future findings. The conclusion should briefly mention the main limitations (e.g., "This protocol may encounter a limited number of studies due to restrictive eligibility criteria"). In the current text, no limitations are mentioned.

**Authors response:** Thank you for this valuable comment. We agree that the conclusion of a systematic review protocol should focus on the methodological aspects of the study rather than anticipated findings. Accordingly, we have revised the Conclusion to emphasize the purpose of the protocol and its methodological framework, while also acknowledging the main potential limitations of the planned review, as shown below.

“This protocol establishes the methodological framework and predefined eligibility criteria for a systematic review and meta-analysis investigating the effects of COVID-19 infection on educators’ executive functions. By providing a transparent and reproducible methodology, this protocol aims to support the rigorous synthesis of the available evidence and identify current knowledge gaps on this topic. Potential limitations include the limited number of eligible studies and the heterogeneity of the available evidence, which may affect the scope of the planned quantitative synthesis.”
