# Supplementary material for: Consequences of COVID-19 infection on educators’ executive functions: A systematic review and meta-analysis protocol
Source: PLoS One. 2026 Oct 14;21(10):e0350156. doi: 10.1371/journal.pone.0350156 (PMC13667799; doi:10.1371/journal.pone.0350156)
Supplement: Peer review history

# Peer review: Decision Letter 0

**DOI:** 10.1371/journal.pone.0350156.r001

**Authors:** Jiawen Deng

9 May 2025

Dear Dr. Rezende,

Thank you for submitting your manuscript to PLOS ONE. After careful consideration, we feel that it has merit but does not fully meet PLOS ONE’s publication criteria as it currently stands. Therefore, we invite you to submit a revised version of the manuscript that addresses the points raised during the review process.

Please submit your revised manuscript by Jun 23 2025 11:59PM. If you will need more time than this to complete your revisions, please reply to this message or contact the journal office at plosone@plos.org. When you're ready to submit your revision, log on to https://www.editorialmanager.com/pone/ and select the 'Submissions Needing Revision' folder to locate your manuscript file.

- A rebuttal letter that responds to each point raised by the academic editor and reviewer(s). You should upload this letter as a separate file labeled 'Response to Reviewers'.
- A marked-up copy of your manuscript that highlights changes made to the original version. You should upload this as a separate file labeled 'Revised Manuscript with Track Changes'.
- An unmarked version of your revised paper without tracked changes. You should upload this as a separate file labeled 'Manuscript'.

If you would like to make changes to your financial disclosure, please include your updated statement in your cover letter. Guidelines for resubmitting your figure files are available below the reviewer comments at the end of this letter.

If applicable, we recommend that you deposit your laboratory protocols in protocols.io to enhance the reproducibility of your results. Protocols.io assigns your protocol its own identifier (DOI) so that it can be cited independently in the future. For instructions see: https://journals.plos.org/plosone/s/submission-guidelines#loc-laboratory-protocols. Additionally, PLOS ONE offers an option for publishing peer-reviewed Lab Protocol articles, which describe protocols hosted on protocols.io. Read more information on sharing protocols at https://plos.org/protocols?utm_medium=editorial-email&utm_source=authorletters&utm_campaign=protocols.

We look forward to receiving your revised manuscript.

Kind regards,

Jiawen Deng

Academic Editor

PLOS ONE

Journal Requirements:

When submitting your revision, we need you to address these additional requirements.

1. Please ensure that your manuscript meets PLOS ONE's style requirements, including those for file naming. The PLOS ONE style templates can be found at

https://journals.plos.org/plosone/s/file?id=wjVg/PLOSOne_formatting_sample_main_body.pdf and

https://journals.plos.org/plosone/s/file?id=ba62/PLOSOne_formatting_sample_title_authors_affiliations.pdf.

2. Please include a caption for figure 1.

[Note: HTML markup is below. Please do not edit.]

Reviewers' comments:

Reviewer's Responses to Questions

**Comments to the Author**

1. Does the manuscript provide a valid rationale for the proposed study, with clearly identified and justified research questions?

Reviewer #1: No

Reviewer #2: Yes

Reviewer #3: Yes

Reviewer #4: Yes

Reviewer #5: Partly

Reviewer #6: Yes

**********

2. Is the protocol technically sound and planned in a manner that will lead to a meaningful outcome and allow testing the stated hypotheses?

Reviewer #1: Partly

Reviewer #2: Yes

Reviewer #3: Yes

Reviewer #4: Yes

Reviewer #5: Partly

Reviewer #6: Partly

**********

3. Is the methodology feasible and described in sufficient detail to allow the work to be replicable?

Reviewer #1: No

Reviewer #2: Yes

Reviewer #3: Yes

Reviewer #4: Yes

Reviewer #5: No

Reviewer #6: Yes

**********

4. Have the authors described where all data underlying the findings will be made available when the study is complete?

The PLOS Data policy

Reviewer #1: Yes

Reviewer #2: Yes

Reviewer #3: No

Reviewer #4: Yes

Reviewer #5: Yes

Reviewer #6: No

**********

5. Is the manuscript presented in an intelligible fashion and written in standard English?

Reviewer #1: Yes

Reviewer #2: Yes

Reviewer #3: Yes

Reviewer #4: Yes

Reviewer #5: Yes

Reviewer #6: Yes

**********

Please use the space provided to explain your answers to the questions above and, if applicable, provide comments about issues authors must address before this protocol can be accepted for publication. You may also include additional comments for the author, including concerns about research or publication ethics.

You may also provide optional suggestions and comments to authors that they might find helpful in planning their study.

Reviewer #1: Thank you for submitting your manuscript for review. After a thorough evaluation, I believe that the article requires substantial improvements before it can be reconsidered for publication.

In particular, the introduction needs significant development. This section should not only present the topic but also provide a clear context, demonstrate its relevance within the field, and justify the study’s purpose. As it stands, the rationale appears underdeveloped, which gives the impression that the topic is being addressed arbitrarily—this can introduce bias into the overall approach of the work.

Additionally, the methodology section should be rewritten to ensure greater transparency. The procedures must be described in enough detail to allow for replication by other researchers. In this regard, I also recommend improving the eligibility criteria, which are currently too vague and insufficiently specific to support reproducibility.

You will find detailed comments in the attached document.

Reviewer #2: 1. A clearer operational definition of executive functions (e.g., which subdomains or assessments will be included).

2. Possibly adding a note about the scope limitation (i.e., generalizability to populations beyond professors) and whether any comparator analysis is anticipated in the future.

Reviewer #3: Dear author,

I read your protocol submitted to PLOSone titled: “Consequences of COVID-19 infection on professors’ executive functions: a systematic review protocol”

Below are my comments

General:

There is a large body of evidence supporting the notion of COVId-19 induced executive dysfunction, and there are many systematic reviews an meta-analyses published on the subject

You have identified a gap in the knowledge specifically around EFs in educators in the academic setup

You stated that the subjects of interest in the review are professors. It would be helpful to change the word or provide an explanation early in the text. A suggestion owould be to use the term senior educator or experienced educator. Are you specifically after those with Professor designation? Any limitation on academic background or speciality? One can assume that a Professor in Math might be affected differently than a professor in English literature for example.

Line 191: “ Additional sensitivity analyses will be conducted.” - Specify how and when

Reviewer #4: Great efforts have been done in this manuscript and highlighted a specific condition regarding professors. Very good protocol and all the best.

Reviewer #5: Dear authors,

Thank you for this opportunity to comment in your protocol.

I focus this time on the search strategies. The essential part of a systematic review to identify all potentially available evidence. In your protocol, you mentioned several databases. That is great. However, I was only able to check the search equations for PubMed. The current version does not align with a "good" search strategy that a medical librarian would do. I advise that you contact your library or have help from international services like systematicreviews.org. You are missing MeSH terms but also need targeting the important field codes (like title and abstract of article in PubMed, where the body text is not searchable). It is recommended to have all search strategies available for the other databases for peer review and reproducibility issues. The prisma flow chart does not seem to be the latest from Page MJ (2020) available at https://www.prisma-statement.org/prisma-2020.

Reviewer #6: PONE-D-25-03730

1. Data Availability: Please specify where the data is available and for how long. If it is subjected to some institutional policy, please mention that, too. Please elaborate more.

2. Abstract: The objective reads like that of a Qualitative study. It needs to be framed as a Review study's objective: "What is the body of literature on..." and so on..

Line 28: no need to mention 'Peers' , just reviewers would suffice.

Line 30: Does it depend on the results? Or would it depend on the review question? Please clarify this, whether your review would assume a narrative synthesis or meta-analysis, in that case, you may have to include a quality checking of the included articles based on their methods. Or in other words, please mention why a risk of bias analysis is needed for the review, in light of your review objectives, Add sources, for the method/framework you will apply.

3. Line 127-128: Does not mention content: Exposure: CoVID-19 infection in inclusion criteria. Or time frame and language articles published in. Please mention everything and also a tentative idea about the time frame of search if the authors have an idea of that in advance.

4. No details on how search results will be recorded before any of the systematic steps start. Mention how yielded results will be recorded (which format- CSV/.Ref etc. , whether any software will be used (i.e. Excel / Endnote/ others reference managing softwares), how long data will be stored for, wheterh raw data of the search will be shared/ available for the readers,

5. Data extraction, synthesis and analysis: Mention how many reviewers in total will undertake each step and break-down each of the steps! (title abstract and full-text screening / reviewing/ data extraction/ analysis-synthesis), mention if same reviewers will be conducting all the steps.

6. Table 2 comes before Figure 1, though appears otherwise in text.

7. The most critical point is probably the limitation of the review mentioned in the last paragraph (Line 253). If this is essentially the case (should the topic be so novel that the search results may not return any articles so as to appear as an ‘Empty Review’, then perhaps the authors should undertake a scoping review method which would serve the purpose better (provide an overview of literature on a relatively new topic, among pothers). In that case such elaborate risk-of-bias analysis plan do not have to be presented. This approach would also align with the idea that some preliminary search / in cases, a whole scoping review would take place before undertaking the actual Systematic Review. The authors may also consider broadening their search strategy/ operational definitions/ eligibility criteria to make sure some articles show up in search result!

I wish the authors best of luck in their decisions, publishing the protocol and finishing the review!

**********

what does this mean?). If published, this will include your full peer review and any attached files.

If you choose “no”, your identity will remain anonymous but your review may still be made public.

**Do you want your identity to be public for this peer review?**  For information about this choice, including consent withdrawal, please see our Privacy Policy

Reviewer #1: No

Reviewer #2: No

Reviewer #3: **Yes:**  Noora Alshahani

Reviewer #4: No

Reviewer #5: No

Reviewer #6: No

**********

[NOTE: If reviewer comments were submitted as an attachment file, they will be attached to this email and accessible via the submission site. Please log into your account, locate the manuscript record, and check for the action link "View Attachments". If this link does not appear, there are no attachment files.]

While revising your submission, please upload your figure files to the Preflight Analysis and Conversion Engine (PACE) digital diagnostic tool, https://pacev2.apexcovantage.com/. PACE helps ensure that figures meet PLOS requirements. To use PACE, you must first register as a user. Registration is free. Then, login and navigate to the UPLOAD tab, where you will find detailed instructions on how to use the tool. If you encounter any issues or have any questions when using PACE, please email PLOS at figures@plos.org. Please note that Supporting Information files do not need this step.

Attachment Submitted filename: PONE-D-25-03730.pdf

Attachment Submitted filename: Comments to authors_PONE-D-25-03730.docx

# Author comment: Author response to Decision Letter 1

**DOI:** 10.1371/journal.pone.0350156.r002

13 Jun 2025

Dear reviewers, we are very grateful for your careful analysis and valuable comments. We use all

of them to improve the methodological quality and scientific rigor of our work. Please see the

answers to the questions below. The revised sentences are in red color in the manuscript file.

Reviewer #1:

Thank you for submitting your manuscript for review. After a thorough evaluation, I believe that

the article requires substantial improvements before it can be reconsidered for publication.

In particular, the introduction needs significant development. This section should not only

present the topic but also provide a clear context, demonstrate its relevance within the field,

and justify the study’s purpose. As it stands, the rationale appears underdeveloped, which gives

the impression that the topic is being addressed arbitrarily—this can introduce bias into the

overall approach of the work.

Authors’ Response: We appreciate the insightful comment. In response, we have significantly

revised the introduction to provide a clearer and more robust context for the study. The updated

section now includes: (1) a more comprehensive background on the cognitive consequences of

COVID-19 infection, (2) a detailed explanation of executive functions and their importance in

academic professions, and; (3) a clearer justification for focusing specifically on professors as a

population of interest. Please see highlighted in the introduction section.

Additionally, the methodology section should be rewritten to ensure greater transparency. The

procedures must be described in enough detail to allow for replication by other researchers. In

this regard, I also recommend improving the eligibility criteria, which are currently too vague and

insufficiently specific to support reproducibility.

Authors’ Response: We appreciate the reviewer’s thoughtful feedback regarding the clarity and

transparency of our methodology. In response, we have thoroughly revised the Methods section

to ensure it provides sufficient detail to allow for replication by other researchers. Please, See

highlighted in the text, as follows:

Table 2. Eligibility criteria.

Inclusion Exclusion

Population Adult individuals (>18 years)

who work as a

teacher/educator at any

educational levels

individuals diagnosed with

psychiatric illnesses

Exposure COVID-19 infection - Not exposed to COVID-19

infection

Outcomes Analysis of the executive

functions of teachers who

were infected with COVID-19

Studies that used nonvalidated

methods to analyze

executive functions

Self-reported executive

functioning

Studies that analyzed

executive functions outside

the context of COVID-19

Study design Cross-sectional, longitudinal;

random and non-random

studies

Literature reviews; studies

with retractions”

You will find detailed comments in the attached document.

Authors’ Response: We appreciate the careful review. All points have been considered. The

point-by-point response follows.

Abstract

as I understand it is a protocol not a review so the focus should be on the protocol and not the

review itself.

Authors’ Response: Adjusted. Please see abstract, as follows:

“This systematic review protocol aims to identify, synthesize, and critically evaluate the

existing body of literature on the effects of COVID-19 on educator’s executive

functions.”

The elegibility criteria should be reported. Please review https://www.prismastatement.

org/abstracts

Authors’ Response: Adjusted. Please see abstract, as follows:

“Method: We will search seven databases—Web of Science Core Collection, Scopus,

MEDLINE/PubMed via the National Library of Medicine interface, Cochrane Library,

Latin American and Caribbean Health Sciences, Embase, ERIC, and SPORTDiscus via

the EBSCOhost interface. Data extraction and analysis will be performed according to

the guidelines of the Preferred Reporting Items for Systematic Reviews and Meta-

Analysis Protocols. The entire process will be carried out for two independent reviewers,

with any discrepancies resolved by a third reviewer. The eligibility criteria followed the

PECO strategy. To be included, articles will have to be: i) original published, ii) studies

with educators, and iii) studies that analyzed executive functions. Conversely, exclusion

criteria will be: i) duplicates, ii) literature reviews, and iii) studies with retractions. If the

search strategy yields a sufficient number of methodologically comparable studies, metaanalytic

statistical methods will be applied, including meta-analysis or meta-regression,

sensitivity, consistency, precision, and certainty of evidence analyses.”

I suggest not using the same words as in the title to generate more impact.

Authors’ Response: Adjusted, as follows:

“Key-words: Executive control; SARS-COV-2; public health; educators.“

Introduction:

1) I think that, since it is a protocol, the introduction should be much more in-depth than it is at

present. It is important that they not only discuss each concept but also explain some studies

and delve into existing results that demonstrate the relevance of studying the problem. If this is

not done, it may seem that the topic is chosen at random, which may generate biases.

2) Explaining this can be good if we talk about the effects of people who have suffered from

covid, but it is not so interesting for the question they have asked, I think you should check well

what they are looking to realize. Your theory is that all these effects affected executive functions

but they do not discuss other variables that also influence and seem to reduce the problem in a

very simple way. I recommend that they also discuss other related biopsychosocial aspects.

3) I think this definition is quite simple for what the executive functions entail, I suggest to go

deeper into it.

4) since executive functions have different authors and different definitions because of this, I

suggest commenting on this as well or, at least, pointing out why it is decided to follow this line

itself. I think it is necessary because the way it is written it seems that there is an absolute

consensus.

5) this should be deepened

6) In order to be able to say this, they must also explain what already exists. Are there any other

studies on similar topics?

7) I think this did not go into too much depth to be able to take this on.

Authors’ Response: We appreciate the insightful comments. we have taken your suggestion

and significantly modified the introduction to improve the text, as follows:

“The coronavirus disease (COVID-19) has led to significant challenges in the

world, affecting various aspects of daily life, including education (1). While much

attention has been given to the respiratory and systemic effects of the virus, increasing

evidence suggests that COVID-19 also affects the central nervous system (CNS) (2).

There are also reports of psychosocial issues, leading to disorders such as anxiety and

depression (3, 4). Therefore, we are aware that the extent of the virus infection and the

pandemic context experience have broad repercussions. This suggests the need for greater

in-depth analysis of the long-term consequences for the population.

Aiming to broaden understanding, a biopsychosocial approach is necessary. From

a biological perspective, COVID-19 has the potential to lead to neuroinflammation—

commonly referred to as a “cytokine storm” (5), hypoxia (6), vascular damage (7) and,

potentially leading to neuronal damage and cognitive deficits (8). The psychological

aspect is also affected. Studies point to the incidence of feelings of tiredness and stress

(9, 10), the “brain fog” (11) and mental confusion (12). The social consequences have

also been devastating. The pandemic has forced a routine break, distancing and reclusion,

and work dynamics changes, generating problems such as anxiety and family financial

crises, for example (13). Indeed, previous research has linked such impairments to a

decline in executive functions (EFs) (14-16). These effects can even contribute to

neuronal injury and cognitive deficits (17, 18).

The impact on the CNS occurs as SARS-CoV-2 is a neurotrophic virus, that is,

has attraction for nerve cells (19). The virus reaches the CNS through sensory and motor

pathways, for example, the olfactory nerve pathway. When it reaches the CNS, it triggers

demyelination of nerve fibers and inflammatory processes (19). These trigger a series of

neuropsychiatric disorders. In the short-term, complications such as cerebrovascular

events, headache, encephalopathies, anosmia, among others, are observed. (20-23). The

literature has already become aware of the long-term infection consequences. Symptoms

of inattention and dysexecutive dysfunction are common (24). There are also reports of

frontotemporal hypoperfusion, as well as structural brain abnormalities, mainly in the

thalamic and temporal regions (25). Demyelination also causes an information processing

reduction, directly affecting performance in tasks related to the use of central executive

functions (2).

Among the cognitive domains potentially affected, executive functions (EFs) have

emerged as a particular concern (7–9). These high-order cognitive processes, also known

as cognitive control, include abilities essential for regulating thoughts and behaviors, such

as maintaining attention, organizing tasks, problem-solving, and adapting to new

information. The literature has identified three central EFs (26): inhibitory control (i.e.,

managing inappropriate behaviors (17, 18) and maintaining selective attention (26, 27)),

working memory (i.e., retention and manipulation of information) (28), and cognitive

flexibility (i.e., adaptation to new rules (18) and perspectives (10). From these core

functions, more complex skills such as planning, decision-making, problem-solving, and

reasoning emerge (18). For the purposes of this review, executive functions will be

operationally defined as cognitive processes assessed through validated instruments or

structured tasks that explicitly measure at least one of the following domains: inhibitory

control, working memory, or cognitive flexibility (29-31). These skills are important for

teaching activities as they allow the establishment of relationships between ideas, the

integration of new information with existing knowledge, and the ability to remember

sequences of events.

For educators, these EFs are critical to the performance of professional duties.

Teaching demands the integration of information, multitasking, responsiveness to

dynamic classroom environments (including online formats), and the ability to make

pedagogical decisions under time constraints. Cognitive impairments in these areas may,

therefore, directly compromise educational quality and teacher well-being. A report

explained that COVID-19 led to impairments in executive function in 36% of cases (32).

Other studies have shown similar results, indicating that COVID-19 infection may lead

to disorders related to headache, confusion and attention difficulties (21, 23, 24, 32).

In addition to fear, COVID-19 has created unexpected and high work demands.

This overloading teachers and imposing a routine that has resulted in a poor well-being

(33). Studies show that this disruption in well-being affects cognitive abilities, resulting

in low scores in EFs analyses (33, 34).

Despite initial studies suggesting a connection, gathering more solid evidence on

whether COVID-19 affects educators’ EFs is important. To the best of our knowledge, no

systematic review has addressed this issue (35, 36). The guiding research question was

the following: “Does COVID-19 affect teachers' EFs in the long term?” Therefore, this

systematic review aims to explore the effects of COVID-19 on educators’ EFs. This

knowledge can enhance our scientific understanding of the topic because EFs represent

an essential element for teaching activities. Thus, this study could potentially contribute

to proposals to improve educators’ mental health after the pandemic, which could

indirectly impact the quality of teaching for students.”

Materials and Methods

Any type of teacher? The psychosocial variables between levels are very different, as well as their

tasks. Review the entire introduction to give context to this

Authors’ Response: Yes. This study was designed to cover all educator’s, from different levels and

subjects. We believe it is necessary, since we know little about the subject. Later, specifications

may be made. Logically, depending on the results obtained from the databases, these groupings

and analyses may be carried out.

Hasn't this preliminary search already been carried out?

Authors’ Response: Yes. However, few studies have been found.

update the date as it is a protocol, I suggest that you extend it to the publication of the protocol.

Authors’ Response: Adjusted. Please see in the text, as follows:

“The databases will be searched by August 2025 to identify potential articles for inclusion

in this systematic review.”

I am fine with the format in which you show it but I suggest you indicate each specific search

strategy as this allows for replicability, I suggest you review the table at

https://doi.org/10.1371/journal.pone.0296273.

Authors’ Response: Dear reviewer, we are grateful for the suggestion and have decided to

incorporate this table into our work (see Table 2). However, we believe that we should keep Table

1, since it shows us the search strategy used.

why was * not used as in the previous case?

Authors’ Response: The * is a truncation operator that allows you to recover variations of the

same word root. In this case, we believe that we have exhausted the possibilities and it would

not be necessary to use it.

it is repeated

Authors’ Response: It was removed from the table.

suggest pointing out in the text an example of the search number you found in this preliminary

adjustment

Authors’ Response: Dear author, we have chosen not to include this information. We believe that

it may seem that we are skipping steps and breaking the rules of the PRISMA manual. We are

working to soon publish these results in a systematic review format.

insert link after

Authors’ Response: Inserted. Please see text, as follows:

“Search results will be exported from the databases in RIS format, stored and organized

in Mendeley (reference manager), and subsequently imported into Rayyan software

(https://new.rayyan.ai/) for screening and selection.”

but in what way? In parallel, blinded...?

Authors’ Response: Inserted. Please see text

, as follows:

“The reviewers will carry out the analyses in parallel and using the "blind" feature.”

remember that this is the protocol, so you must detail everything very specifically and go very

well into each section of the methodology. This section should be improved.

Authors’ Response: The text has been adjusted. Please let me know what you think.

Reference

Authors’ Response: Reference inserted. See text, pleas

, as follows:

“The eligibility criteria for this study were determined based on population, exposure,

comparison, and outcome (PECO) (Tab. 2) (38, 39).

But in what way? just having gone through it or having been infected?

Authors’ Response: Adjusted. Please see in the text

, as follows:

“Accordingly, the following parameters are established: population (P), educators;

exposure (E), have been infected with COVID-19; and outcome (O), executive function.”

it is necessary to specify this very well, what type of teacher and what characteristics should be

excluded. In addition, they should define the characteristics very well because I think they could

be biased, not only for being a teacher influences the covid in one way but also the age for

example could also influence.

Authors’ Response: Our goal is not to deal specifically with areas or levels of education, but

rather to explore teaching activities in general. We believe that this broad mapping and data

collection is important. Later, we will consider narrowing the analyses.

either way?

Authors’ Response: No restrictions. We believe that we will find few studies, due to the

specificity of the sample. Therefore, we will analyze any type of work that has focused on the

executive functions of teachers.

It is very important not only to detail which one is not, but also which one is. Not all studies are

going to give us the same indications nor are they going to be equally useful in the subject, this

should be reviewed. I suggest that you use a better inclusion and exclusion table and go deeper

into each section.

Authors’ Response: Dear reviewer, the table has been inserted. Please see text, as follows:

Table 2. Eligibility criteria.

Inclusion Exclusion

Population Adult individuals (>18 years)

who work as a

teacher/educator at any

educational levels

- Children and adolescents.

- Non-teaching adults.

Exposure COVID-19 infection - Not exposed to COVID-19

infection

Outcomes Analysis of the executive

functions of teachers who

were infected with COVID-19

Studies that used nonvalidated

methods to analyze

executive functions

Self-reported executive

functioning

Studies that analyzed

executive functions outside

the context of COVID-19

Study design Cross-sectional, longitudinal;

random and non-random

studies

Literature reviews; studies

with retractions”

should explain the exact data and indices to be used

Authors’ Response: Adjusted. Please see in the text, as follows:

“Descriptive and content analyses of the results will also be conducted based on

the impact of COVID-19 on educators’ executive functions. The extracted data will

include quantitative indicators such as means, standard deviations, and effect sizes related

to executive function performance (e.g., working memory, cognitive flexibility, inhibitory

control), as well as qualitative themes derived from narrative reports or interviews.

Indices such as test scores, neuropsychological assessments, and self-reported measures

will be used to categorize and synthesize findings.”

reference

Authors’ Response: Inserted. Please see text, as follows:

“The WebPlotDigitizer will be used to collect the data available in the graphs (47).”

how many times for how long?

Authors’ Response: Adjusted. Please see in the text, as follows:

“If any data are missing from the primary studies, the authors will be contacted by email

up to three times over a period of four weeks. If no response is received after this

period, the study will be included in the review with the available data, and the missing

information will be acknowledged as a limitation.”

You included several studies, they should take into account tools for each type of study.

Authors’ Response: Adjusted. Please see in the text , as follows:

“In summary, studies with scores equal to or above 70% will be considered to have

a low risk of bias. The risk of bias will be evaluated by two independent reviewers. In

case of disagreement, a third reviewer will be consulted. Different validated tools can will

be applied according to the design of each included study: for observational studies, the

Joanna Briggs Institute (JBI) critical appraisal tools will be used; for randomized

controlled trials, the Cochrane Risk of Bias Tool (RoB 2.0); for qualitative studies, the

JBI Checklist for Qualitative Research; and for mixed-methods studies, the Mixed

Methods Appraisal Tool (MMAT).”

this was not mentioned in the title, it should be added.

Authors’ Response: Adjusted. Please see title, as follows:

“Consequences of COVID-19 infection on educators’ executive functions: a systematic

review and meta-analysis protocol”

the software, the moderators and the indexes to be taken into account were not explained. It

should be reviewed.

Authors’ Response: Adjusted. Please see in the text, as follows:

All main analyses will consider studies that have investigated the impact of

COVID-19 on the executive functions of the teaching public. Therefore, the main

outcomes focused on in our analysis are EFs. Importantly, these analyses will be

categorized according to the type of study, separating cross-sectional studies from cohort

studies if the search returns to both models. This organization ensures that the analyses

are adapted to the context and peculiarities of each study design.

The pooled effect sizes will be calculated together with their 95% confidence

intervals, using the R statistical software. In this way, the analyses will make it possible

to assess the effect of COVID-19 on educators’ EFs. Based on the results obtained in the

systematic review, subgroup meta-analyses will be performed to investigate the

variability and possible modifications in the effects between different profiles of

individuals within the sample. Variables such as age, sex, socioeconomic context,

professional experience, and teaching level will be considered as potential moderators.

Statistical heterogeneity among the studies will be assessed using the I² statistic

and the Chi-squared (Q) test. If applicable, publication bias will be examined through

funnel plots and Egger’s test.

with all the data?

Authors’ Response: Adjusted. Please see in the text

, as follows:

“Additional sensitivity analyses will be conducted using all available data

included in the meta-analysis, based on predefined eligibility criteria. These analyses will

consider only complete datasets for the outcomes of interest (executive functions),

ensuring consistency across comparisons. Sensitivity analyses will be performed after the

initial meta-analysis and whenever significant heterogeneity or risk of bias is detected.”

it is not explained with which software

Authors’ Response: Inserted. Please see text , as follows:

“The GRADE assessments will be conducted using the GRADEpro GDT software

(https://gradepro.org).”

it is a protocol, please review it

Authors’ Response: Adjusted. Please see in the text , as follows:

“This systematic review protocol aims to understand”.

it is not a review

Authors’ Response: Adjusted. Please see in the text

, as follows:

“By systematically examining these effects, this protocol provides critical insights that

can inform the development of interventions to support educators’ mental health and

enhance the quality of student teaching in post-pandemic times.”

it is not the review, this is not the aim of the protocol

Authors’ Response: Adjusted. Please see in the text

, as follows:

“This protocol aims to establish the appropriate methodological criteria for later

carrying out the systematic review and meta-analysis to understand the impact of a serious

global event, the COVID-19 pandemic, on educators’ EFs. The findings are expected to

contribute to the development of interventions aimed at promoting educators’ cognitive

health, particularly executive functions, which are closely related to planning, attention,

emotional regulation, and decision-making. By supporting these capacities, such

interventions may indirectly favor improvements in educators’ mental health and

professional well-being, potentially influencing the overall quality of teaching and the

educational environment.”

You are talking about executive functions, do you think this will be the case? I think this is a

statement that is too far removed from direct impact

Authors’ Response: Adjusted. Please see in the text

, as follows:

“The findings are expected to contribute to the development of interventions

aimed at promoting educators’ cognitive health, particularly executive functions, which

are closely related to planning, attention, emotional regulation, and decision-making. By

supporting these capacities, such interventions may indirectly favor improvements in

educators’ mental health and professional well-being, potentially influencing the overall

quality of teaching and the educational environment.”

Reviewer #2:

1. A clearer operational definition of executive functions (e.g., which subdomains or

assessments will be included).

Authors’ Response: Inserted. Please see the introduction text, as follows:

“Among the cognitive domains potentially affected, executive functions (EFs) have

emerged as a particular concern (7–9). These high-order cognitive processes, also known

as cognitive control, include abilities essential for regulating thoughts and behaviors, such

as maintaining attention, organizing tasks, problem-solving, and adapting to new

information. The literature has identified three central EFs (26): inhibitory control (i.e.,

managing inappropriate behaviors (17, 18) and maintaining selective attention (26, 27)),

working memory (i.e., retention and manipulation of information) (28), and cognitive

flexibility (i.e., adaptation to new rules (18) and perspectives (10). From these core

functions, more complex skills such as planning, decision-making, problem-solving, and

reasoning emerge (18). For the purposes of this review, executive functions will be

operationally defined as cognitive processes assessed through validated instruments or

structured tasks that explicitly measure at least one of the following domains: inhibitory

control, working memory, or cognitive flexibility (29-31).”

2. Possibly adding a note about the scope limitation (i.e., generalizability to populations beyond

professors) and whether any comparator analysis is anticipated in the future.

Authors’ Response: Inserted. Please see the discussion text (lines: 322-325), as follows:

“Therefore, understanding the impact of COVID-19 on these variables is highly valuable

for a class of workers who perform socially and economically relevant roles. Furthermore,

understanding the long-term impact of the virus on the EFs of education professionals

will provide the necessary support for creating intervention plans aimed at minimizing

consequences, as well as for preventing new COVID-19 infections and new pandemic

episodes.”

“It is worth noting that our findings will be limited to educators and cannot be generalized

to all populations. Subsequent studies should observe specific classes of educators and

other populations.”

Reviewer #3: Dear author,

I read your protocol submitted to PLOSone titled: “Consequences of COVID-19 infection on

professors’ executive functions: a systematic review protocol”

Below are my comments

General:

There is a large body of evidence supporting the notion of COVId-19 induced executive

dysfunction, and there are many systematic reviews an meta-analyses published on the subject

You have identified a gap in the knowledge specifically around EFs in educators in the academic

setup

Authors’ Response: Dear reviewer, thank you for your insightful comment. Indeed, while there

is a substantial body of evidence on COVID-19-induced executive dysfunction in general

populations, our systematic review protocol specifically targets the impact on executive

functions in educators within the academic setting. This focus is important because executive

functions are critical for teaching activities, and understanding their potential impairment in this

group may have important implications for educational processes and outcomes. Our goal is to

contribute knowledge that can inform targeted interventions to support educators’ cognitive

health and, consequently, the quality of education.

You stated that the subjects of interest in the review are professors. It would be helpful to change

the word or provide an explanation early in the text. A suggestion owould be to use the term

senior educator or experienced educator. Are you specifically after those with Professor

designation? Any limitation on academic background or speciality? One can assume that a

Professor in Math might be affected differently than a professor in English literature for example.

Authors’ Response: This study was designed to cover all educators and teachers (we modified

the text), from different levels and subjects. We believe it is necessary, since we know little about

the subject. Later, specifications may be made. Logically, depending on the results obtained from

the databases, these groupings and analyses may be carried out.

Line 191: “ Additional sensitivity analyses will be conducted.” - Specify how and when

Authors’ Response: Inserted. Please see the text (lines: 248-253), as follows:

“Additional sensitivity analyses will be conducted using all available data

included in the meta-analysis, based on predefined eligibility criteria. These analyses will

consider only complete datasets for the outcomes of interest (executive functions),

ensuring consistency across comparisons. Sensitivity analyses will be performed after the

initial meta-analysis and whenever significant heterogeneity or risk of bias is detected.”

Reviewer #4:

Great efforts have been done in this manuscript and highlighted a specific condition regarding

professors. Very good protocol and all the best.

R: Dear reviewer, We are immensely grateful for the review and encouragement provided.

Reviewer #5:

Dear authors,

Thank you for this opportunity to comment in your protocol.

I focus this time on the search strategies. The essential part of a systematic review to identify

all potentially available evidence. In your protocol, you mentioned several databases. That is

great. However, I was only able to check the search equations for PubMed. The current version

does not align with a "good" search strategy that a medical librarian would do. I advise that you

contact your library or have help from international services like systematicreviews.org. You are

missing MeSH terms but also need targeting the important field codes (like title and abstract of

article in PubMed, where the body text is not searchable). It is recommended to have all search

strategies available for the other databases for peer review and reproducibility issues. The

prisma flow chart does not seem to be the latest from Page MJ (2020) available at

https://www.prisma-statement.org/prisma-2020.

Authors’ Response: Thank for your valuable and constructive feedback regarding our protocol,

especially concerning the search strategies.

We fully agree with your observations. In the revised version of our manuscript, we are preparing

a comprehensive Supplementary Material that will include the complete and detailed search

strategies for all databases mentioned (including MeSH terms and appropriate field codes such

as [tiab] for PubMed). This aims to ensure transparency, reproducibility, and adherence to best

practices in systematic reviews.

Additionally, we will update the PRISMA flowchart according to the most recent 2020 version, as

recommended.

The search strategies used both MeSH terms (Medical Subject Headings) and free-text terms

(title and abstract) for each concept in the PICO framework. Below, we present the full search

string used in PubMed, including MeSH descriptors.

Supplementary material

Pubmed:

("Teachers"[MeSH Terms] OR teacher*[tiab] OR professor*[tiab] OR educator*[tiab] OR

instructor*[tiab] OR tutor*[tiab] OR lecturer*[tiab])

AND

("COVID-19"[MeSH Terms] OR "COVID-19"[tiab] OR coronavirus[tiab] OR "SARS-CoV-2"[tiab] OR

"long COVID"[tiab] OR omicron[tiab] OR delta[tiab] OR "severe acute respiratory

syndrome"[tiab])

AND

("Executive Function"[MeSH Terms] OR "Cognition"[MeSH Terms] OR “attention” OR "executive

function*"[tiab] OR "cognitive control"[tiab] OR "working memory"[tiab] OR "cognitive

inhibition"[tiab] OR "mental function*"[tiab] OR "frontal lobe function*"[tiab])

Scopus:

TITLE-ABS ( teacher* OR professor* OR educator* OR instructor* OR tutor* OR lecturer* )

AND

TITLE-ABS ( "COVID-19" OR coronavirus OR "SARS-CoV-2" OR "long COVID" OR omicron OR

delta OR "severe acute respiratory syndrome" )

AND

TITLE-ABS ( "executive function*" OR "cognitive control" OR "working memory" OR "cognitive

inhibition" OR "mental function*" OR "cognitive performance" OR "frontal lobe function*" )

Web of science:

TS=(teacher* OR professor* OR educator* OR instructor* OR tutor* OR lecturer*)

AND

TS=("COVID-19" OR coronavirus OR "SARS-CoV-2" OR "long COVID" OR omicron OR delta OR

"severe acute respiratory syndrome")

AND

TS=("executive function*" OR "cognitive control" OR "working memory" OR "cognitive

inhibition" OR "mental function*" OR "frontal lobe function*" OR "cognitive performance")

Cochrane Library:

(teacher* OR professor* OR educator* OR instructor* OR tutor* OR lecturer*):ti,ab,kw

AND

("COVID-19" OR coronavirus OR "SARS-CoV-2" OR "long COVID" OR omicron OR delta OR

"severe acute respiratory syndrome"):ti,ab,kw

AND

("executive function*" OR "cognitive control" OR "working memory" OR "cognitive inhibition"

OR "mental function*" OR "cognitive performance" OR "frontal lobe function*"):ti,ab,kw

Embase:

('teacher'/exp OR teacher*:ti,ab OR professor*:ti,ab OR educator*:ti,ab OR instructor*:ti,ab OR

tutor*:ti,ab OR lecturer*:ti,ab)

AND

('covid-19'/exp OR 'covid-19':ti,ab OR coronavirus:ti,ab OR 'sars-cov-2':ti,ab OR 'long

covid':ti,ab OR omicron:ti,ab OR delta:ti,ab OR 'severe acute respiratory syndrome':ti,ab)

AND

('executive function'/exp OR 'cognition'/exp OR 'executive function*':ti,ab OR 'working

memory':ti,ab OR 'cognitive performance':ti,ab OR 'cognitive inhibition':ti,ab OR 'mental

function*':ti,ab OR 'frontal lobe function*':ti,ab)

ERIC:

(teacher* OR professor* OR educator* OR instructor* OR tutor* OR lecturer*)

AND

("COVID-19" OR coronavirus OR "SARS-CoV-2" OR "long COVID" OR omicron OR delta OR

"severe acute respiratory syndrome")

AND

("executive function*" OR "cognitive control" OR "working memory" OR "cognitive inhibition"

OR "mental function*" OR "frontal lobe function*" OR "cognitive performance")

SPORTdiscuss

(teacher* OR professor* OR educator* OR instructor* OR tutor* OR lecturer*)

AND

("COVID-19" OR coronavirus OR "SARS-CoV-2" OR "long COVID" OR omicron OR delta OR

"severe acute respiratory syndrome")

AND

("executive function*" OR "cognitive control" OR "working memory" OR "cognitive inhibition"

OR "mental function*" OR "frontal lobe function*" OR "cognitive performance")

LILACS:

(teacher OR professor OR educator OR instructor OR tutor OR lecturer)

AND

("COVID-19" OR coronavirus OR "SARS-CoV-2" OR "long COVID" OR omicron OR delta OR

"síndrome respiratória aguda grave")

AND

("função executiva" OR "controle cognitivo" OR "memória de trabalho" OR "função mental" OR

"desempenho cognitivo" OR "inibição cognitiva")

Reviewer #6:

1. Data Availability: Please specify where the data is available and for how long. If it is

subjected to some institutional policy, please mention that, too. Please elaborate more.

Authors’ Response: Thank you for your observation. The data from this systematic review will

be made publicly available through the PROSPERO registry. All relevant materials—including the

search strategy, data extraction form, and synthesis plans—will remain accessible on the

PROSPERO platform for an indefinite period, following its open-access and transparency policies.

No institutional restrictions apply to the availability of this information.

2. Abstract: The objective reads like that of a Qualitative study. It needs to be framed as a

Review study's objective: "What is the body of literature on..." and so on..

Authors’ Response: Thanks for your valuable suggestion. Adjusted into the manuscript, as

follows:

“This systematic review protocol aims to identify, synthesize, and critically evaluate the

existing body of literature on the effects of COVID-19 on educator’s executive functions.”

Line 28: no need to mention 'Peers' , just reviewers would suffice.

Authors’ Response: Thanks for your valuable suggestion. Adjusted into the manuscript , as

follows:

“Data extraction and analysis will be performed according to the guidelines of the

Preferred Reporting Items for Systematic Reviews and Meta-Analysis Protocols. The

entire process will be carried out for two independent reviewers, with any discrepancies

resolved by a third reviewer.”

Line 30: Does it depend on the results? Or would it depend on the review question? Please

clarify this, whether your review would assume a narrative synthesis or meta-analysis, in that

case, you may have to include a quality checking of the included articles based on their

methods. Or in other words, please mention why a risk of bias analysis is needed for the

review, in light of your review objectives, Add sources, for the method/framework you will

apply.

Authors’ Response: Dear reviewer, we appreciate your comment. Our concern is that we may

not find enough work to allow us to conduct a systematic review (or even no work at all). This

is because this is a very specific population. In any case, we have adjusted the text to make this

clear (line 34), as follows:

“. If the search strategy yields a sufficient number of methodologically comparable

studies” (…).

Line 127-128: Does not mention content: Exposure: CoVID-19 infection in inclusion criteria. Or

time frame and language articles published in. Please mention everything and also a tentative

idea about the time frame of search if the authors have an idea of that in advance.

Authors’ Response: Thanks for your valuable suggestion. Adjusted into the manuscript, as

follows:

“The eligibility criteria for this study were determined based on population,

exposure, comparison, and outcome (PECO) (Tab. 2) (38, 39). Accordingly, the following

parameters are established: population (P), educators; exposure (E), have been infected

with COVID-19; and outcome (O), executive function.

Table 2. Eligibility criteria.

Inclusion Exclusion

Population Adult individuals (>18 years)

who work as a

teacher/educator at any

educational levels

individuals diagnosed with

psychiatric illnesses

Exposure COVID-19 infection - Not exposed to COVID-19

infection

Outcomes Analysis of the executive

functions of teachers who

were infected with COVID-19

Studies that used nonvalidated

methods to analyze

executive functions

Self-reported executive

functioning

Studies that analyzed

executive functions outside

the context of COVID-19

Study design Cross-sectional, longitudinal;

random and non-random

studies

Literature reviews; studies

with retractions

This systematic review will include articles published after the onset of the COVID-19

pandemic (March 11, 2020).”

4. No details on how search results will be recorded before any of the systematic steps start.

Mention how yielded results will be recorded (which format- CSV/.Ref etc. , whether any

software will be used (i.e. Excel / Endnote/ others reference managing softwares), how long

data will be stored for, wheterh raw data of the search will be shared/ available for the readers,

Authors’ Response: Dear reviewer, thank you for your comment. The information has been

included in the methods, as follows:

“Search results will be exported from the databases in RIS format, stored and

organized in Mendeley (reference manager), and subsequently imported into Rayyan

software (https://new.rayyan.ai/) for screening and selection. The raw search data will be

made available upon request, and all records will be retained indefinitely, in accordance

with institutional data management policies . The reviewers will carry out the analyses in

parallel and using the "blind" feature. Subsequently, the data from the selected studies

will be extracted independently by each reviewer”.

5. Data extraction, synthesis and analysis: Mention how many reviewers in total will undertake

each step and break-down each of the steps! (title abstract and full-text screening / reviewing/

data extraction/ analysis-synthesis), mention if same reviewers will be conducting all the steps.

R: Dear reviewer, thank you for your comment. The information has been included in the

methods (lines: 185-208) , as follows:

“The study selection process will be conducted in three sequential phases: title

screening, abstract screening, and full-text screening. All phases will be performed

independently by two reviewers (LM and SM), and any disagreements will be resolved

through discussion and, when necessary, adjudicated by a third reviewer (PS).

In the first phase, study titles will be screened against the predefined eligibility

criteria. In the second phase, abstracts of the selected titles will be reviewed by the same

two independent reviewers using the same inclusion and exclusion criteria. In the third

phase, the full texts of potentially eligible studies will be retrieved and examined to

confirm final inclusion (46). All three screening stages will be conducted independently

and blindly between LM and SM.

A spreadsheet will be created to include all fields presented in Table 3 for data

extraction. Two independent reviewers will evaluate and extract the data (LM and SM).

Any discrepancies will be addressed by consensus with a third reviewer (PS). Data

synthesis and analysis will also be performed by LM and SM, under the methodological

supervision of PS. Depending on the volume and quality of the included studies, both

qualitative synthesis and, where appropriate, meta-analytic methods (e.g., meta-analysis

or meta-regression) will be applied. Quality appraisal and risk of bias assessments will be

essential to inform the statistical approach and interpret the findings in alignment with

the review’s objectives.

Descriptive and content analyses of the results will also be conducted based on

the impact of COVID-19 on educators’ executive functions. The extracted data will

include quantitative indicators such as means, standard deviations, and effect sizes related

to executive function performance (e.g., working memory, cognitive flexibility, inhibitory

control), as well as qualitative themes derived from narrative reports or interviews.

Indices such as test scores, neuropsychological assessments, and self-reported measures

will be used to categorize and synthesize findings”.

6. Table 2 comes before Figure 1, though appears otherwise in text.

Authors’ Response: Adjusted! Please see into the manuscript file.

7. The most critical point is probably the limitation of the review mentioned in the last

paragraph (Line 253). If this is essentially the case (should the topic be so novel that the search

results may not return any articles so as to appear as an ‘Empty Review’, then perhaps the

authors should undertake a scoping review method which would serve the purpose better

(provide an overview of literature on a relatively new topic, among pothers). In that case such

elaborate risk-of-bias analysis plan do not have to be presented. This approach would also align

with the idea that some preliminary search / in cases, a whole scoping review would take place

before undertaking the actual Systematic Review. The authors may also consider broadening

their search strategy/ operational definitions/ eligibility criteria to make sure some articles

show up in search result!

Authors’ Response: Thank you very much for this thoughtful comment. We truly appreciate the

concern regarding the possibility of an “empty review” and the suggestion to consider a scoping

review approach. However, we respectfully intend to maintain the systematic review design as

originally proposed. This decision is grounded in our broader research plan, which includes an

experimental field study focusing on the effects of COVID-19 on executive functions in university

professors.

In this context, even a limited or null result from the systematic review will be also of scientific

value. A potential “empty review” would highlight the existing research gap and serve as a strong

justification and foundation for the field investigation currently underway. Furthermore, we

understand the implications of such a result for evidence synthesis, and for that reason, we have

designed a rigorous protocol to ensure transparency and methodological soundness, including

risk of bias assessment and planned sensitivity analyses, should sufficient data be available.

We have also carefully considered our search strategy and eligibility criteria to balance specificity

with comprehensiveness, aiming to capture relevant studies without compromising the review’s

focus. We remain open to refining the search strategy to maximize retrieval without altering the

scope or nature of the review.

Once again, we appreciate your valuable input and the opportunity to clarify our approach.

I wish the authors best of luck in their decisions, publishing the protocol and finishing the

review!

Attachment Submitted filename: Reviewers response.pdf

# Peer review: Decision Letter 1

**DOI:** 10.1371/journal.pone.0350156.r003

**Authors:** Jiawen Deng

9 Jul 2025

Dear Dr. Rezende,

Thank you for submitting your manuscript to PLOS ONE. After careful consideration, we feel that it has merit but does not fully meet PLOS ONE’s publication criteria as it currently stands. Therefore, we invite you to submit a revised version of the manuscript that addresses the points raised during the review process.

Both reviewers have provided very detailed comments in the attached peer-review documents. Please carefully review and make edits as necessary. Please also ensure that your protocol aligns with the content of your PROSPERO registration, as this will be checked on submission of the revision.

Please submit your revised manuscript by Aug 23 2025 11:59PM. If you will need more time than this to complete your revisions, please reply to this message or contact the journal office at plosone@plos.org. When you're ready to submit your revision, log on to https://www.editorialmanager.com/pone/ and select the 'Submissions Needing Revision' folder to locate your manuscript file.

- A rebuttal letter that responds to each point raised by the academic editor and reviewer(s). You should upload this letter as a separate file labeled 'Response to Reviewers'.
- A marked-up copy of your manuscript that highlights changes made to the original version. You should upload this as a separate file labeled 'Revised Manuscript with Track Changes'.
- An unmarked version of your revised paper without tracked changes. You should upload this as a separate file labeled 'Manuscript'.

If you would like to make changes to your financial disclosure, please include your updated statement in your cover letter. Guidelines for resubmitting your figure files are available below the reviewer comments at the end of this letter.

If applicable, we recommend that you deposit your laboratory protocols in protocols.io to enhance the reproducibility of your results. Protocols.io assigns your protocol its own identifier (DOI) so that it can be cited independently in the future. For instructions see: https://journals.plos.org/plosone/s/submission-guidelines#loc-laboratory-protocols. Additionally, PLOS ONE offers an option for publishing peer-reviewed Lab Protocol articles, which describe protocols hosted on protocols.io. Read more information on sharing protocols at https://plos.org/protocols?utm_medium=editorial-email&utm_source=authorletters&utm_campaign=protocols.

We look forward to receiving your revised manuscript.

Kind regards,

Jiawen Deng

Academic Editor

PLOS ONE

[Note: HTML markup is below. Please do not edit.]

Reviewers' comments:

Reviewer's Responses to Questions

**Comments to the Author**

1. Does the manuscript provide a valid rationale for the proposed study, with clearly identified and justified research questions?

Reviewer #1: Yes

Reviewer #5: Yes

Reviewer #6: Yes

**********

2. Is the protocol technically sound and planned in a manner that will lead to a meaningful outcome and allow testing the stated hypotheses?

Reviewer #1: Partly

Reviewer #5: Yes

Reviewer #6: Partly

**********

3. Is the methodology feasible and described in sufficient detail to allow the work to be replicable?

Reviewer #1: No

Reviewer #5: Yes

Reviewer #6: No

**********

4. Have the authors described where all data underlying the findings will be made available when the study is complete?

The PLOS Data policy

Reviewer #1: Yes

Reviewer #5: Yes

Reviewer #6: Yes

**********

5. Is the manuscript presented in an intelligible fashion and written in standard English?

Reviewer #1: Yes

Reviewer #5: Yes

Reviewer #6: Yes

**********

Please use the space provided to explain your answers to the questions above and, if applicable, provide comments about issues authors must address before this protocol can be accepted for publication. You may also include additional comments for the author, including concerns about research or publication ethics.

You may also provide optional suggestions and comments to authors that they might find helpful in planning their study.

Reviewer #1: Thank you for the opportunity to review this protocol. I hope the above comments are helpful in refining and strengthening the manuscript prior to publication.

Keywords: It is recommended not to repeat keywords that are already in the title, in order to increase the paper's reach and discoverability.

Abstract: Since this is a protocol, it would be more appropriate to end the abstract with a clear conclusion rather than including a discussion, which is not relevant in this context.

Introduction:

Line 57 of the introduction, the phrase that says “including education” and then continues discussing neuroinflammation seems to lack meaning, I suggest reviewing the wording.

Line 63: The text refers to "three core executive functions" but then lists four; this inconsistency should be corrected.

Line 72: Before stating that the topic is important for educators, it would be better to first elaborate on why that is the case and then place the statement at the end of the introduction.

Line 73: The term “other studies” is used, but the same author previously cited is referenced again, which may be confusing.

The introduction should explicitly mention previous systematic reviews and meta-analyses that have addressed similar topics, and clearly explain what novelty this review adds.

Methods:

Lines 91–92: A preliminary search is mentioned, but no findings are reported. It would be useful to briefly state what was identified.

Line 100: Please clarify that the full systematic search will be conducted once the protocol is published.

Lines 104–107: Consider removing the explanation of Boolean operators “OR” and “AND,” as it is unnecessary for a scholarly audience.

Table 1:

It is suggested to include the exact search strategies used for each database (copy-pasted) to enhance reproducibility.

The use of the asterisk (*) appears inconsistent across terms; please clarify the rationale.

Line 116: Add the appropriate citation for the PRISMA-S guideline.

Line 117: Include a link to the Rayyan platform.

Line 118: Mention that references from other systematic reviews will also be screened.

Eligibility Criteria (Line 121):

This section should be specified much more clearly. I suggest using a table and reviewing inclusion and exclusion criteria for each PECO component, and adding an “S” for the type of study. It is very important to determine how executive functions will be evaluated, and what will be considered an educator or teacher (from any level?). The exposure must be clearly defined — will only longitudinal studies with educators who have been exposed be included? Or will studies that simply mention COVID also be considered?

Figures and Tables:

Line 144: Figure 1 is not visible; please ensure it is included properly.

Line 151: Table 2 is not mentioned in the main text; it should be properly referenced.

Risk of Bias Assessment (Line 161):

Add the appropriate heading for this section.

It is not possible to evaluate the proposed bias assessment tool without a clear statement on the study designs that will be included.

Data Transformation (Line 168):

This section is unclear. What data will be transformed exactly? What data will be extracted specifically for each of the sections mentioned in Table 2 (e.g., mean, SD…?) Please clarify.

Meta-Analysis (Line 173):

The planned statistical analyses should be described in greater detail and with more technical specificity.

Sensitivity Analysis (Line 186):

What exactly will be reported? Will every individual analysis be described? What thresholds will be used to judge quality? Further elaboration is needed.

Limitations (Line 254):

The text states that relevant studies may not be found, but earlier a preliminary search was said to have been conducted. This appears contradictory and should be clarified.

Reviewer #5: Dear Authors, thank you for this revised version. I have few comments. In a table, you mentioned type of eligible studies as "random and non-random", please modify to "randomized and non-randomized". However, for ethical reasons, I argue that there will be no randomized controlled trials on this topic. This might be said somewhere in the protocol.

Please revise the English langauge for your protocol: "carried out for" seems weird: "carried out by", is my suggestion. The statement that cite ref. 9.10,11 and 12, needs to be more clear and fluent. The website of Rayyan does not naturally contains the subdomain "news". Please also cite it as Mourad Ouzzani, Hossam Hammady, Zbys Fedorowicz, and Ahmed Elmagarmid. Rayyan — a web and mobile app for systematic reviews. Systematic Reviews (2016) 5:210, DOI: 10.1186/s13643-016-0384-4.

You replied to a reviewer by saying you are going to "narrowing the analyses": you meant subgroup analyses. You mentioned this later in detail but make sure that the reader gets such information well in advance. YOu may add the code field "kw" in Embase as :ab,ti,kw. You may also improve the syntax and vocabulary to cover all potentially availabel evidence.

Proofread your protocol and good luck.

Reviewer #6: Much care should be taken in detailing out the analysis plan, themes, sub-themes etc.

reporting guidelines are yet to be developed for empty reviews, so need to be cautious.

Discussion and conclusions must be evidence -based.

Thank you and good luck, all authors!

**********

what does this mean?). If published, this will include your full peer review and any attached files.

If you choose “no”, your identity will remain anonymous but your review may still be made public.

**Do you want your identity to be public for this peer review?**  For information about this choice, including consent withdrawal, please see our Privacy Policy

Reviewer #1: No

Reviewer #5: **Yes:**  Dr Muaamar Al-Gobari

Reviewer #6: No

**********

[NOTE: If reviewer comments were submitted as an attachment file, they will be attached to this email and accessible via the submission site. Please log into your account, locate the manuscript record, and check for the action link "View Attachments". If this link does not appear, there are no attachment files.]

While revising your submission, please upload your figure files to the Preflight Analysis and Conversion Engine (PACE) digital diagnostic tool, https://pacev2.apexcovantage.com/. PACE helps ensure that figures meet PLOS requirements. To use PACE, you must first register as a user. Registration is free. Then, login and navigate to the UPLOAD tab, where you will find detailed instructions on how to use the tool. If you encounter any issues or have any questions when using PACE, please email PLOS at figures@plos.org. Please note that Supporting Information files do not need this step.

# Author comment: Author response to Decision Letter 2

**DOI:** 10.1371/journal.pone.0350156.r004

8 Sep 2025

Dear Editor,

Thank you for your comments.

We have already responded to your requests. The file sent under the name "Manuscript" is clean and does not contain any changes.

Regarding the indication of funding, this was an error and the project does not have direct financial support for its development.

I hope you will consider our manuscript for evaluation.

Sincerely,

Attachment Submitted filename: Reviewers_response_auresp_2.pdf

# Peer review: Decision Letter 2

**DOI:** 10.1371/journal.pone.0350156.r005

**Authors:** Steve Zimmerman

23 Sep 2025

Dear Dr. Rezende,

Thank you for submitting your manuscript to PLOS ONE. After careful consideration, we feel that it has merit but does not fully meet PLOS ONE’s publication criteria as it currently stands. Therefore, we invite you to submit a revised version of the manuscript that addresses the points raised during the review process.

The manuscript has been re-evaluated by two reviewers, and their comments are available below.

Please submit your revised manuscript by Nov 07 2025 11:59PM. If you will need more time than this to complete your revisions, please reply to this message or contact the journal office at plosone@plos.org. When you're ready to submit your revision, log on to https://www.editorialmanager.com/pone/ and select the 'Submissions Needing Revision' folder to locate your manuscript file.

- A rebuttal letter that responds to each point raised by the academic editor and reviewer(s). You should upload this letter as a separate file labeled 'Response to Reviewers'.
- A marked-up copy of your manuscript that highlights changes made to the original version. You should upload this as a separate file labeled 'Revised Manuscript with Track Changes'.
- An unmarked version of your revised paper without tracked changes. You should upload this as a separate file labeled 'Manuscript'.

If applicable, we recommend that you deposit your laboratory protocols in protocols.io to enhance the reproducibility of your results. Protocols.io assigns your protocol its own identifier (DOI) so that it can be cited independently in the future. For instructions see: https://journals.plos.org/plosone/s/submission-guidelines#loc-laboratory-protocols. Additionally, PLOS ONE offers an option for publishing peer-reviewed Lab Protocol articles, which describe protocols hosted on protocols.io. Read more information on sharing protocols at https://plos.org/protocols?utm_medium=editorial-email&utm_source=authorletters&utm_campaign=protocols.

We look forward to receiving your revised manuscript.

Kind regards,

Steve Zimmerman, PhD

Senior Editor, PLOS One

Journal Requirements:

If the reviewer comments include a recommendation to cite specific previously published works, please review and evaluate these publications to determine whether they are relevant and should be cited. There is no requirement to cite these works unless the editor has indicated otherwise.

Please review your reference list to ensure that it is complete and correct. If you have cited papers that have been retracted, please include the rationale for doing so in the manuscript text, or remove these references and replace them with relevant current references. Any changes to the reference list should be mentioned in the rebuttal letter that accompanies your revised manuscript. If you need to cite a retracted article, indicate the article’s retracted status in the References list and also include a citation and full reference for the retraction notice.

Additional Editor Comments (if provided):

Reviewer #1:

Reviewer #5:

Reviewer #6:

[Note: HTML markup is below. Please do not edit.]

Reviewers' comments:

Reviewer's Responses to Questions

**Comments to the Author**

1. Does the manuscript provide a valid rationale for the proposed study, with clearly identified and justified research questions?

Reviewer #1: Yes

Reviewer #5: Partly

Reviewer #6: Yes

**********

2. Is the protocol technically sound and planned in a manner that will lead to a meaningful outcome and allow testing the stated hypotheses?

Reviewer #1: Yes

Reviewer #5: Partly

Reviewer #6: Yes

**********

3. Is the methodology feasible and described in sufficient detail to allow the work to be replicable?

Reviewer #1: No

Reviewer #5: No

Reviewer #6: Yes

**********

4. Have the authors described where all data underlying the findings will be made available when the study is complete?

The PLOS Data policy

Reviewer #1: No

Reviewer #5: Yes

Reviewer #6: Yes

**********

5. Is the manuscript presented in an intelligible fashion and written in standard English?

Reviewer #1: Yes

Reviewer #5: Yes

Reviewer #6: Yes

**********

Please use the space provided to explain your answers to the questions above and, if applicable, provide comments about issues authors must address before this protocol can be accepted for publication. You may also include additional comments for the author, including concerns about research or publication ethics.

You may also provide optional suggestions and comments to authors that they might find helpful in planning their study.

Reviewer #1: Thank you for addressing all my comments. I think the manuscript has improved considerably, but I still think that not all the comments have been fully resolved, perhaps due to some confusion. Here are the minor revisions that the study would need:

Comment 1: Objective: I believe that the objective, even if changed, still does not correspond to what is being done. As this study corresponds to a protocol, the objective should not focus on synthesizing and critically evaluating the existing evidence, as that will be the result of the future review. The objective should be to establish the methodological guidelines and criteria for carrying out this review. You can also point out that the objective of the future review will be to synthesize, etc.

Comment 2: Data and indices to be used: I appreciate the adjustments made, however, I believe that the description still needs to be more specific. It is not enough to state that means, standard deviations, effect sizes, or neuropsychological tests in general will be included; it is necessary to indicate more precisely: When will a relevant difference be considered to exist (e.g., statistical significance thresholds, minimum effect sizes, or clinical criteria), what specific estimators will be taken into account (95% CI, p-values, Cohen's d, OR, RR, etc.)... and all sources supporting this use must be referenced.

Comment 3: Risk of bias: The clarification is adequate, but references are needed to justify the choice of the 70% threshold for considering low risk of bias. This criterion should be supported by recognized literature or methodological guidelines. Similarly, for each of the tools mentioned (RoB 2.0, JBI, MMAT, etc.), it would be advisable to include the corresponding reference, so that the theoretical and methodological basis for their application is clear. This will provide transparency and facilitate the reproducibility of the assessment.

Comment 4: Main analyses, meta-analyses and sensitivity analysis: The changes introduced are appropriate and enrich the analysis section. However, everything detailed in this part must also be properly referenced with evidence to support each decision.

Comment 5: Exact date of the search: It is not enough to indicate only the month in which the search was conducted. It is essential to indicate the exact date (day, month, and year) on which each search will be performed in the databases, which I also suggest should be after the protocol has been accepted.

Comment 7: Search strategy: I believe my previous comment has not been fully understood. I agree with the format currently presented and understand that you want to keep the tables included, but what I am requesting is not the eligibility criteria (as incorporated in Table 2), but the exact step-by-step search strategy. To ensure replicability, you need to include the complete search strings as they were executed in each database. The example I wanted to point out was not the table in the cited article, but the table in the appendix to that work (https://doi.org/10.1371/journal.pone.0296273) , which details the specific search strategies. I therefore suggest keeping the tables you already have, but adding an additional table (or an appendix) that explicitly reproduces each search strategy used in the different databases.

Reviewer #5: Dear authors,

Thank you for this revised version for partially addressing my comments. I focus my review this time on the search strategies. There are some mistakes, and all search strategies need revising by a medical librarian or seek any international service (e.g., systematicreviews.org). The MeSH term "teachers" does not exist. The MeSH term"COVID-19" is not enough to capture all potentially relevant evidence (e.g., "Post-Acute COVID-19 Syndrome"[Mesh] OR "SARS-CoV-2"[Mesh]). What about "Cognition Disorders"[Mesh]?. The table 1 in page 7 has not changed from previous version. In the asbtract, you state where you search PubMed interface. for Medline only or all for non-Medline. Or you search Medline in another platform as Medline Ovid. All these terms are not similar, and it is important for reproducibility issues.

Reviewer #6: Congratulations on the revisions made to your manuscript and specially, working on the method section to bring it up to a standard, even for a topic where you may be unsure of many of the parameters. Good luck with the broader study, and the systematic review. Take care!

**********

what does this mean?). If published, this will include your full peer review and any attached files.

If you choose “no”, your identity will remain anonymous but your review may still be made public.

**Do you want your identity to be public for this peer review?**  For information about this choice, including consent withdrawal, please see our Privacy Policy

Reviewer #1: No

Reviewer #5: No

Reviewer #6: No

**********

[NOTE: If reviewer comments were submitted as an attachment file, they will be attached to this email and accessible via the submission site. Please log into your account, locate the manuscript record, and check for the action link "View Attachments". If this link does not appear, there are no attachment files.]

While revising your submission, please upload your figure files to the Preflight Analysis and Conversion Engine (PACE) digital diagnostic tool, https://pacev2.apexcovantage.com/. PACE helps ensure that figures meet PLOS requirements. To use PACE, you must first register as a user. Registration is free. Then, login and navigate to the UPLOAD tab, where you will find detailed instructions on how to use the tool. If you encounter any issues or have any questions when using PACE, please email PLOS at figures@plos.org

# Author comment: Author response to Decision Letter 3

**DOI:** 10.1371/journal.pone.0350156.r006

21 Jan 2026

Dear reviewers, we are very grateful for your careful analysis and valuable comments. We use all of them to improve the methodological quality and scientific rigor of our work. Please see the answers to the questions below.

Reviewer #1: Thank you for addressing all my comments. I think the manuscript has improved considerably, but I still think that not all the comments have been fully resolved, perhaps due to some confusion. Here are the minor revisions that the study would need:

Comment 1: Objective: I believe that the objective, even if changed, still does not correspond to what is being done. As this study corresponds to a protocol, the objective should not focus on synthesizing and critically evaluating the existing evidence, as that will be the result of the future review. The objective should be to establish the methodological guidelines and criteria for carrying out this review. You can also point out that the objective of the future review will be to synthesize, etc.

Authors’ Response: We agree with the reviewer’s comment and have revised the objective accordingly. The objective now focuses on establishing the methodological guidelines and criteria for conducting the systematic review, while clarifying that the synthesis and critical evaluation of the evidence will be addressed in the future review. Please see below:

“This systematic review protocol aims to establish the methodological guidelines and criteria for carrying out this review. The future review will be identify, synthesize, and critically evaluate the existing body of literature on the effects of COVID-19 on educator’s executive functions.”

Comment 2: Data and indices to be used: I appreciate the adjustments made, however, I believe that the description still needs to be more specific. It is not enough to state that means, standard deviations, effect sizes, or neuropsychological tests in general will be included; it is necessary to indicate more precisely: When will a relevant difference be considered to exist (e.g., statistical significance thresholds, minimum effect sizes, or clinical criteria), what specific estimators will be taken into account (95% CI, p-values, Cohen's d, OR, RR, etc.)... and all sources supporting this use must be referenced.

Authors’ Response: We agree with the reviewer’s comment and have revised this section to provide a more specific and detailed description, including explicit criteria for relevant differences, the statistical estimators to be considered, and the appropriate supporting references. Please see below:

Descriptive, content, and quantitative analyses will be conducted to synthesize the impact of COVID-19 infection on educators’ executive functions. Quantitative data extracted from the included studies will comprise means and standard deviations of executive function outcomes, as well as effect size estimates comparing educators with prior COVID-19 infection to non-infected or pre-infection baseline groups. Executive functions of interest will include, but will not be limited to, working memory, cognitive flexibility, and inhibitory control, as assessed through standardized neuropsychological tests or validated self-report instruments.

A relevant difference between groups will be considered to exist when statistical significance is reported at a threshold of p < 0.05 and/or when the magnitude of the effect reaches at least a small standardized effect size. For continuous outcomes, effect sizes will be expressed as standardized mean differences (Cohen’s d or Hedges’ g), interpreted according to conventional criteria (small ≥ 0.20, moderate ≥ 0.50, large ≥ 0.80) (Cohen, 1988; Borenstein et al., 2009). For dichotomous outcomes, effect measures such as odds ratios (OR) or risk ratios (RR), along with their corresponding 95% confidence intervals (95% CI), will be extracted when available. A difference will be considered statistically meaningful when the 95% CI does not cross the null value (0 for standardized mean differences and 1 for OR/RR).

When studies report multiple executive function measures, priority will be given to validated and widely used neuropsychological instruments (e.g., Stroop Test, Trail Making Test, Wisconsin Card Sorting Test, Digit Span, or equivalent standardized tools). If outcomes are presented graphically without numerical values, data will be extracted using WebPlotDigitizer software (52), following established recommendations for data recovery from figures.

Qualitative data, including narrative descriptions, interviews, or open-ended responses, will be analyzed thematically to identify perceived cognitive changes associated with COVID-19 infection. These qualitative findings will be used to contextualize and complement the quantitative results but will not be pooled statistically.

If relevant quantitative data are missing or incomplete, corresponding authors will be contacted by email up to three times over a four-week period. If no response is obtained, the study will be included using the available data, and the missing information will be explicitly reported as a limitation of the review.

To ensure reliability in the data extraction process, inter-rater agreement will be assessed by calculating the percentage of agreement and Cohen’s kappa coefficient. Cohen’s kappa values will be interpreted as follows: <0.20 poor agreement, 0.21–0.40 fair agreement, 0.41–0.60 moderate agreement, 0.61–0.80 substantial agreement, and >0.80 almost perfect agreement (53, 54).

Comment 3: Risk of bias: The clarification is adequate, but references are needed to justify the choice of the 70% threshold for considering low risk of bias. This criterion should be supported by recognized literature or methodological guidelines. Similarly, for each of the tools mentioned (RoB 2.0, JBI, MMAT, etc.), it would be advisable to include the corresponding reference, so that the theoretical and methodological basis for their application is clear. This will provide transparency and facilitate the reproducibility of the assessment.

Authors’ Response: We thank the reviewer for this valuable comment, which helped us improve the transparency and methodological rigor of the protocol.

The threshold of 70% used to classify studies as having low risk of bias was based on previously published systematic reviews that adopted similar criteria when summarizing methodological quality across multiple domains. Specifically, this cutoff was guided by the approach used by Noll et al. (2017), who classified studies as having low, moderate, or high risk of bias according to the proportion of criteria met across the selected appraisal tool. This strategy has been applied in health-related systematic reviews as a pragmatic and transparent way to synthesize overall methodological quality when domain-level judgments are required.

To address the reviewer’s concern, we have now explicitly cited Noll et al. (2017) in the manuscript to justify the adoption of the 70% threshold and clarified that this criterion is intended as a methodological decision to enhance comparability and reproducibility, rather than as a strict universal standard.

In addition, we have included the original and methodological references for each risk-of-bias and quality assessment tool employed in the review. Specifically, we now cite the Cochrane Risk of Bias 2.0 tool for randomized studies, the Joanna Briggs Institute (JBI) critical appraisal tools for observational designs, and the Mixed Methods Appraisal Tool (MMAT) for mixed-methods studies. These references provide the theoretical and methodological foundations for the application of each instrument and ensure transparency in the assessment process.

We believe that these additions strengthen the methodological justification of the risk-of-bias assessment and improve the reproducibility and clarity of the protocol.

Comment 4: Main analyses, meta-analyses and sensitivity analysis: The changes introduced are appropriate and enrich the analysis section. However, everything detailed in this part must also be properly referenced with evidence to support each decision.

Authors’ Response: We thank the reviewer for highlighting the importance of methodological justification for all analytical decisions. In response, we have now added appropriate references to support each component of the main analyses, meta-analyses, and sensitivity analyses.

Comment 5: Exact date of the search: It is not enough to indicate only the month in which the search was conducted. It is essential to indicate the exact date (day, month, and year) on which each search will be performed in the databases, which I also suggest should be after the protocol has been accepted.

Authors’ Response: We agree with the reviewer’s comment and have revised the manuscript to specify the exact dates (day, month, and year) on which each database search will be performed, clarifying that the searches will be conducted after acceptance of the protocol.

Comment 7: Search strategy: I believe my previous comment has not been fully understood. I agree with the format currently presented and understand that you want to keep the tables included, but what I am requesting is not the eligibility criteria (as incorporated in Table 2), but the exact step-by-step search strategy. To ensure replicability, you need to include the complete search strings as they were executed in each database. The example I wanted to point out was not the table in the cited article, but the table in the appendix to that work (https://doi.org/10.1371/journal.pone.0296273) , which details the specific search strategies. I therefore suggest keeping the tables you already have, but adding an additional table (or an appendix) that explicitly reproduces each search strategy used in the different databases.

Authors’ Response: We thank the reviewer for the clarification and for providing a concrete example, which helped us fully understand the request. We agree that transparency and reproducibility of the search strategy are essential, particularly in a protocol for a systematic review and meta-analysis. In response to this comment, we confirm that the complete, step-by-step search strategies, exactly as executed in each database (including all Boolean operators, field tags, and filters), have already been developed and are now provided as supplementary material. This additional table explicitly reproduces the full search strings used for each database, following the format exemplified in the appendix of the referenced PLOS ONE article.

We have retained the existing tables in the main manuscript, as they summarize the eligibility criteria and databases searched, but we have added a new supplementary appendix dedicated exclusively to the detailed search strategies. This ensures full replicability of the search process while maintaining the clarity and structure of the main text.

We believe that the inclusion of this supplementary material fully addresses the reviewer’s concern and strengthens the methodological transparency of the protocol.

Reviewer #5: Dear authors,

Thank you for this revised version for partially addressing my comments. I focus my review this time on the search strategies. There are some mistakes, and all search strategies need revising by a medical librarian or seek any international service (e.g., systematicreviews.org). The MeSH term "teachers" does not exist. The MeSH term"COVID-19" is not enough to capture all potentially relevant evidence (e.g., "Post-Acute COVID-19 Syndrome"[Mesh] OR "SARS-CoV-2"[Mesh]). What about "Cognition Disorders"[Mesh]?. The table 1 in page 7 has not changed from previous version. In the asbtract, you state where you search PubMed interface. for Medline only or all for non-Medline. Or you search Medline in another platform as Medline Ovid. All these terms are not similar, and it is important for reproducibility issues.

Authors’ Response: Thank you very much for your careful review and for the detailed comments regarding the search strategies. We appreciate the opportunity to improve this critical component of the protocol.

In response to your comments, we have fully revised and refined all search strategies across the databases. Specifically, we removed the use of non-existent or inappropriate controlled vocabulary (e.g., the MeSH term “Teachers”) and restructured the population component using well-defined free-text terms. The COVID-19 search component was expanded to include additional controlled vocabulary and free-text terms, such as “SARS-CoV-2” and “Post-Acute COVID-19 Syndrome,” in order to increase sensitivity and ensure comprehensive coverage of the literature. The cognitive domain was also strengthened by incorporating validated controlled terms (e.g., “Executive Function,” “Cognition,” and “Cognition Disorders”) and relevant free-text synonyms.

In addition, we standardized and clearly specified the database platforms and interfaces used (e.g., MEDLINE searched via the PubMed interface) to avoid ambiguity and to ensure full reproducibility of the search process. All revised, database-specific, step-by-step search strategies are now explicitly reported as supplementary material, exactly as executed in each database.

We believe that the inclusion of this supplementary material fully addresses the reviewer’s concern and strengthens the methodological transparency of the protocol.

Reviewer #6: Congratulations on the revisions made to your manuscript and specially, working on the method section to bring it up to a standard, even for a topic where you may be unsure of many of the parameters. Good luck with the broader study, and the systematic review. Take care!

Authors’ Response: Thank you very much for your kind words and encouraging feedback. We truly appreciate your recognition of the efforts made to strengthen the methodological rigor of the manuscript. Your comments were very helpful in improving the clarity and robustness of the protocol. We are grateful for your time and thoughtful review, and we will certainly take your suggestions into account as we move forward with the broader study and the systematic review. Thank you again, and best regards.

Attachment Submitted filename: Reviewers_response_auresp_3.pdf

# Peer review: Decision Letter 3

**DOI:** 10.1371/journal.pone.0350156.r007

**Authors:** Steve Zimmerman

19 Feb 2026

Dear Dr. Rezende,

Thank you for submitting your manuscript to PLOS ONE. After careful consideration, we feel that it has merit but does not fully meet PLOS ONE’s publication criteria as it currently stands. Therefore, we invite you to submit a revised version of the manuscript that addresses the points raised during the review process.

Reviewer #1: Thank you for addressing all my comments. I think the manuscript has improved considerably, but I still think that not all the comments have been fully resolved, perhaps due to some confusion. Here are the minor revisions that the study would need:

Comment 1: Objective: I believe that the objective, even if changed, still does not correspond to what is being done. As this study corresponds to a protocol, the objective should not focus on synthesizing and critically evaluating the existing evidence, as that will be the result of the future review. The objective should be to establish the methodological guidelines and criteria for carrying out this review. You can also point out that the objective of the future review will be to synthesize, etc.

Comment 2: Data and indices to be used: I appreciate the adjustments made, however, I believe that the description still needs to be more specific. It is not enough to state that means, standard deviations, effect sizes, or neuropsychological tests in general will be included; it is necessary to indicate more precisely: When will a relevant difference be considered to exist (e.g., statistical significance thresholds, minimum effect sizes, or clinical criteria), what specific estimators will be taken into account (95% CI, p-values, Cohen's d, OR, RR, etc.)... and all sources supporting this use must be referenced.

Comment 3: Risk of bias: The clarification is adequate, but references are needed to justify the choice of the 70% threshold for considering low risk of bias. This criterion should be supported by recognized literature or methodological guidelines. Similarly, for each of the tools mentioned (RoB 2.0, JBI, MMAT, etc.), it would be advisable to include the corresponding reference, so that the theoretical and methodological basis for their application is clear. This will provide transparency and facilitate the reproducibility of the assessment.

Comment 4: Main analyses, meta-analyses and sensitivity analysis: The changes introduced are appropriate and enrich the analysis section. However, everything detailed in this part must also be properly referenced with evidence to support each decision.

Comment 5: Exact date of the search: It is not enough to indicate only the month in which the search was conducted. It is essential to indicate the exact date (day, month, and year) on which each search will be performed in the databases, which I also suggest should be after the protocol has been accepted.

Comment 7: Search strategy: I believe my previous comment has not been fully understood. I agree with the format currently presented and understand that you want to keep the tables included, but what I am requesting is not the eligibility criteria (as incorporated in Table 2), but the exact step-by-step search strategy. To ensure replicability, you need to include the complete search strings as they were executed in each database. The example I wanted to point out was not the table in the cited article, but the table in the appendix to that work (https://doi.org/10.1371/journal.pone.0296273) , which details the specific search strategies. I therefore suggest keeping the tables you already have, but adding an additional table (or an appendix) that explicitly reproduces each search strategy used in the different databases.

Reviewer #5: Dear authors,

Thank you for this revised version for partially addressing my comments. I focus my review this time on the search strategies. There are some mistakes, and all search strategies need revising by a medical librarian or seek any international service (e.g., systematicreviews.org). The MeSH term "teachers" does not exist. The MeSH term"COVID-19" is not enough to capture all potentially relevant evidence (e.g., "Post-Acute COVID-19 Syndrome"[Mesh] OR "SARS-CoV-2"[Mesh]). What about "Cognition Disorders"[Mesh]?. The table 1 in page 7 has not changed from previous version. In the asbtract, you state where you search PubMed interface. for Medline only or all for non-Medline. Or you search Medline in another platform as Medline Ovid. All these terms are not similar, and it is important for reproducibility issues.

Reviewer #6: Congratulations on the revisions made to your manuscript and specially, working on the method section to bring it up to a standard, even for a topic where you may be unsure of many of the parameters. Good luck with the broader study, and the systematic review. Take care!

-->In addition to addressing the above comments, please could you also:

1) Provide greater clarity and specificity regarding the outcome of interest: Please provide the specific measures of executive function that you will include. Please see: https://www.cochrane.org/authors/handbooks-and-manuals/mecir-manual/standards-conduct-new-cochrane-intervention-reviews-c1-c75/developing-protocol-review-c1-c23/selecting-outcomes-be-addressed-studies-included-review-c14-c18

2) Please be more precise about the time factor. Your research question is “Does COVID-19 affect teachers' 130 EFs in the long term?” How, exactly, is "long term" defined?

Please submit your revised manuscript by Apr 04 2026 11:59PM. If you will need more time than this to complete your revisions, please reply to this message or contact the journal office at plosone@plos.org. When you're ready to submit your revision, log on to https://www.editorialmanager.com/pone/ and select the 'Submissions Needing Revision' folder to locate your manuscript file.

- A letter that responds to each point raised by the academic editor and reviewer(s). You should upload this letter as a separate file labeled 'Response to Reviewers'.
- A marked-up copy of your manuscript that highlights changes made to the original version. You should upload this as a separate file labeled 'Revised Manuscript with Track Changes'.
- An unmarked version of your revised paper without tracked changes. You should upload this as a separate file labeled 'Manuscript'.

If you would like to make changes to your financial disclosure, please include your updated statement in your cover letter. Guidelines for resubmitting your figure files are available below the reviewer comments at the end of this letter.

If applicable, we recommend that you deposit your laboratory protocols in protocols.io to enhance the reproducibility of your results. Protocols.io assigns your protocol its own identifier (DOI) so that it can be cited independently in the future. For instructions see: https://journals.plos.org/plosone/s/submission-guidelines#loc-laboratory-protocols. Additionally, PLOS ONE offers an option for publishing peer-reviewed Lab Protocol articles, which describe protocols hosted on protocols.io. Read more information on sharing protocols at https://plos.org/protocols?utm_medium=editorial-email&utm_source=authorletters&utm_campaign=protocols.

We look forward to receiving your revised manuscript.

Kind regards,

Steve Zimmerman, PhD

Senior Editor, PLOS One

Journal Requirements:

If the reviewer comments include a recommendation to cite specific previously published works, please review and evaluate these publications to determine whether they are relevant and should be cited. There is no requirement to cite these works unless the editor has indicated otherwise.

[Note: HTML markup is below. Please do not edit.]

Reviewers' comments:

Reviewer's Responses to Questions

**Comments to the Author**

1. Does the manuscript provide a valid rationale for the proposed study, with clearly identified and justified research questions?

Reviewer #5: Yes

**********

2. Is the protocol technically sound and planned in a manner that will lead to a meaningful outcome and allow testing the stated hypotheses?

Reviewer #5: Yes

**********

3. Is the methodology feasible and described in sufficient detail to allow the work to be replicable?

Reviewer #5: Yes

**********

4. Have the authors described where all data underlying the findings will be made available when the study is complete?

The PLOS Data policy

Reviewer #5: Yes

**********

5. Is the manuscript presented in an intelligible fashion and written in standard English?

Reviewer #5: Yes

**********

Please use the space provided to explain your answers to the questions above and, if applicable, provide comments about issues authors must address before this protocol can be accepted for publication. You may also include additional comments for the author, including concerns about research or publication ethics.

You may also provide optional suggestions and comments to authors that they might find helpful in planning their study.

Reviewer #5: Dear Authors,

I noted that all my suggestions have been addressed. Thank you for all this efforts and wish you the best in conducting this review.

**********

what does this mean?). If published, this will include your full peer review and any attached files.

If you choose “no”, your identity will remain anonymous but your review may still be made public.

**Do you want your identity to be public for this peer review?**  For information about this choice, including consent withdrawal, please see our Privacy Policy

Reviewer #5: No

**********

[NOTE: If reviewer comments were submitted as an attachment file, they will be attached to this email and accessible via the submission site. Please log into your account, locate the manuscript record, and check for the action link "View Attachments". If this link does not appear, there are no attachment files.]

To ensure your figures meet our technical requirements, please review our figure guidelines: https://journals.plos.org/plosone/s/figures

You may also use PLOS’s free figure tool, NAAS, to help you prepare publication quality figures: https://journals.plos.org/plosone/s/figures#loc-tools-for-figure-preparation.

NAAS will assess whether your figures meet our technical requirements by comparing each figure against our figure specifications.

# Author comment: Author response to Decision Letter 4

**DOI:** 10.1371/journal.pone.0350156.r008

8 Apr 2026

Reviewer #1: Thank you for addressing all my comments. I think the manuscript has improved considerably, but I still think that not all the comments have been fully resolved, perhaps due to some confusion. Here are the minor revisions that the study would need:

Comment 1: Objective: I believe that the objective, even if changed, still does not correspond to what is being done. As this study corresponds to a protocol, the objective should not focus on synthesizing and critically evaluating the existing evidence, as that will be the result of the future review. The objective should be to establish the methodological guidelines and criteria for carrying out this review. You can also point out that the objective of the future review will be to synthesize, etc.

Authors’ Response: Adjusted. Please see introduction, as follows:

“To the best of our knowledge, no systematic review has addressed this issue (39, 40). The guiding research question was the following: “Does COVID-19 affect teachers' EFs in the long term?” This knowledge can enhance our scientific understanding of the topic because EFs represent an essential element for teaching activities. Thus, The objective of this protocol is to describe the methodological procedures, eligibility criteria, search strategy, and analytical approach that will be used to conduct a systematic review and meta-analysis examining the consequences of COVID-19 infection on educators’ executive functions.”

Comment 2: Data and indices to be used: I appreciate the adjustments made, however, I believe that the description still needs to be more specific. It is not enough to state that means, standard deviations, effect sizes, or neuropsychological tests in general will be included; it is necessary to indicate more precisely: When will a relevant difference be considered to exist (e.g., statistical significance thresholds, minimum effect sizes, or clinical criteria), what specific estimators will be taken into account (95% CI, p-values, Cohen's d, OR, RR, etc.)... and all sources supporting this use must be referenced.

Comment 3: Risk of bias: The clarification is adequate, but references are needed to justify the choice of the 70% threshold for considering low risk of bias. This criterion should be supported by recognized literature or methodological guidelines. Similarly, for each of the tools mentioned (RoB 2.0, JBI, MMAT, etc.), it would be advisable to include the corresponding reference, so that the theoretical and methodological basis for their application is clear. This will provide transparency and facilitate the reproducibility of the assessment.

Authors’ Response: Thank you for thess suggestions. We have revised the section to specify the statistical thresholds for significance (p < 0.05), the interpretation criteria for effect sizes (Cohen’s benchmarks), and the exact estimators to be used (Hedges’ g, OR, RR, 95% CI). We also clarified the criteria for conducting subgroup analyses and meta-regression (k ≥ 10) and included appropriate methodological references. Please see the text, as follows:

“Main meta-analyses and/or meta-regressions

The primary outcome will be post-COVID-19 executive function performance in educators, assessed through validated neuropsychological tests (e.g., working memory, inhibitory control, cognitive flexibility measures). For continuous outcomes, effect sizes will be calculated as standardized mean differences (Hedges’ g) with 95% confidence intervals (95% CI). Statistical significance will be defined as p < 0.05 (two-tailed). Effect size magnitude will be interpreted according to Cohen’s criteria (0.20 = small, 0.50 = moderate, 0.80 = large) (57).

For dichotomous outcomes, pooled estimates will be expressed as odds ratios (OR) or risk ratios (RR) with 95% CI. When adjusted estimates are available, the most fully adjusted model will be extracted (57).

A random-effects model (DerSimonian and Laird estimator) will be used. Between-study heterogeneity will be quantified using I² (25% low, 50% moderate, 75% high) and Cochran’s Q test (p < 0.10 indicating significant heterogeneity) (58). Subgroup analyses and meta-regression will be conducted when k ≥ 10 studies are available per covariate (59).

All analyses will be conducted in R (metafor and meta packages). Methodological decisions are based on established meta-analytic guidelines (57).”

Comment 4: Exact date of the search: It is not enough to indicate only the month in which the search was conducted. It is essential to indicate the exact date (day, month, and year) on which each search will be performed in the databases, which I also suggest should be after the protocol has been accepted.

Authors’ Response: Adjusted. Thank you for this clarification. We have revised the manuscript to specify the exact date on which the database searches will be conducted (10 April 2026). We also clarified that the searches will be performed after protocol acceptance to ensure transparency and inclusion of the most recent evidence. Please, se as follow:

“The electronic database searches will be conducted on 10 April 2026, following acceptance of this protocol, to ensure methodological transparency and to capture the most up-to-date evidence available at the time of the review.”

Comment 7: Search strategy: I believe my previous comment has not been fully understood. I agree with the format currently presented and understand that you want to keep the tables included, but what I am requesting is not the eligibility criteria (as incorporated in Table 2), but the exact step-by-step search strategy. To ensure replicability, you need to include the complete search strings as they were executed in each database. The example I wanted to point out was not the table in the cited article, but the table in the appendix to that work (https://doi.org/10.1371/journal.pone.0296273) , which details the specific search strategies. I therefore suggest keeping the tables you already have, but adding an additional table (or an appendix) that explicitly reproduces each search strategy used in the different databases.

Authors’ Response: Thank you for the clarification. We have now added a separate appendix table that provides the complete, database-specific search strings exactly as executed in each source, ensuring full transparency and reproducibility. The original tables have been maintained, and the additional appendix explicitly details the step-by-step search strategies, as suggested. Please, see as follow:

Terms used in the search strategy according to each database.

Database Search strategy

Cochrane Library ([mh "School Teachers"] OR [mh "Faculty"] OR teacher*:ti,ab,kw OR professor*:ti,ab,kw OR educator*:ti,ab,kw OR instructor*:ti,ab,kw OR tutor*:ti,ab,kw OR lecturer*:ti,ab,kw) AND ([mh "COVID-19"] OR [mh "SARS-CoV-2"] OR [mh "Post-Acute COVID-19 Syndrome"] OR "COVID-19":ti,ab,kw OR "SARS-CoV-2":ti,ab,kw OR coronavirus:ti,ab,kw OR "long COVID":ti,ab,kw) AND ([mh "Executive Function"] OR [mh "Cognition"] OR [mh "Neuropsychological Tests"] OR "executive function*":ti,ab,kw OR "working memory":ti,ab,kw OR "cognitive control":ti,ab,kw OR "cognitive flexibility":ti,ab,kw)

Pubmed ("School Teachers"[Mesh] OR "Faculty"[Mesh] OR teacher*[tiab] OR professor*[tiab] OR educator*[tiab] OR instructor*[tiab] OR tutor*[tiab] OR lecturer*[tiab] OR faculty[tiab]) AND ("COVID-19"[Mesh] OR "SARS-CoV-2"[Mesh] OR "Post-Acute COVID-19 Syndrome"[Mesh] OR COVID-19[tiab] OR SARS-CoV-2[tiab] OR coronavirus[tiab] OR "long COVID"[tiab] OR "post-acute COVID"[tiab]) AND ("Executive Function"[Mesh] OR "Cognition"[Mesh] OR "Neuropsychological Tests"[Mesh] OR "Cognition Disorders"[Mesh]OR "executive function*"[tiab] OR "cognitive control"[tiab] OR "working memory"[tiab] OR "cognitive inhibition"[tiab] OR "cognitive flexibility"[tiab] OR "cognitive performance"[tiab])

Scopus TITLE-ABS-KEY(teacher* OR professor* OR educator*

OR instructor* OR tutor* OR lecturer* OR faculty) AND

TITLE-ABS-KEY("COVID-19" OR "SARS-CoV-2" OR coronavirus OR "long COVID" OR "post-acute COVID") AND TITLE-ABS-KEY("executive function*" OR "cognitive control" OR "working memory" OR "cognitive inhibition"

OR "cognitive flexibility"OR "cognitive performance")

Web of Science TS=(teacher* OR professor* OR educator* OR instructor* OR tutor* OR lecturer* OR faculty) AND TS=("COVID-19" OR "SARS-CoV-2" OR coronavirus OR "long COVID" OR "post-acute COVID") AND TS=("executive function*" OR "cognitive control" OR "working memory" OR "cognitive inhibition" OR "cognitive flexibility"OR "cognitive performance")

EMBASE ('school teacher'/exp OR 'faculty member'/exp OR teacher*:ti,ab OR professor*:ti,ab OR educator*:ti,ab OR instructor*:ti,ab OR tutor*:ti,ab OR lecturer*:ti,ab) AND('covid-19'/exp OR 'sars-related coronavirus 2'/exp OR 'post-acute covid-19 syndrome'/exp OR covid-19:ti,ab OR sars-cov-2:ti,ab OR coronavirus:ti,ab OR 'long covid':ti,ab) AND('executive function'/exp OR 'cognition'/exp OR 'neuropsychological test'/exp OR 'cognitive defect'/exp

OR 'working memory'/exp OR 'cognitive control':ti,ab OR 'cognitive flexibility':ti,ab OR 'cognitive inhibition':ti,ab OR 'cognitive performance':ti,ab)

ERIC (teacher* OR professor* OR educator* OR instructor* OR tutor* OR lecturer* OR faculty) AND ("COVID-19" OR "SARS-CoV-2" OR coronavirus OR "long COVID" OR "post-acute COVID") AND ("executive function*" OR "cognitive control" OR "working memory" OR "cognitive inhibition" OR "cognitive flexibility" OR "cognitive performance")

SPORTDiscus (teacher* OR professor* OR educator* OR instructor* OR tutor* OR lecturer* OR faculty) AND ("COVID-19" OR "SARS-CoV-2" OR coronavirus OR "long COVID" OR "post-acute COVID") AND ("executive function*" OR "cognitive control" OR "working memory" OR "cognitive inhibition" OR "cognitive flexibility" OR "cognitive performance")

LILACS (teacher* OR professor* OR educator* OR instructor* OR tutor* OR lecturer* OR faculty) AND ("COVID-19" OR "SARS-CoV-2" OR coronavirus OR "long COVID" OR "post-acute COVID") AND ("executive function*" OR "cognitive control" OR "working memory" OR "cognitive inhibition" OR "cognitive flexibility" OR "cognitive performance")

Reviewer #5:

Dear authors,

Thank you for this revised version for partially addressing my comments. I focus my review this time on the search strategies. There are some mistakes, and all search strategies need revising by a medical librarian or seek any international service (e.g., systematicreviews.org). The MeSH term "teachers" does not exist. The MeSH term"COVID-19" is not enough to capture all potentially relevant evidence (e.g., "Post-Acute COVID-19 Syndrome"[Mesh] OR "SARS-CoV-2"[Mesh]). What about "Cognition Disorders"[Mesh]?. The table 1 in page 7 has not changed from previous version. In the asbtract, you state where you search PubMed interface. for Medline only or all for non-Medline. Or you search Medline in another platform as Medline Ovid. All these terms are not similar, and it is important for reproducibility issues.

Authors’ Response: Thank you for this important methodological observation. We have revised all search strategies to ensure correct use of controlled vocabulary and improved sensitivity. Specifically, we replaced the non-existent MeSH term “teachers” with the valid terms “School Teachers” and “Faculty”; expanded the COVID-19 block to include “SARS-CoV-2” and “Post-Acute COVID-19 Syndrome”; and refined the cognition block by incorporating additional relevant MeSH terms. We also clarified that MEDLINE will be searched via the PubMed interface to ensure reproducibility. The updated, fully detailed strategies are now presented in the appendix. Please, see as follow:

Database Search strategy

MEDLINE (via PubMed) ("School Teachers"[Mesh] OR "Faculty"[Mesh] OR teacher*[tiab] OR professor*[tiab] OR educator*[tiab]

OR instructor*[tiab] OR tutor*[tiab] OR lecturer*[tiab] OR faculty[tiab]) AND ("COVID-19"[Mesh] OR "SARS-CoV-2"[Mesh] OR "Post-Acute COVID-19 Syndrome"[Mesh] OR COVID-19[tiab] OR SARS-CoV-2[tiab] OR coronavirus[tiab] OR "long COVID"[tiab] OR "post-acute COVID"[tiab])

AND ("Executive Function"[Mesh] OR "Cognition"[Mesh] OR "Neuropsychological Tests"[Mesh] OR "Cognition Disorders"[Mesh]OR "executive function*"[tiab] OR "cognitive control"[tiab] OR "working memory"[tiab] OR "cognitive inhibition"[tiab] OR "cognitive flexibility"[tiab] OR "cognitive performance"[tiab])

EMBASE ('school teacher'/exp OR 'faculty member'/exp OR teacher*:ti,ab OR professor*:ti,ab OR educator*:ti,ab OR instructor*:ti,ab OR tutor*:ti,ab OR lecturer*:ti,ab)

AND('covid-19'/exp OR 'sars-related coronavirus 2'/exp OR 'post-acute covid-19 syndrome'/exp OR covid-19:ti,ab OR sars-cov-2:ti,ab OR coronavirus:ti,ab OR 'long covid':ti,ab)

AND('executive function'/exp OR 'cognition'/exp OR 'neuropsychological test'/exp OR 'cognitive defect'/exp

OR 'working memory'/exp OR 'cognitive control':ti,ab OR 'cognitive flexibility':ti,ab OR 'cognitive inhibition':ti,ab OR 'cognitive performance':ti,ab)

Scopus TITLE-ABS-KEY(teacher* OR professor* OR educator*

OR instructor* OR tutor* OR lecturer* OR faculty) AND

TITLE-ABS-KEY("COVID-19" OR "SARS-CoV-2" OR coronavirus OR "long COVID" OR "post-acute COVID")

AND TITLE-ABS-KEY("executive function*" OR "cognitive control" OR "working memory" OR "cognitive inhibition"

OR "cognitive flexibility"OR "cognitive performance")

Web of Science TS=(teacher* OR professor* OR educator* OR instructor* OR tutor* OR lecturer* OR faculty) AND TS=("COVID-19" OR "SARS-CoV-2" OR coronavirus OR "long COVID" OR "post-acute COVID") AND TS=("executive function*" OR "cognitive control" OR "working memory" OR "cognitive inhibition"

OR "cognitive flexibility"OR "cognitive performance")

Cochrane Library ([mh "School Teachers"] OR [mh "Faculty"] OR teacher*:ti,ab,kw OR professor*:ti,ab,kw OR educator*:ti,ab,kw OR instructor*:ti,ab,kw OR tutor*:ti,ab,kw OR lecturer*:ti,ab,kw) AND ([mh "COVID-19"] OR [mh "SARS-CoV-2"] OR [mh "Post-Acute COVID-19 Syndrome"] OR "COVID-19":ti,ab,kw OR "SARS-CoV-2":ti,ab,kw OR coronavirus:ti,ab,kw OR "long COVID":ti,ab,kw) AND ([mh "Executive Function"] OR [mh "Cognition"] OR [mh "Neuropsychological Tests"] OR "executive function*":ti,ab,kw OR "working memory":ti,ab,kw OR "cognitive control":ti,ab,kw

OR "cognitive flexibility":ti,ab,kw)

ERIC (teacher* OR professor* OR educator* OR instructor* OR tutor* OR lecturer* OR faculty) AND

("COVID-19" OR "SARS-CoV-2" OR coronavirus OR "long COVID" OR "post-acute COVID") AND ("executive function*" OR "cognitive control" OR "working memory" OR "cognitive inhibition" OR "cognitive flexibility" OR "cognitive performance")

SPORTDiscus (teacher* OR professor* OR educator* OR instructor* OR tutor* OR lecturer* OR faculty) AND ("COVID-19" OR "SARS-CoV-2" OR coronavirus OR "long COVID" OR "post-acute COVID") AND ("executive function*" OR "cognitive control"

OR "working memory" OR "cognitive inhibition" OR "cognitive flexibility" OR "cognitive performance")

LILACS (teacher* OR professor* OR educator* OR instructor* OR tutor* OR lecturer* OR faculty) AND ("COVID-19" OR "SARS-CoV-2" OR coronavirus OR "long COVID" OR "post-acute COVID") AND ("executive function*" OR "cognitive control" OR "working memory" OR "cognitive inhibition" OR "cognitive flexibility" OR "cognitive performance")

Attachment Submitted filename: Reviewers response 04.03.docx

# Peer review: Decision Letter 4

**DOI:** 10.1371/journal.pone.0350156.r009

**Authors:** Steve Zimmerman

29 May 2026

Dear Dr. Rezende,

Thank you for submitting your manuscript to PLOS ONE. After careful consideration, we feel that it has merit but does not fully meet PLOS ONE’s publication criteria as it currently stands. Therefore, we invite you to submit a revised version of the manuscript that addresses the points raised during the review process.

The manuscript has been thoroughly evaluated by one reviewer, whose comments are available below.

Please submit your revised manuscript by Jul 12 2026 11:59PM. If you will need more time than this to complete your revisions, please reply to this message or contact the journal office at plosone@plos.org. When you're ready to submit your revision, log on to https://www.editorialmanager.com/pone/ and select the 'Submissions Needing Revision' folder to locate your manuscript file.

- A letter that responds to each point raised by the academic editor and reviewer(s). You should upload this letter as a separate file labeled 'Response to Reviewers'.
- A marked-up copy of your manuscript that highlights changes made to the original version. You should upload this as a separate file labeled 'Revised Manuscript with Track Changes'.
- An unmarked version of your revised paper without tracked changes. You should upload this as a separate file labeled 'Manuscript'.

If you would like to make changes to your financial disclosure, please include your updated statement in your cover letter. Guidelines for resubmitting your figure files are available below the reviewer comments at the end of this letter.

If applicable, we recommend that you deposit your laboratory protocols in protocols.io to enhance the reproducibility of your results. Protocols.io assigns your protocol its own identifier (DOI) so that it can be cited independently in the future. For instructions see: https://journals.plos.org/plosone/s/submission-guidelines#loc-laboratory-protocols. Additionally, PLOS ONE offers an option for publishing peer-reviewed Lab Protocol articles, which describe protocols hosted on protocols.io. Read more information on sharing protocols at https://plos.org/protocols?utm_medium=editorial-email&utm_source=authorletters&utm_campaign=protocols.

As the corresponding author, your ORCID iD is verified in the submission system and will appear in the published article. PLOS supports the use of ORCID, and we encourage all coauthors to register for an ORCID iD and use it as well. Please encourage your coauthors to verify their ORCID iD within the submission system before final acceptance, as unverified ORCID iDs will not appear in the published article. *Only*  the individual author can complete the verification step; PLOS staff *cannot*  verify ORCID iDs on behalf of authors.

We look forward to receiving your revised manuscript.

Kind regards,

Steve Zimmerman, PhD

Senior Editor, PLOS One

Journal Requirements:

If the reviewer comments include a recommendation to cite specific previously published works, please review and evaluate these publications to determine whether they are relevant and should be cited. There is no requirement to cite these works unless the editor has indicated otherwise.

[Note: HTML markup is below. Please do not edit.]

Reviewers' comments:

Reviewer's Responses to Questions

**Comments to the Author**

1. Does the manuscript provide a valid rationale for the proposed study, with clearly identified and justified research questions?

Reviewer #7: Yes

**********

2. Is the protocol technically sound and planned in a manner that will lead to a meaningful outcome and allow testing the stated hypotheses?

Reviewer #7: Partly

**********

3. Is the methodology feasible and described in sufficient detail to allow the work to be replicable?

Reviewer #7: Yes

**********

4. Have the authors described where all data underlying the findings will be made available when the study is complete?

The PLOS Data policy

Reviewer #7: Yes

**********

5. Is the manuscript presented in an intelligible fashion and written in standard English?

Reviewer #7: Yes

**********

Please use the space provided to explain your answers to the questions above and, if applicable, provide comments about issues authors must address before this protocol can be accepted for publication. You may also include additional comments for the author, including concerns about research or publication ethics.

You may also provide optional suggestions and comments to authors that they might find helpful in planning their study.

Reviewer #7: I would like to thank the esteemed editor for the invitation and the opportunity to review this manuscript.

This study addresses an important and timely topic, the impact of COVID-19 on educators’ executive functions, and the registration of the protocol in PROSPERO, as well as the use of the PRISMA-P framework, are notable strengths. However, significant methodological and reporting issues exist. Therefore, minor revisions are required before the manuscript can be considered for publication.

1- Title: The title is representative of the study design.

2- Abstract: The abstract is relatively acceptable in terms of overall structure and content, but there is a fundamental flaw in how the objective is stated. The objective of a protocol should not be "to conduct a systematic review" (explore the effects), but rather to describe the methodology and study criteria; this flaw in the abstract needs to be corrected.

3- Introduction: The introduction section is acceptable in terms of overall coverage of the topic, but it has weaknesses in conceptual precision, clarity of the research gap. The text uses terms such as "educational educators," "teachers," and "professors" but does not provide an operational or clear definition of this population (e.g., educational level, type of employment, gender, work experience). This ambiguity will cause problems later in the study's eligibility criteria. The introduction states that there are three core executive functions: inhibitory control, working memory, and cognitive flexibility. However, when elaborating on each, it only provides examples for working memory and cognitive flexibility, giving only a brief explanation for inhibitory control. Additionally, it mentions "planning" and "decision-making" as more complex skills but does not explain their relationship with the three core functions. There is no clarity in distinguishing between the direct effects of the virus and the indirect effects of the pandemic. The introduction discusses both neuronal damage caused by the virus (inflammation, hypoxia) and the stress, anxiety, and workload resulting from the pandemic, but it does not differentiate whether the impairment in teachers' executive functions is due to the infection itself or the psychosocial conditions of the pandemic. This confounding is a fundamental weakness in the study's design. The research gap is not explicitly stated. Although at the end it says "no systematic review has addressed this issue," it does not explain why, despite previous reviews on the general population, a specific review focused on teachers is needed. The differences between teachers and other professions (e.g., unique cognitive load, continuous interaction with students, high flexibility requirements) are not mentioned. There is inconsistency between the objectives and the research question. The research question is: "Does COVID-19 affect teachers' executive functions in the long term?" But the objective of the protocol is: "to describe the methodology... to investigate the consequences of infection" (not to answer the question). This confusion between the protocol and the study itself is unacceptable. Furthermore, the term "long term" is not defined in the introduction (how many months? which indicator?).

The authors mention four previous reviews (35-38) but do not critique any of them. For example, the methodological flaws of those reviews or why their results are not generalizable to teachers are not mentioned. The explanation of neural mechanisms (demyelination, inflammation) is repeated across several paragraphs. Some sentences, such as "These skills are important for teaching activities," appear more than once with similar phrasing. Important confounding variables are not mentioned. Factors such as infection severity (hospitalized or outpatient), vaccination, history of neurological diseases, age, gender, and occupational stress level, which could modify the effect of COVID-19 on executive functions, are overlooked in the introduction. The writing requires editing.

4- Methods: The Methods section is generally well‑designed, with just a few minor issues that should be addressed. The exclusion criterion that removes individuals diagnosed with psychiatric illnesses introduces selection bias, because COVID-19 itself can cause psychiatric disorders such as depression, anxiety, and PTSD; excluding these individuals means only a subgroup of infected persons without psychiatric symptoms is included, which biases the estimated effect of COVID‑19 on executive functions, and these characteristics would be better treated as moderating variables rather than exclusion criteria. Similarly, the exclusion of studies that rely on self‑reported executive functioning creates a high risk of an empty review, since many pandemic‑era studies have used self‑report questionnaires like the BRIEF‑A or simple surveys; if only studies employing objective neuropsychological tests (e.g., Wisconsin Card Sorting Test, Stroop, N‑back) are accepted, it is very likely that no eligible studies will be found. Furthermore, no operational definition of "educator" is provided in the eligibility criteria: the population is defined only as "adults working as teachers/educators at any educational level," with no criteria to distinguish educational level (preschool, primary, secondary, university), type of employment (full‑time, part‑time), or minimum work experience, leading to high population heterogeneity that will be difficult to manage in a quantitative synthesis. The protocol also states a threshold of 70% for low risk of bias without citing any reference or methodological justification for this cutoff. In the text, references are made to Table 1 and Table 2, but Table 3 appears without a citation after the description of qualitative synthesis. Finally, although the research question and introduction use the term "long‑term," the methods section does not define a minimum follow‑up period (e.g., 3 months, 6 months, 1 year), so studies with different follow‑up durations will be included without stratification.

5- Results: Not Applicable

6- Tables: Tables present the key information in a structured format.

7- Figures: The PRISMA figure was appropriate in terms of standards.

8- Discussion and Limitation: The Discussion section is not appropriate for a systematic review protocol, as it discusses expected findings rather than methodological limitations. In a systematic review protocol, before the search and data analysis are carried out, a "Discussion" section does not conceptually make sense. Protocols typically use a "Discussion" or "Considerations" section to address the methodological challenges ahead, not to provide insights based on results. In this text, the Discussion is written as if findings already exist (e.g., "This protocol provides critical insights"). This is premature for a protocol and constitutes unscientific exaggeration. The Discussion contains unsubstantiated claims lacking supporting references, such as: "This protocol provides critical insights that can inform the development of interventions..." while no data yet exist. Critical methodological limitations are not properly identified or analyzed. The limitations mentioned in the Discussion are very superficial and general: the limitation of "a limited number of studies" is noted, but its consequences (e.g., inability to perform meta‑analysis, lack of generalizability) are not addressed. For the limitation of "population heterogeneity," the proposed solution ("subgroup classification") is overly simplistic, and real statistical challenges (e.g., insufficient number of studies within subgroups) are ignored. Important limitations not mentioned in the Discussion include, for example, the high risk of an empty review due to restrictive eligibility criteria (excluding self‑report and psychiatric illness). Sentences such as "Executive functions are fundamental to an educator's professional performance" and "This field is still poorly explored" are repeated from the introduction. Instead of repeating, the Discussion should focus on the challenges facing the protocol. A good Discussion in a protocol should honestly acknowledge the weaknesses of the study design, for example: excluding self‑report studies may lead to loss of real-world evidence; excluding individuals with psychiatric illness may introduce selection bias; the likely absence of a comparison group in many studies. The section on generalizability is very brief and unclear. It states: "Our findings will be limited to educators and cannot be generalized to all populations." However, it does not explain why educators differ from other populations (e.g., due to high cognitive load, occupational stress, or frequent interaction with students). This statement is unsupported and merely a general comment.

9- Conclusion: The conclusion section contains claims about results that appear before the study has been conducted. For example, the statement "It is expected that the findings will help develop interventions..." is made even though no search, extraction, or analysis has yet been performed. In a protocol, the conclusion should be limited to methodology, not to future findings. The conclusion should briefly mention the main limitations (e.g., "This protocol may encounter a limited number of studies due to restrictive eligibility criteria"). In the current text, no limitations are mentioned.

**********

what does this mean?). If published, this will include your full peer review and any attached files.

If you choose “no”, your identity will remain anonymous but your review may still be made public.

**Do you want your identity to be public for this peer review?**  For information about this choice, including consent withdrawal, please see our Privacy Policy

Reviewer #7: No

**********

[NOTE: If reviewer comments were submitted as an attachment file, they will be attached to this email and accessible via the submission site. Please log into your account, locate the manuscript record, and check for the action link "View Attachments". If this link does not appear, there are no attachment files.]

To ensure your figures meet our technical requirements, please review our figure guidelines: https://journals.plos.org/plosone/s/figures

You may also use PLOS’s free figure tool, NAAS, to help you prepare publication quality figures: https://journals.plos.org/plosone/s/figures#loc-tools-for-figure-preparation.

NAAS will assess whether your figures meet our technical requirements by comparing each figure against our figure specifications.

# Author comment: Author response to Decision Letter 5

**DOI:** 10.1371/journal.pone.0350156.r010

30 Jul 2026

Dear reviewer, we are very grateful for your careful analysis and valuable comments. We use all of them to improve the methodological quality and scientific rigor of our work. Please see the answers to the questions below.

1- Title: The title is representative of the study design.

Authors response: Thanks for your positive feedback.

2- Abstract: The abstract is relatively acceptable in terms of overall structure and content, but there is a fundamental flaw in how the objective is stated. The objective of a protocol should not be "to conduct a systematic review" (explore the effects), but rather to describe the methodology and study criteria; this flaw in the abstract needs to be corrected.

Authors response: Thank you for this comment. We have revised the objective in the abstract to reflect the purpose of a protocol, emphasizing the methodological framework and predefined criteria for the planned systematic review and meta-analysis. See as follow:

“Objective: This study aims to present the methodological framework and predefined criteria that will guide a systematic review and meta-analysis investigating the effects of COVID-19 infection on educators’ executive functions.

3- Introduction: The introduction section is acceptable in terms of overall coverage of the topic, but it has weaknesses in conceptual precision, clarity of the research gap. The text uses terms such as "educational educators," "teachers," and "professors" but does not provide an operational or clear definition of this population (e.g., educational level, type of employment, gender, work experience). This ambiguity will cause problems later in the study's eligibility criteria.

Authors response: Thank you for this valuable comment. We intentionally adopted broad eligibility criteria for the educator population because of the limited number of studies available on this topic. Restricting participants by educational level, employment type, gender, or work experience could substantially reduce the available evidence. Therefore, our aim is to comprehensively map the existing literature across all educator groups. If sufficient data are identified, subgroup analyses based on these characteristics will be considered in the systematic review.

The introduction states that there are three core executive functions: inhibitory control, working memory, and cognitive flexibility. However, when elaborating on each, it only provides examples for working memory and cognitive flexibility, giving only a brief explanation for inhibitory control. Additionally, it mentions "planning" and "decision-making" as more complex skills but does not explain their relationship with the three core functions.

Authors response: Thank you for this comment. We have revised the description of executive functions by providing a clearer definition of inhibitory control and clarifying that higher-order abilities, such as planning and decision-making, arise from the interaction of the three core executive functions rather than constituting independent executive functions. See as follow:

“The literature has identified three central EFs (26): i) inhibitory control (i.e., the ability to suppress automatic or inappropriate responses/behaviors, resist distractions, and maintain goal-directed attention (17, 18) and maintaining selective attention (26, 27)), ii) working memory (i.e., retention and manipulation of information) (28), and ii) cognitive flexibility (i.e., adaptation to new rules (18) and perspectives (10). From these core functions, more complex skills such as planning, decision-making, problem-solving, and reasoning emerge (18). These higher-order abilities rely on the coordinated interaction of inhibitory control, working memory, and cognitive flexibility rather than representing independent executive functions.

There is no clarity in distinguishing between the direct effects of the virus and the indirect effects of the pandemic. The introduction discusses both neuronal damage caused by the virus (inflammation, hypoxia) and the stress, anxiety, and workload resulting from the pandemic, but it does not differentiate whether the impairment in teachers' executive functions is due to the infection itself or the psychosocial conditions of the pandemic. This confounding is a fundamental weakness in the study's design. The research gap is not explicitly stated.

Authors response: Thank you for this important comment. We acknowledge that distinguishing the direct neurological effects of SARS-CoV-2 infection from the indirect psychosocial consequences of the pandemic is currently a limitation of the available evidence rather than of the study design. We have clarified this point in the Introduction, emphasizing that the proposed systematic review aims to synthesize the existing evidence and identify knowledge gaps that may guide future research to better disentangle these mechanisms.

A lthough at the end it says "no systematic review has addressed this issue," it does not explain why, despite previous reviews on the general population, a specific review focused on teachers is needed. The differences between teachers and other professions (e.g., unique cognitive load, continuous interaction with students, high flexibility requirements) are not mentioned. There is inconsistency between the objectives and the research question.

Authors response: Thank you for this comment. The Introduction already includes a paragraph highlighting the importance of executive functions for educators, emphasizing the specific cognitive demands of teaching, such as multitasking, continuous information integration, responsiveness to dynamic classroom environments, and pedagogical decision-making. We have revised this section to further clarify that this rationale supports the need for a review specifically focused on educators. Please, see as follow:

“For educators, these EFs are critical to the performance of professional duties. Teaching demands the integration of information, multitasking, responsiveness to dynamic classroom environments (including online formats), and the ability to make pedagogical decisions under time constraints. Cognitive impairments in these areas may, therefore, directly compromise educational quality and teacher well-being. A report explained that COVID-19 led to impairments in executive function in 36% of cases (32). Other studies have shown similar results, indicating that COVID-19 infection may lead to disorders related to headache, confusion and attention difficulties (21, 23).”

The research question is: "Does COVID-19 affect teachers' executive functions in the long term?" But the objective of the protocol is: "to describe the methodology... to investigate the consequences of infection" (not to answer the question). This confusion between the protocol and the study itself is unacceptable.

Authors response: Thank you for this comment. We agree that the central research question is whether COVID-19 affects educators' executive functions in the long term. However, as this manuscript is a systematic review protocol, its objective is to describe the methodological procedures and predefined criteria that will guide the planned systematic review addressing this research question. We have revised the text to make this distinction clearer.

Furthermore, the term "long term" is not defined in the introduction (how many months? which indicator?).

Authors response: Thank you for this comment. We acknowledge that there is currently no consensus in the literature regarding a specific time threshold for defining long-term executive function outcomes following COVID-19 infection, particularly in educators. Therefore, we did not establish a predefined cutoff. Instead, the systematic review will synthesize the available evidence according to the follow-up periods reported in the included studies, allowing the findings to be presented and interpreted across different time intervals.

The authors mention four previous reviews (35-38) but do not critique any of them. For example, the methodological flaws of those reviews or why their results are not generalizable to teachers are not mentioned. The explanation of neural mechanisms (demyelination, inflammation) is repeated across several paragraphs. Some sentences, such as "These skills are important for teaching activities," appear more than once with similar phrasing.

Authors response: Thank you for this comment. We consider it appropriate to avoid generalizing findings from other occupational groups, as teaching involves specific cognitive demands that may influence executive functioning differently. Moreover, evidence on this topic remains limited, even in broader populations. We have clarified this rationale in the Introduction and revised the text to reduce repetitive descriptions of neural mechanisms and executive functions. We believe that the planned systematic review will help identify current evidence gaps and provide valuable information to guide future research and inform educational systems.

Important confounding variables are not mentioned. Factors such as infection severity (hospitalized or outpatient), vaccination, history of neurological diseases, age, gender, and occupational stress level, which could modify the effect of COVID-19 on executive functions, are overlooked in the introduction. The writing requires editing.

Authors response: Thank you for this valuable comment. We agree that these variables are important and may influence the relationship between COVID-19 infection and executive function outcomes. Accordingly, we have incorporated a paragraph in the Introduction acknowledging these potential confounding factors and highlighting their relevance when interpreting the available evidence. Please, see as follow:

“Multiple factors may contribute to the variability in executive function outcomes following COVID-19 infection, including infection severity, vaccination status, pre-existing neurological conditions, age, sex, and occupational stress. These variables may contribute to the heterogeneity of cognitive outcomes reported across studies and should be considered when interpreting the available evidence (39, 40). Therefore, the proposed systematic review will also seek to identify and summarize these characteristics whenever they are reported in the included studies.”

4- Methods: The Methods section is generally well designed, with just a few minor issues that should be addressed. The exclusion criterion that removes individuals diagnosed with psychiatric illnesses introduces selection bias, because COVID-19 itself can cause psychiatric disorders such as depression, anxiety, and PTSD; excluding these individuals means only a subgroup of infected persons without psychiatric symptoms is included, which biases the estimated effect of COVID 19 on executive functions, and these characteristics would be better treated as moderating variables rather than exclusion criteria. Similarly, the exclusion of studies that rely on self reported executive functioning creates a high risk of an empty review, since many pandemic era studies have used self report questionnaires like the BRIEF A or simple surveys;

Authors response: Thank you for this valuable comment. We acknowledge the reviewer's concern; however, these exclusion criteria were intentionally established to minimize potential confounding factors and improve the specificity of the review. Psychiatric disorders may independently affect executive function performance, making it difficult to isolate the association with COVID-19 infection. In addition, our objective is to synthesize evidence based on objective assessments of executive functions using validated neuropsychological instruments, such as the Five Digits Test (FDT), the Montreal Cognitive Assessment (MoCA), and the Rey Auditory Verbal Learning Test (RAVLT), rather than self-reported measures, which assess perceived cognitive functioning and are more susceptible to reporting bias. We have clarified this rationale in the manuscript.

If only studies employing objective neuropsychological tests (e.g., Wisconsin Card Sorting Test, Stroop, N back) are accepted, it is very likely that no eligible studies will be found. Furthermore, no operational definition of "educator" is provided in the eligibility criteria: the population is defined only as "adults working as teachers/educators at any educational level," with no criteria to distinguish educational level (preschool, primary, secondary, university), type of employment (full time, part time), or minimum work experience, leading to high population heterogeneity that will be difficult to manage in a quantitative synthesis.

Authors response: T hank you for this valuable comment. We appreciate the reviewer's concern; however, we have decided to maintain the inclusion of studies based on objective neuropsychological assessments. Although this approach may reduce the number of eligible studies, we believe it provides greater methodological rigor for evaluating executive functions. If no eligible studies are identified, this finding will itself highlight an important gap in the literature and reinforce the need for further research using objective cognitive assessments in educators. Regarding the definition of educators, we intentionally adopted broad eligibility criteria to maximize the identification of relevant studies in this emerging field. If sufficient data are available, participant characteristics, such as educational level, employment conditions, and workload, will be explored through subgroup or stratified analyses.

The protocol also states a threshold of 70% for low risk of bias without citing any reference or methodological justification for this cutoff.

Authors response: Thank you for alerting us to the lack of a reference for this information. The reference has been added. See below:

“Studies scoring 70% or higher will be considered to have a low risk of bias (58).”

58. Noll M, de Mendonca CR, de Souza Rosa LP, Silveira EA. Determinants of eating patterns and nutrient intake among adolescent athletes: a systematic review. Nutr J. 2017 Jul 28;16(1):46.

In the text, references are made to Table 1 and Table 2, but Table 3 appears without a citation after the description of qualitative synthesis.

Authors response: Thank you for alerting us to the missing reference to Table 3 in the text. We have made the correction. See below:

“Descriptive and content analyses of the results will also be conducted based on the impact of COVID-19 on educators’ executive functions. The extracted data will include quantitative indicators such as means, standard deviations, and effect sizes related to executive function performance (e.g., working memory, cognitive flexibility, inhibitory control), as well as qualitative themes derived from narrative reports or interviews (Tab. 3).”

Finally, although the research question and introduction use the term "long term," the methods section does not define a minimum follow up period (e.g., 3 months, 6 months, 1 year), so studies with different follow up durations will be included without stratification.

Authors response: Thank you for this valuable comment. We acknowledge that no universally accepted definition of a minimum follow-up period for long-term executive function outcomes after COVID-19 infection currently exists, particularly in studies involving educators. Therefore, we did not establish a predefined cutoff. Instead, follow-up duration will be extracted from each included study, and the findings will be stratified according to the reported follow-up periods whenever possible.

5- Results: Not Applicable.

6- Tables: Tables present the key information in a structured format.

7- Figures: The PRISMA figure was appropriate in terms of standards.

8- Discussion and Limitation: The Discussion section is not appropriate for a systematic review protocol, as it discusses expected findings rather than methodological limitations. In a systematic review protocol, before the search and data analysis are carried out, a "Discussion" section does not conceptually make sense. Protocols typically use a "Discussion" or "Considerations" section to address the methodological challenges ahead, not to provide insights based on results. In this text, the Discussion is written as if findings already exist (e.g., "This protocol provides critical insights"). This is premature for a protocol and constitutes unscientific exaggeration.

Authors response: Thank you for this thoughtful comment. We respectfully prefer to retain the Discussion section, as its inclusion is consistent with the PRISMA-P recommendations for systematic review protocols. In addition, several recently published systematic review protocols have included a Discussion section with a similar purpose. We have revised this section to ensure that it focuses on the rationale, potential contributions, and anticipated methodological considerations of the proposed review rather than on the results of the review itself. Examples of recently published protocols are provided below for reference:

https://link.springer.com/article/10.1186/2046-4053-4-1

https://bmjopen.bmj.com/content/15/5/e101478.abstract

https://bmjopen.bmj.com/content/16/5/e106406.abstract

The Discussion contains unsubstantiated claims lacking supporting references, such as: "This protocol provides critical insights that can inform the development of interventions..." while no data yet exist.

Authors response: Thank you for this valuable comment. We agree with the reviewer's observation and have revised the sentence to avoid making unsupported claims about the protocol itself. The statement now refers to the potential contributions of the future systematic review rather than the protocol, as follows:

“By systematically examining these effects, the future review can provide critical insights that can inform the development of interventions to support educators’ mental health and enhance the quality of student teaching in post-pandemic times.”

Critical methodological limitations are not properly identified or analyzed. The limitations mentioned in the Discussion are very superficial and general: the limitation of "a limited number of studies" is noted, but its consequences (e.g., inability to perform meta analysis, lack of generalizability) are not addressed. For the limitation of "population heterogeneity," the proposed solution ("subgroup classification") is overly simplistic, and real statistical challenges (e.g., insufficient number of studies within subgroups) are ignored. Important limitations not mentioned in the Discussion include, for example, the high risk of an empty review due to restrictive eligibility criteria (excluding self report and psychiatric illness).

Authors response:Thank you for this valuable comment. We appreciate the reviewer's concern; however, we respectfully prefer to maintain the current text. Although our eligibility criteria may reduce the number of eligible studies, we do not consider the possibility of an empty review to be a methodological limitation. Rather, if no eligible studies are identified, this would represent an important finding by highlighting a significant gap in the literature and the need for future research employing objective neuropsychological assessments in educators. For this reason, we have chosen not to present this possibility as a limitation of the protocol.

Sentences such as "Executive functions are fundamental to an educator's professional performance" and "This field is still poorly explored" are repeated from the introduction. Instead of repeating, the Discussion should focus on the challenges facing the protocol.

Authors response: Thank you for this valuable comment. We agree that the Discussion should avoid repeating points already presented in the Introduction. Accordingly, we have revised this section to reduce redundancy and place greater emphasis on the methodological considerations and challenges of the proposed systematic review, as shown below.

A good Discussion in a protocol should honestly acknowledge the weaknesses of the study design, for example: excluding self report studies may lead to loss of real-world evidence; excluding individuals with psychiatric illness may introduce selection bias; the likely absence of a comparison group in many studies. The section on generalizability is very brief and unclear. It states: "Our findings will be limited to educators and cannot be generalized to all populations." However, it does not explain why educators differ from other populations (e.g., due to high cognitive load, occupational stress, or frequent interaction with students). This statement is unsupported and merely a general comment.

Authors response:Thank you for this valuable comment. We appreciate the reviewer's observation. The rationale for focusing specifically on educators and the unique cognitive demands of the teaching profession has already been addressed in the Introduction. Therefore, we prefer to avoid repeating these points in the Discussion and maintain the current structure to minimize redundancy across sections. See as follow:

“For educators, these EFs are critical to the performance of professional duties. Teaching demands the integration of information, multitasking, responsiveness to dynamic classroom environments (including online formats), and the ability to make pedagogical decisions under time constraints. Cognitive impairments in these areas may, therefore, directly compromise educational quality and teacher well-being. A report explained that COVID-19 led to impairments in executive function in 36% of cases (32). Other studies have shown similar results, indicating that COVID-19 infection may lead to disorders related to headache, confusion and attention difficulties (21, 23).”

9- Conclusion: The conclusion section contains claims about results that appear before the study has been conducted. For example, the statement "It is expected that the findings will help develop interventions..." is made even though no search, extraction, or analysis has yet been performed. In a protocol, the conclusion should be limited to methodology, not to future findings. The conclusion should briefly mention the main limitations (e.g., "This protocol may encounter a limited number of studies due to restrictive eligibility criteria"). In the current text, no limitations are mentioned.

Authors response: Thank you for this valuable comment. We agree that the conclusion of a systematic review protocol should focus on the methodological aspects of the study rather than anticipated findings. Accordingly, we have revised the Conclusion to emphasize the purpose of the protocol and its methodological framework, while also acknowledging the main potential limitations of the planned review, as shown below.

“This protocol establishes the methodological framework and predefined eligibility criteria for a systematic review and meta-analysis investigating the effects of COVID-19 infection on educators’ executive functions. By providing a transparent and reproducible methodology, this protocol aims to support the rigorous synthesis of the available evidence and identify current knowledge gaps on this topic. Potential limitations include the limited number of eligible studies and the heterogeneity of the available evidence, which may affect the scope of the planned quantitative synthesis.”

Attachment Submitted filename: Reviewers response 10.07.docx

# Peer review: Decision Letter 5

**DOI:** 10.1371/journal.pone.0350156.r011

**Authors:** Gustavo De Conti Teixeira Costa

25 Aug 2026

Consequences of COVID-19 infection on educators’ executive functions: a systematic review and meta-analysis protocol

PONE-D-25-03730R5

Dear Dr. Rezende,

We’re pleased to inform you that your manuscript has been judged scientifically suitable for publication and will be formally accepted for publication once it meets all outstanding technical requirements.

Within one week, you’ll receive an e-mail detailing the required amendments. When these have been addressed, you’ll receive a formal acceptance letter and your manuscript will be scheduled for publication.

An invoice will be generated when your article is formally accepted. Please note, if your institution has a publishing partnership with PLOS and your article meets the relevant criteria, all or part of your publication costs will be covered. Please make sure your user information is up-to-date by logging into Editorial Manager at Editorial Manager® and clicking the ‘Update My Information' link at the top of the page. For questions related to billing, please contact billing support.

If your institution or institutions have a press office, please notify them about your upcoming paper to help maximize its impact. If they’ll be preparing press materials, please inform our press team as soon as possible -- no later than 48 hours after receiving the formal acceptance. Your manuscript will remain under strict press embargo until 2 pm Eastern Time on the date of publication. For more information, please contact onepress@plos.org.

Kind regards,

Gustavo De Conti Teixeira Costa, Ph.D

Academic Editor

PLOS One

Additional Editor Comments (optional):

Dear Authors,

Thank you for submitting your manuscript. Following five rounds of peer review involving seven reviewers, we are pleased to inform you that the manuscript has been judged scientifically suitable for publication and is accepted, subject to the completion of any outstanding technical requirements.

We appreciate your careful and constructive responses throughout the review process and congratulate you on the acceptance of your manuscript.

Best Regards,

Gustavo De Conti

Reviewers' comments:

Reviewer's Responses to Questions

**Comments to the Author**

1. Does the manuscript provide a valid rationale for the proposed study, with clearly identified and justified research questions?

Reviewer #7: Partly

**********

2. Is the protocol technically sound and planned in a manner that will lead to a meaningful outcome and allow testing the stated hypotheses?

Reviewer #7: Partly

**********

3. Is the methodology feasible and described in sufficient detail to allow the work to be replicable?

Reviewer #7: Yes

**********

4. Have the authors described where all data underlying the findings will be made available when the study is complete?

The PLOS Data policy

Reviewer #7: Yes

**********

5. Is the manuscript presented in an intelligible fashion and written in standard English?

Reviewer #7: Yes

**********

Please use the space provided to explain your answers to the questions above and, if applicable, provide comments about issues authors must address before this protocol can be accepted for publication. You may also include additional comments for the author, including concerns about research or publication ethics.

You may also provide optional suggestions and comments to authors that they might find helpful in planning their study.

Reviewer #7: I sincerely appreciate the authors for thoroughly addressing and responding to all the requested revisions.

**********

what does this mean?). If published, this will include your full peer review and any attached files.

If you choose “no”, your identity will remain anonymous but your review may still be made public.

**Do you want your identity to be public for this peer review?**  For information about this choice, including consent withdrawal, please see our Privacy Policy

Reviewer #7: No

**********

# Editor report: Acceptance letter

**DOI:** 10.1371/journal.pone.0350156.r012

**Authors:** Gustavo De Conti Teixeira Costa

PONE-D-25-03730R5

PLOS One

Dear Dr. Rezende,

I'm pleased to inform you that your manuscript has been deemed suitable for publication in PLOS One. Congratulations! Your manuscript is now being handed over to our production team.

At this stage, our production department will prepare your paper for publication. This includes ensuring the following:

* All references, tables, and figures are properly cited

* All relevant supporting information is included in the manuscript submission,

* There are no issues that prevent the paper from being properly typeset

You will receive further instructions from the production team, including instructions on how to review your proof when it is ready. Please keep in mind that we are working through a large volume of accepted articles, so please give us a few days to review your paper and let you know the next and final steps.

Lastly, if your institution or institutions have a press office, please let them know about your upcoming paper now to help maximize its impact. If they'll be preparing press materials, please inform our press team within the next 48 hours. Your manuscript will remain under strict press embargo until 2 pm Eastern Time on the date of publication. For more information, please contact onepress@plos.org.

You will receive an invoice from PLOS for your publication fee after your manuscript has reached the completed accept phase. If you receive an email requesting payment before acceptance or for any other service, this may be a phishing scheme. Learn how to identify phishing emails and protect your accounts at https://explore.plos.org/phishing.

If we can help with anything else, please email us at customercare@plos.org.

Thank you for submitting your work to PLOS One and supporting open access.

Kind regards,

PLOS One Editorial Office Staff

on behalf of

Dr. Gustavo De Conti Teixeira Costa

Academic Editor

PLOS One
